# Supplementary material for: p53-induced RNA-binding protein ZMAT3 inhibits transcription of a hexokinase to suppress mitochondrial respiration in human cancer cells
Source: eLife. 2026 Mar 17;14:RP107538. doi: 10.7554/eLife.107538 (PMC12995290; doi:10.7554/eLife.107538)
Supplement: Figure 1—figure supplement 1—source data 1. [file elife-107538-fig1-figsupp1-data1.zip › Figure_1-figure_supplement_1-source_data_1_.pdf]

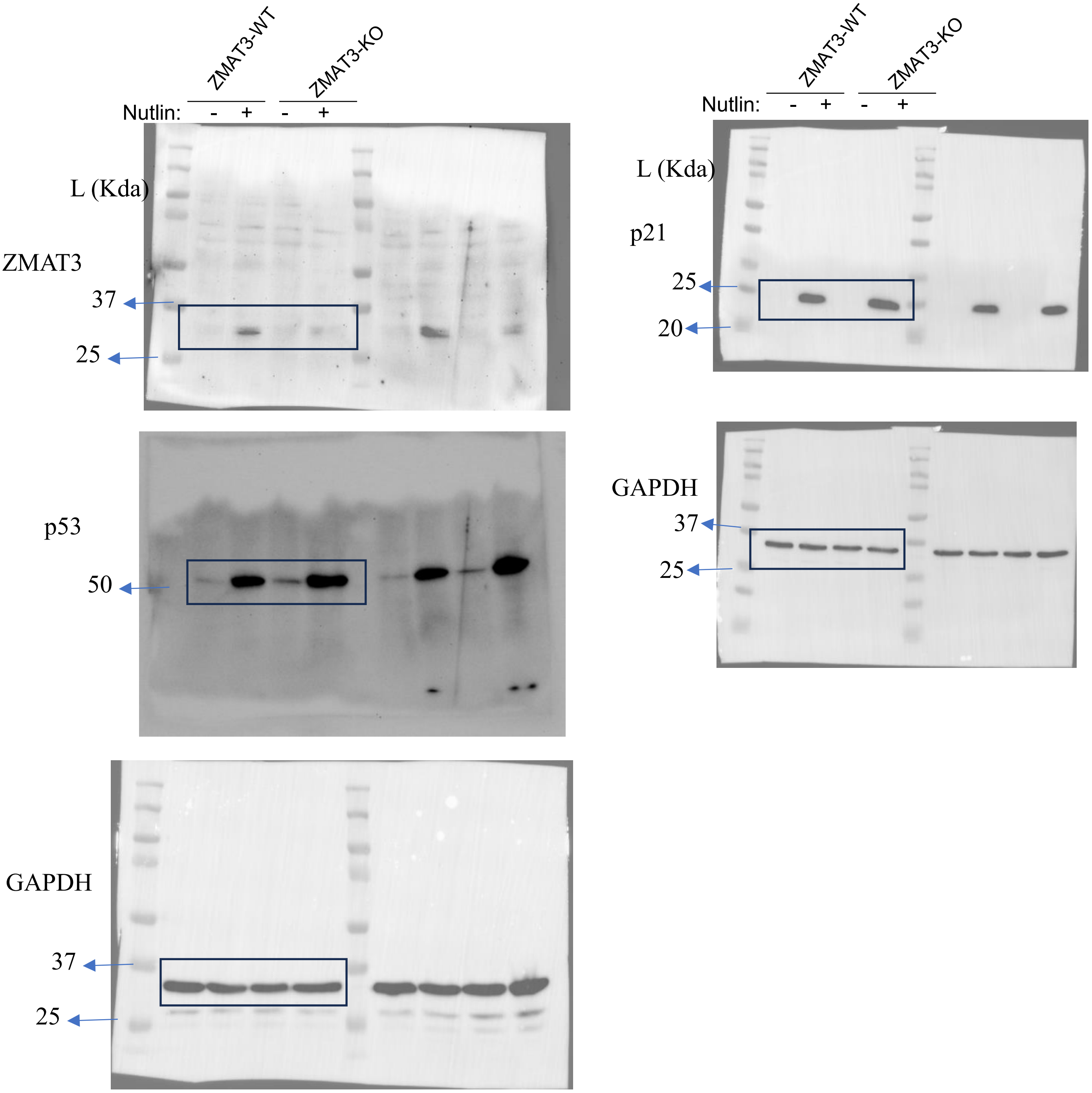

Figure 1-figure supplement 1-source data 1 . Original membranes corresponding to Figure1- figure supplement 1, panel A. BIO-RAD molecular markers (catalog no. 161-0394) were employed. The membranes correspond to ZMAT3, p53, p21 and GAPDH Immunoblot.
