# Supplementary figures and images for: p53-induced RNA-binding protein ZMAT3 inhibits transcription of a hexokinase to suppress mitochondrial respiration in human cancer cells
Source: eLife. 2026 Mar 17;14:RP107538. doi: 10.7554/eLife.107538 (PMC12995290; doi:10.7554/eLife.107538)

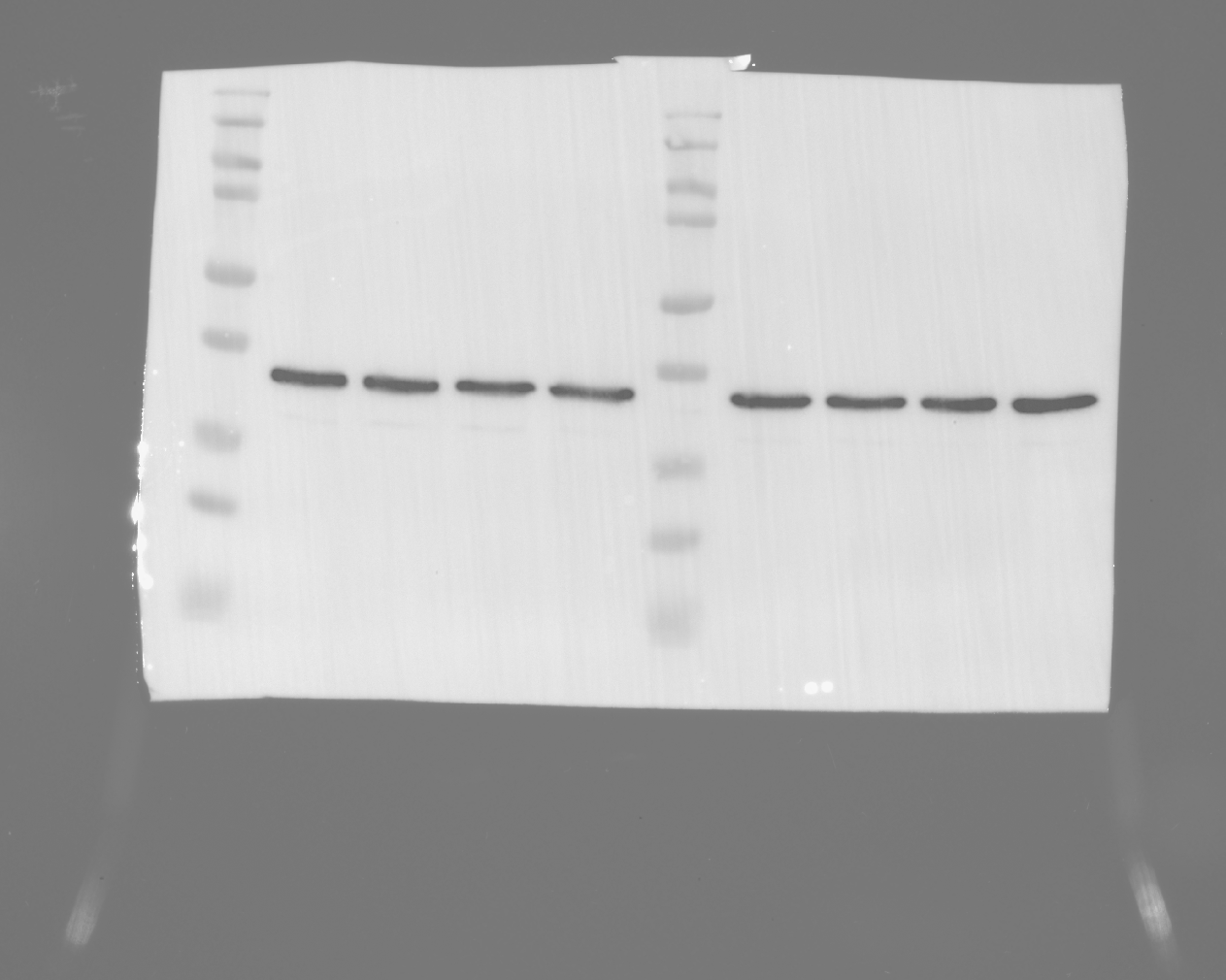

Supplement: Figure 1—figure supplement 1—source data 2. [file elife-107538-fig1-figsupp1-data2.zip › Figure 1- figure supplement 1A source data 2/GAPDH lower.tif]

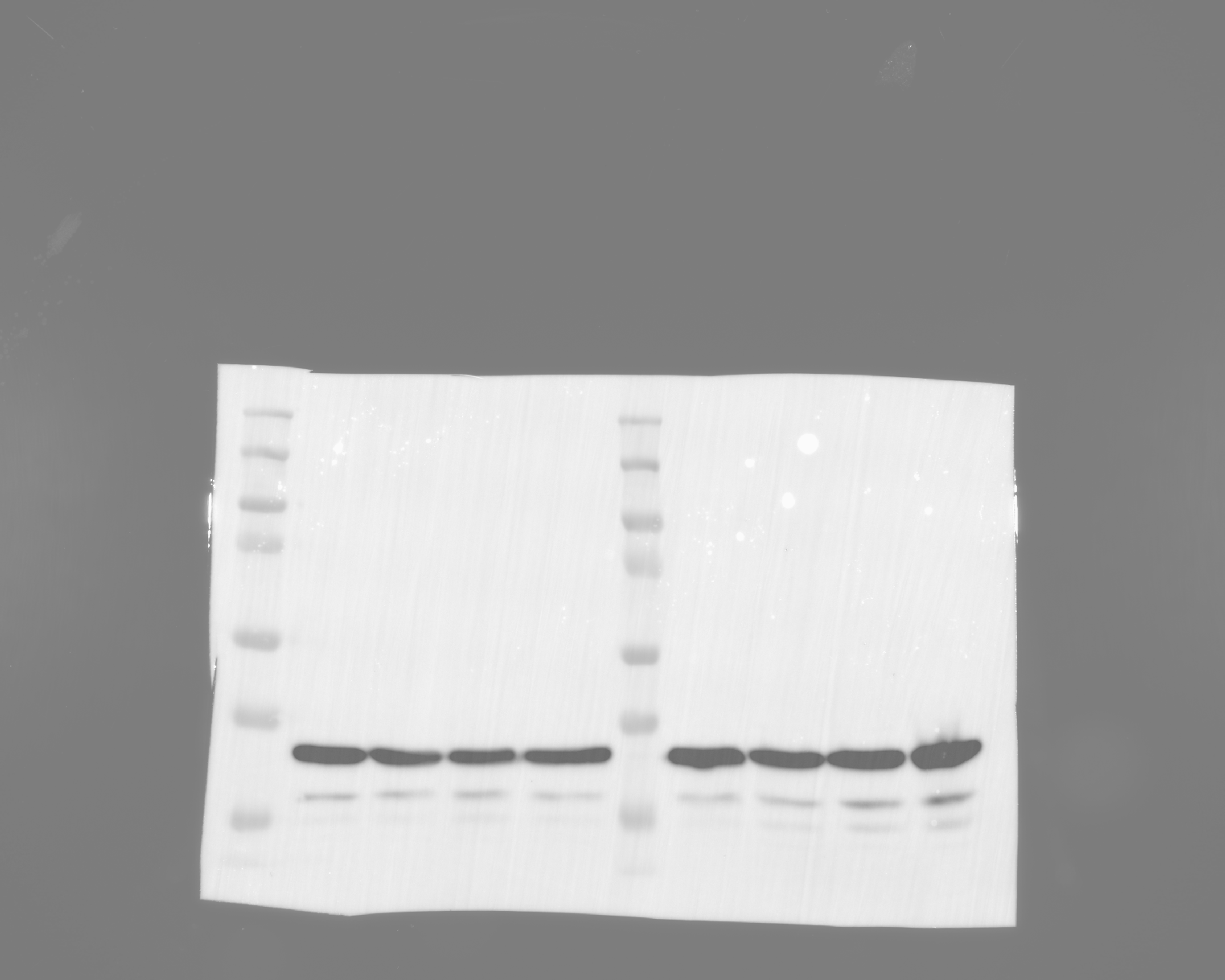

Supplement: Figure 1—figure supplement 1—source data 2. [file elife-107538-fig1-figsupp1-data2.zip › Figure 1- figure supplement 1A source data 2/GAPDH.tif]

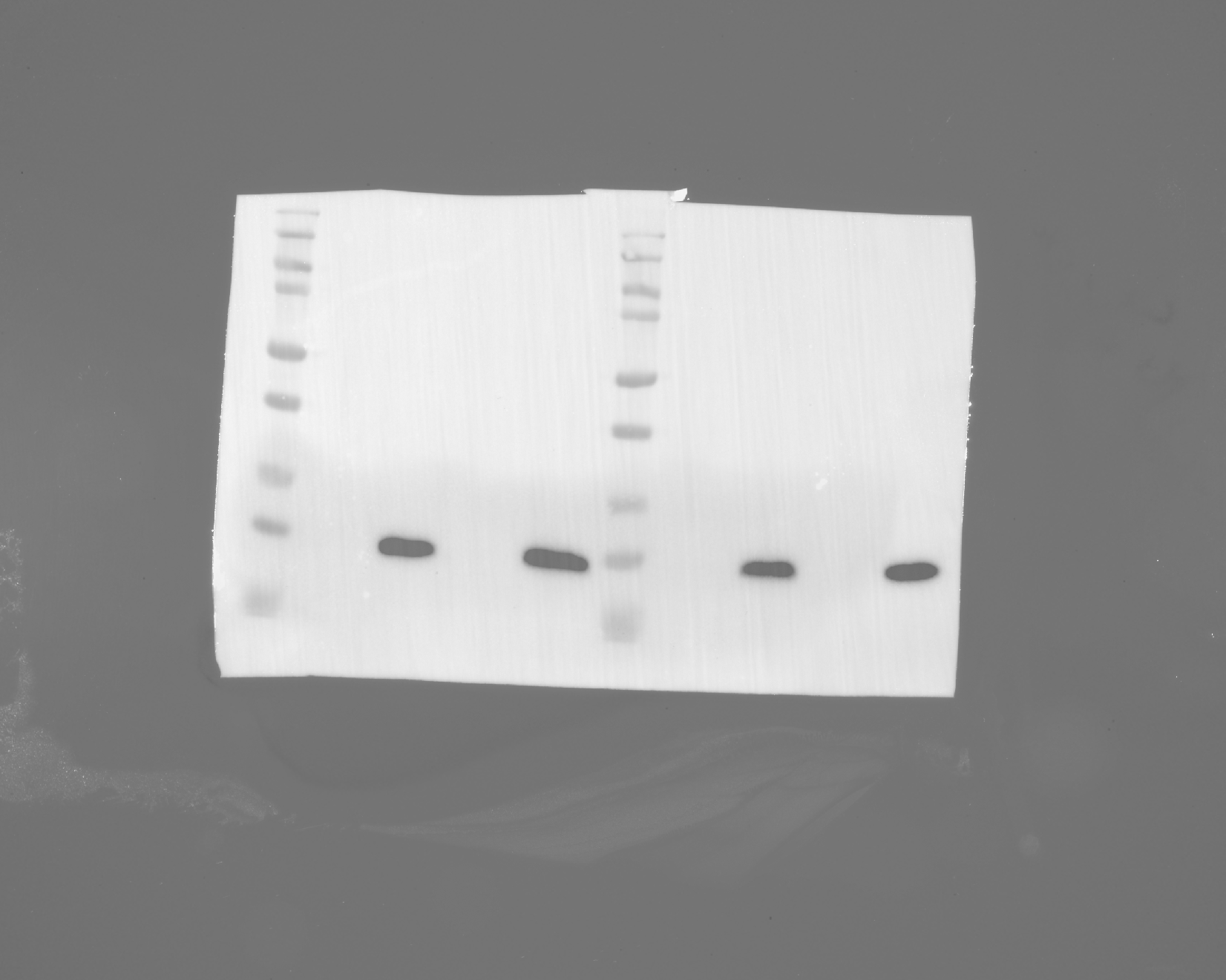

Supplement: Figure 1—figure supplement 1—source data 2. [file elife-107538-fig1-figsupp1-data2.zip › Figure 1- figure supplement 1A source data 2/p21.tif]

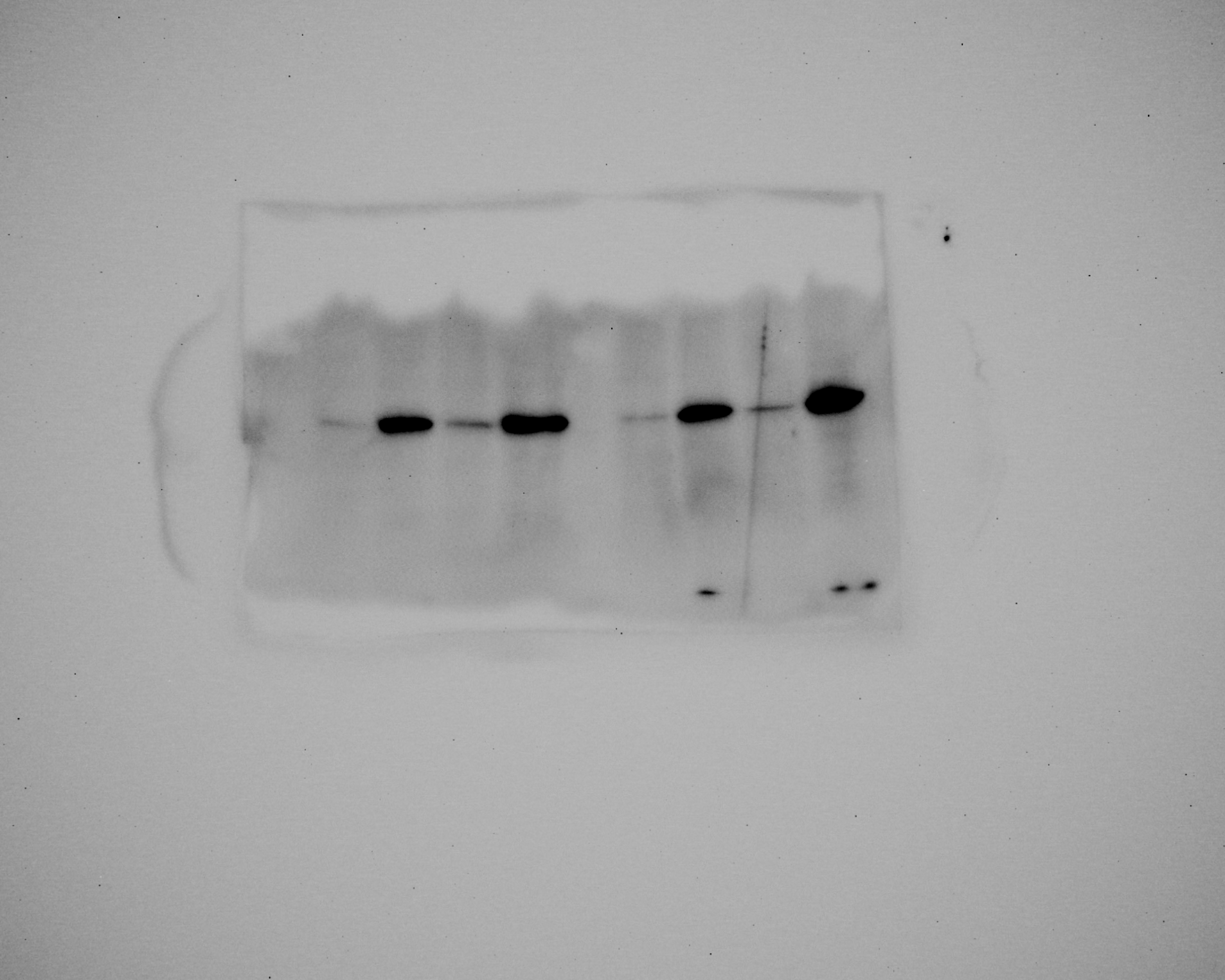

Supplement: Figure 1—figure supplement 1—source data 2. [file elife-107538-fig1-figsupp1-data2.zip › Figure 1- figure supplement 1A source data 2/p53.tif]

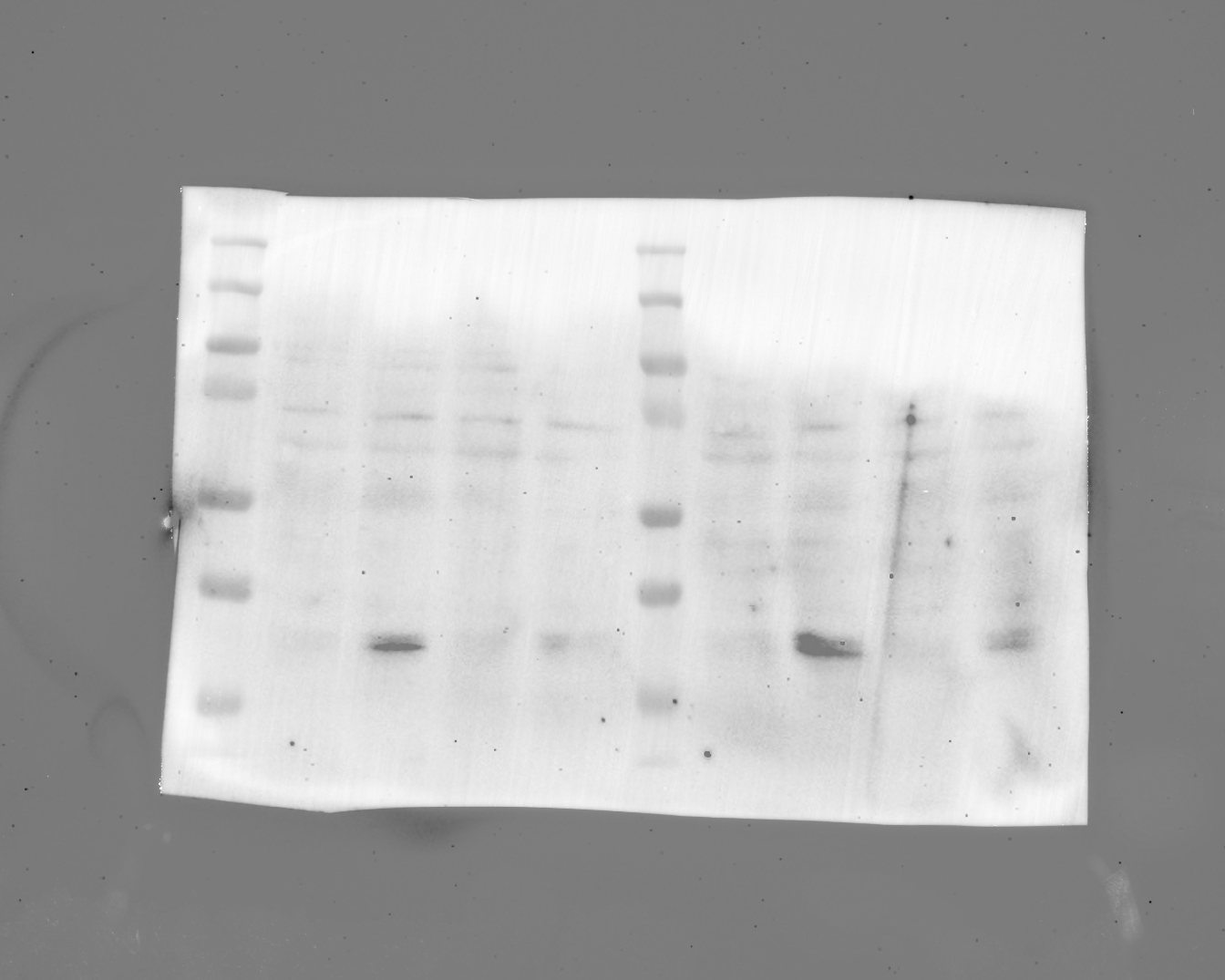

Supplement: Figure 1—figure supplement 1—source data 2. [file elife-107538-fig1-figsupp1-data2.zip › Figure 1- figure supplement 1A source data 2/ZMAT3.tif]

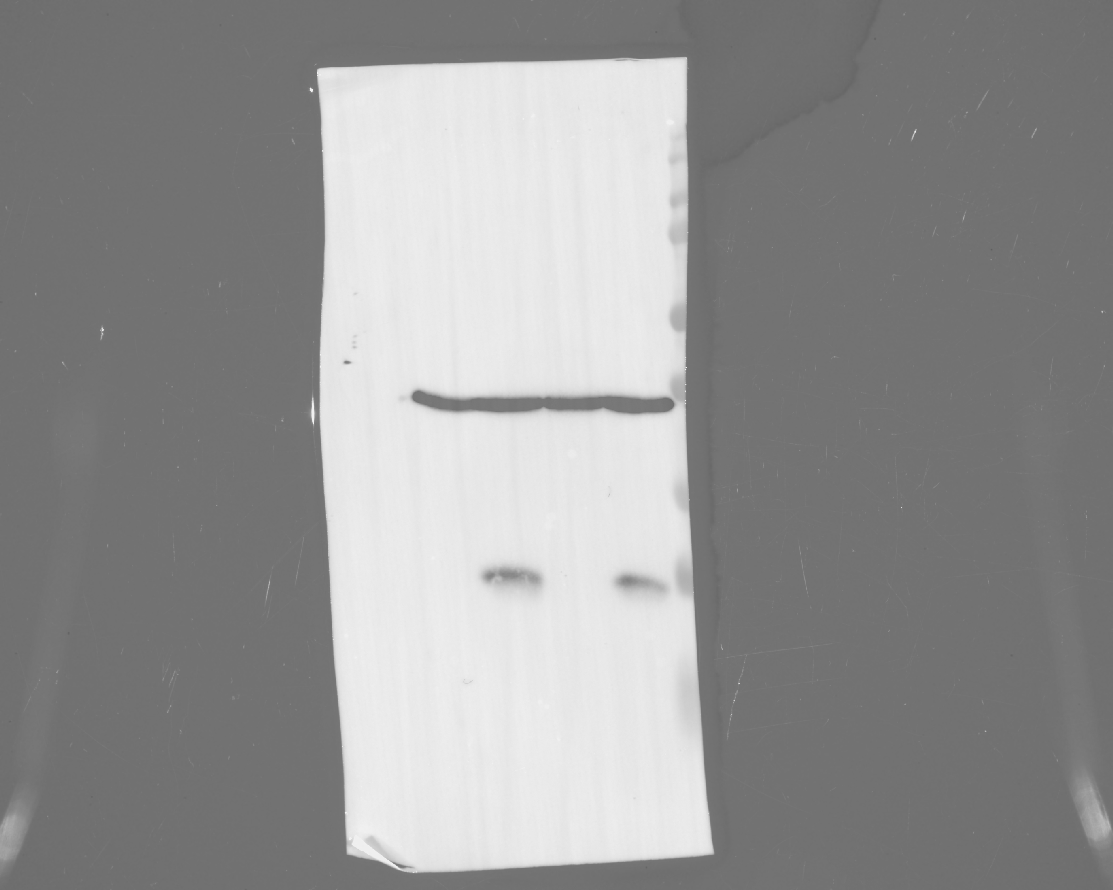

Supplement: Figure 1—figure supplement 2—source data 2. [file elife-107538-fig1-figsupp2-data2.zip › Figure 1-figure supplement 2A-source data 2/A GAPDH.tif]

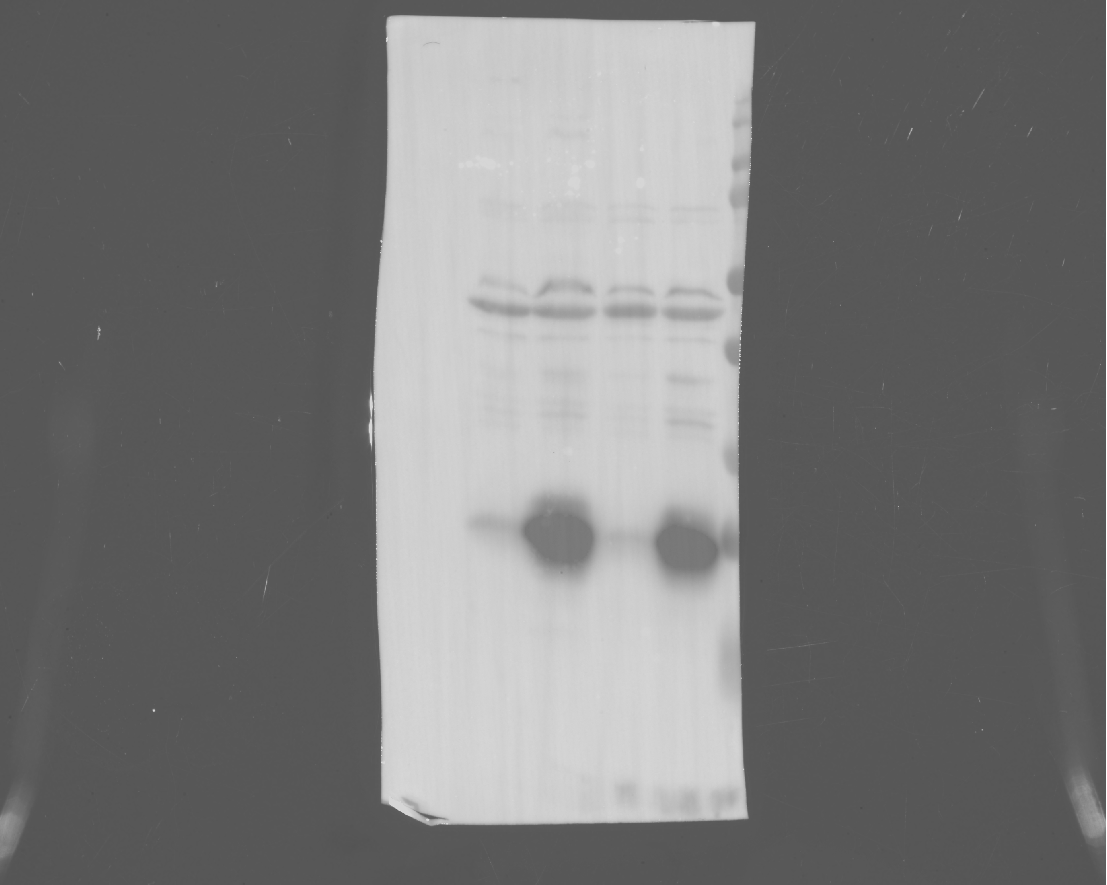

Supplement: Figure 1—figure supplement 2—source data 2. [file elife-107538-fig1-figsupp2-data2.zip › Figure 1-figure supplement 2A-source data 2/A p21.tif]

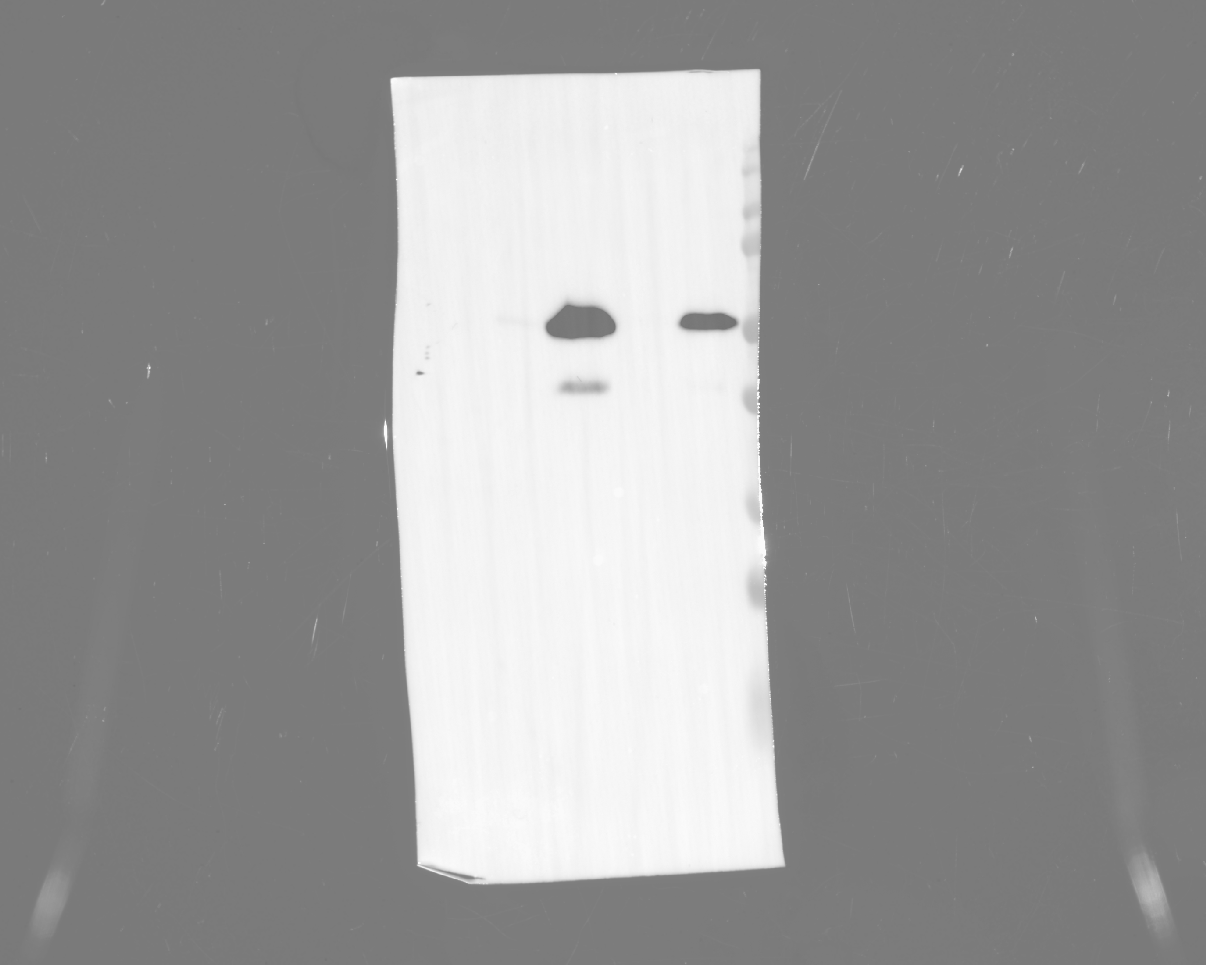

Supplement: Figure 1—figure supplement 2—source data 2. [file elife-107538-fig1-figsupp2-data2.zip › Figure 1-figure supplement 2A-source data 2/A p53.tif]

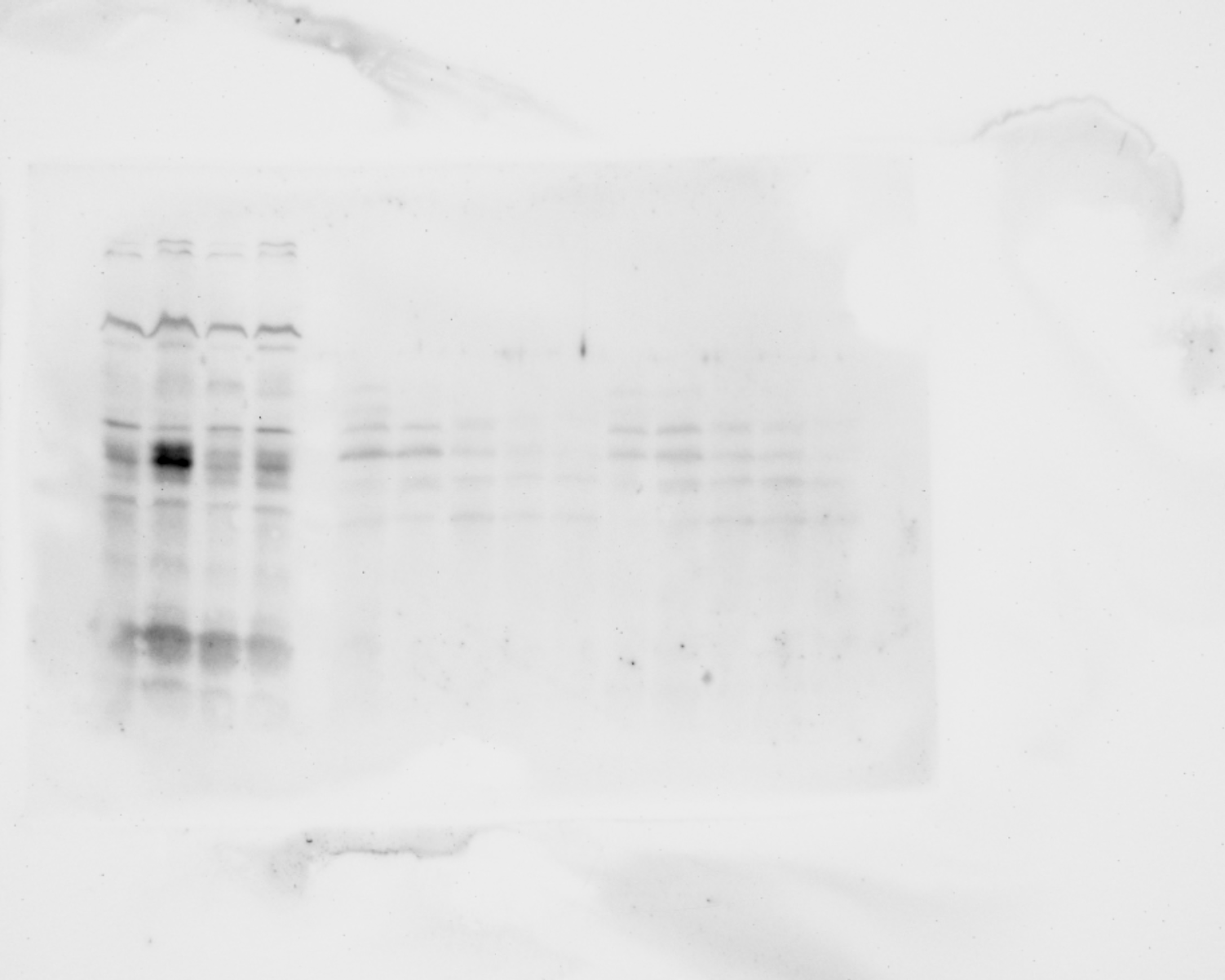

Supplement: Figure 1—figure supplement 2—source data 2. [file elife-107538-fig1-figsupp2-data2.zip › Figure 1-figure supplement 2A-source data 2/A ZMAT3.tif]

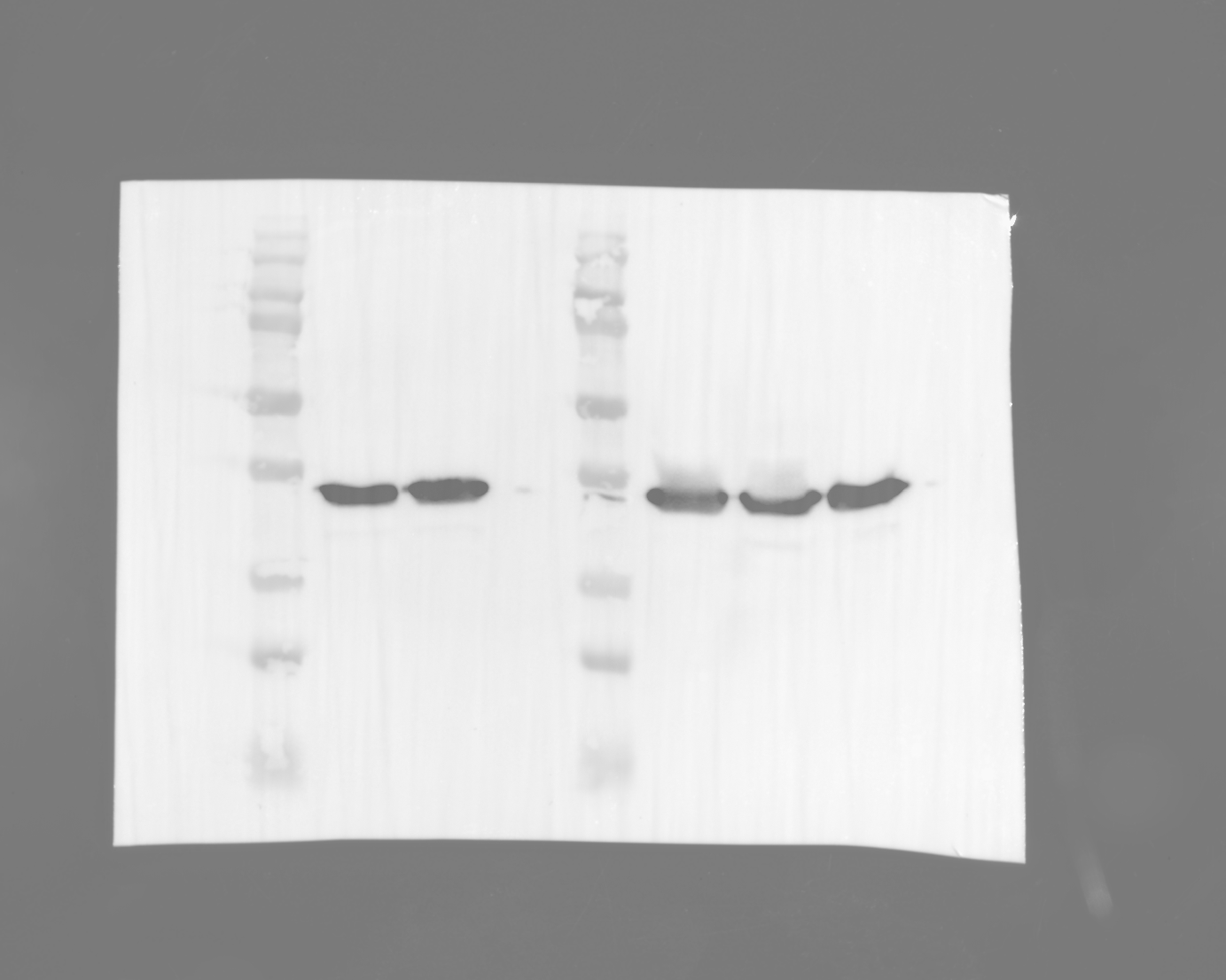

Supplement: Figure 2—source data 2. [file elife-107538-fig2-data2.zip › Figure 2B source data 2/GAPDH.tif]

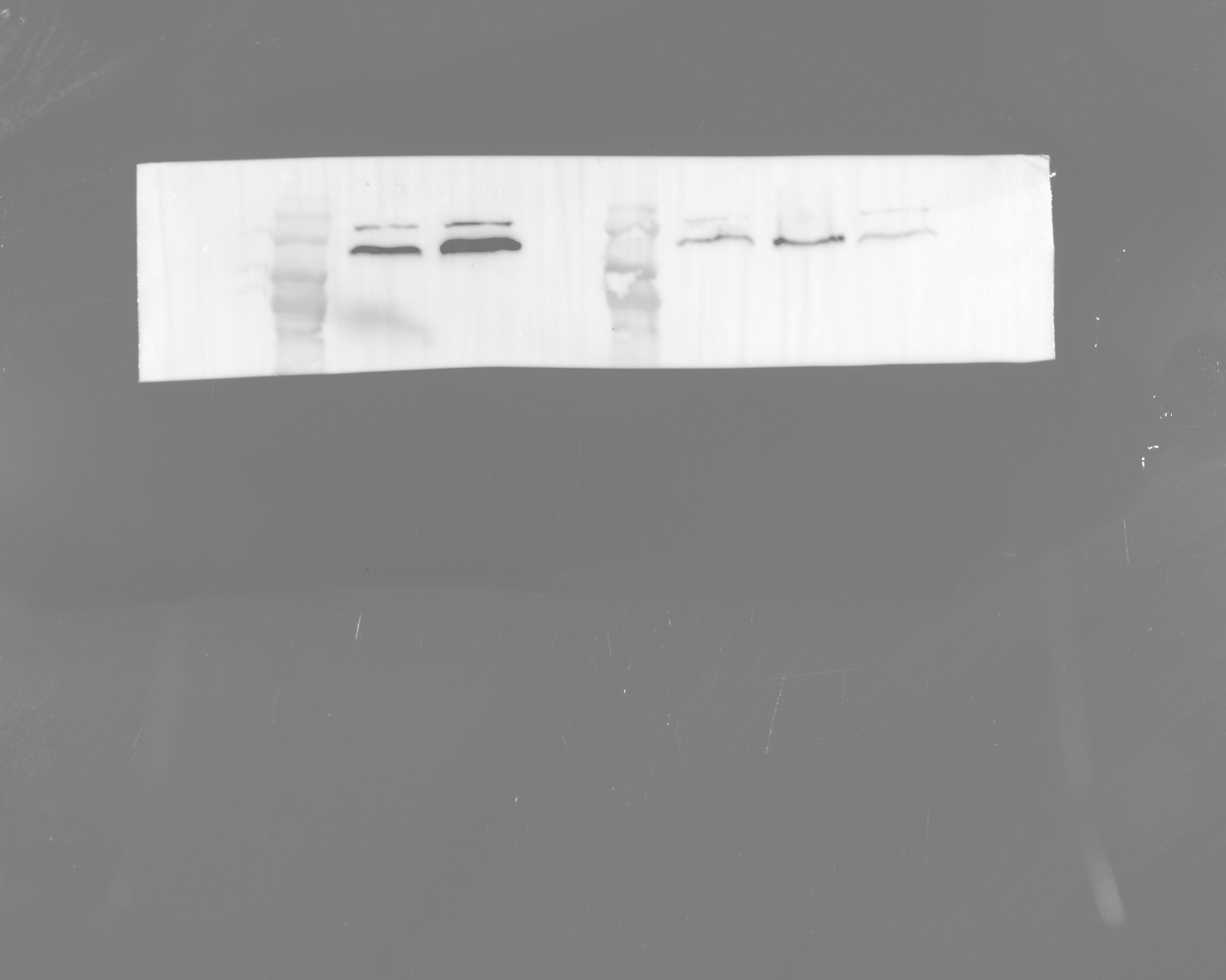

Supplement: Figure 2—source data 2. [file elife-107538-fig2-data2.zip › Figure 2B source data 2/HKDC1.tif]

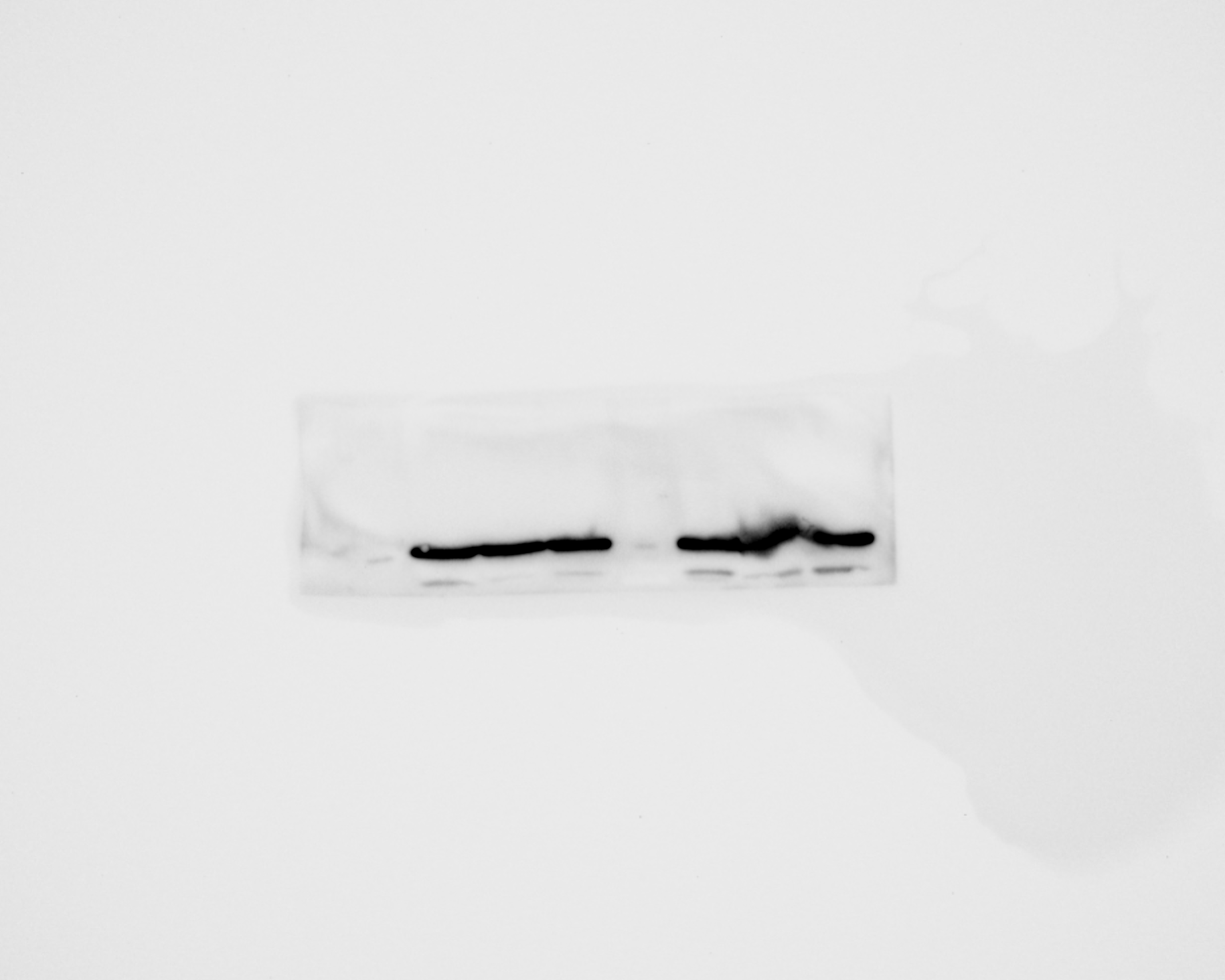

Supplement: Figure 2—source data 2. [file elife-107538-fig2-data2.zip › Figure 2E source data 2/HCT116 GAPDH.tif]

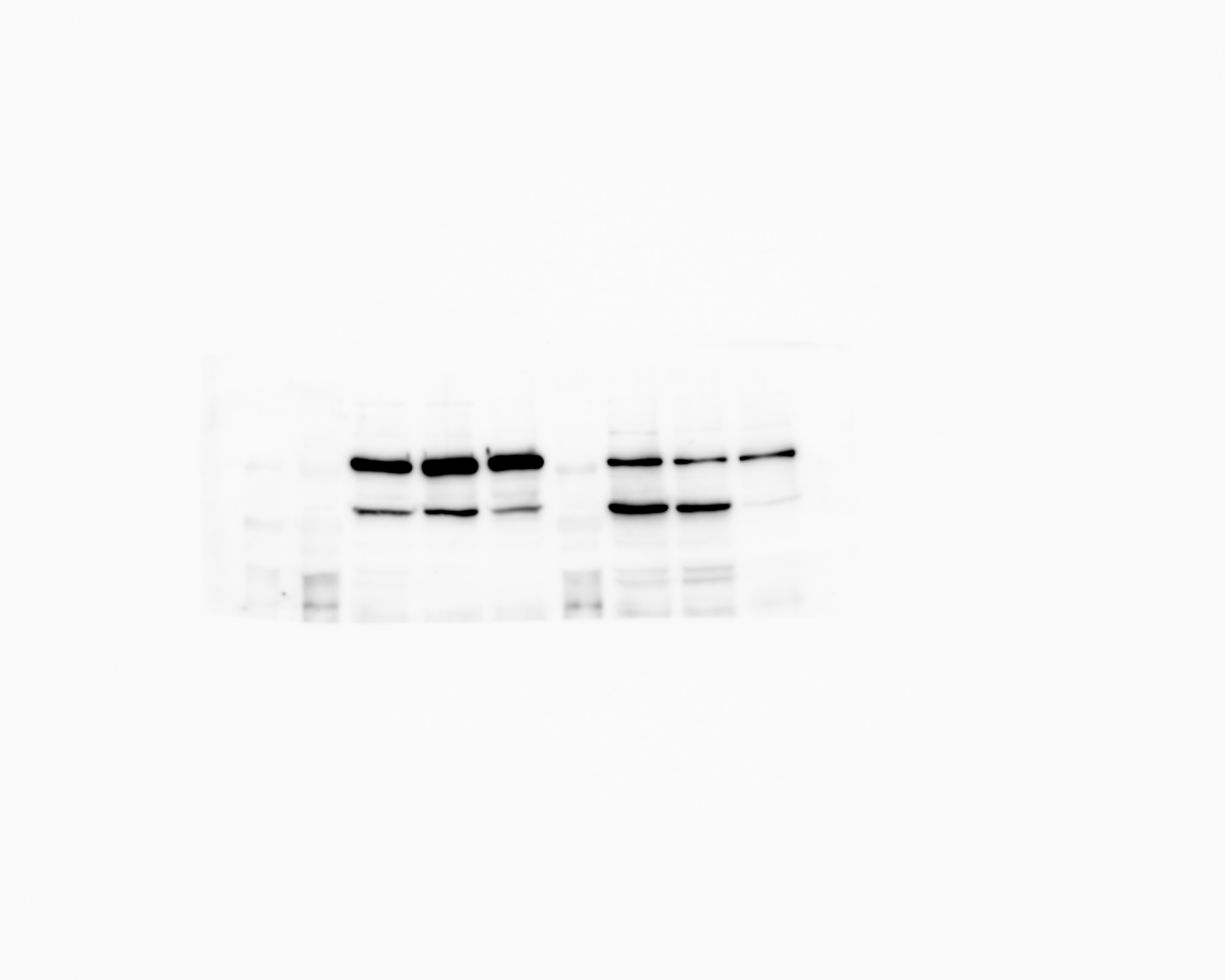

Supplement: Figure 2—source data 2. [file elife-107538-fig2-data2.zip › Figure 2E source data 2/HCT116 HKDC1.tif]

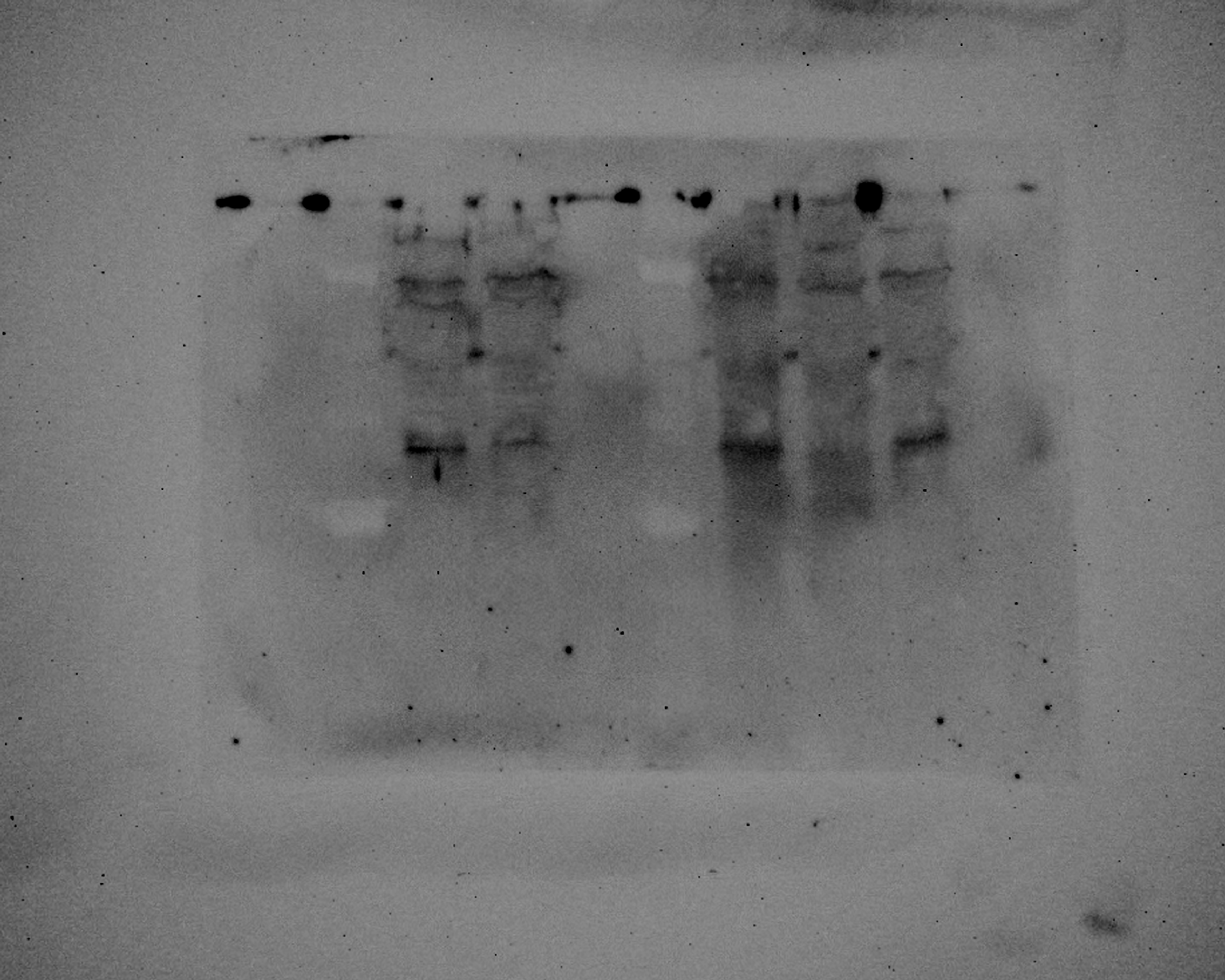

Supplement: Figure 2—source data 2. [file elife-107538-fig2-data2.zip › Figure 2E source data 2/HCT116 ZMAT3.tif]

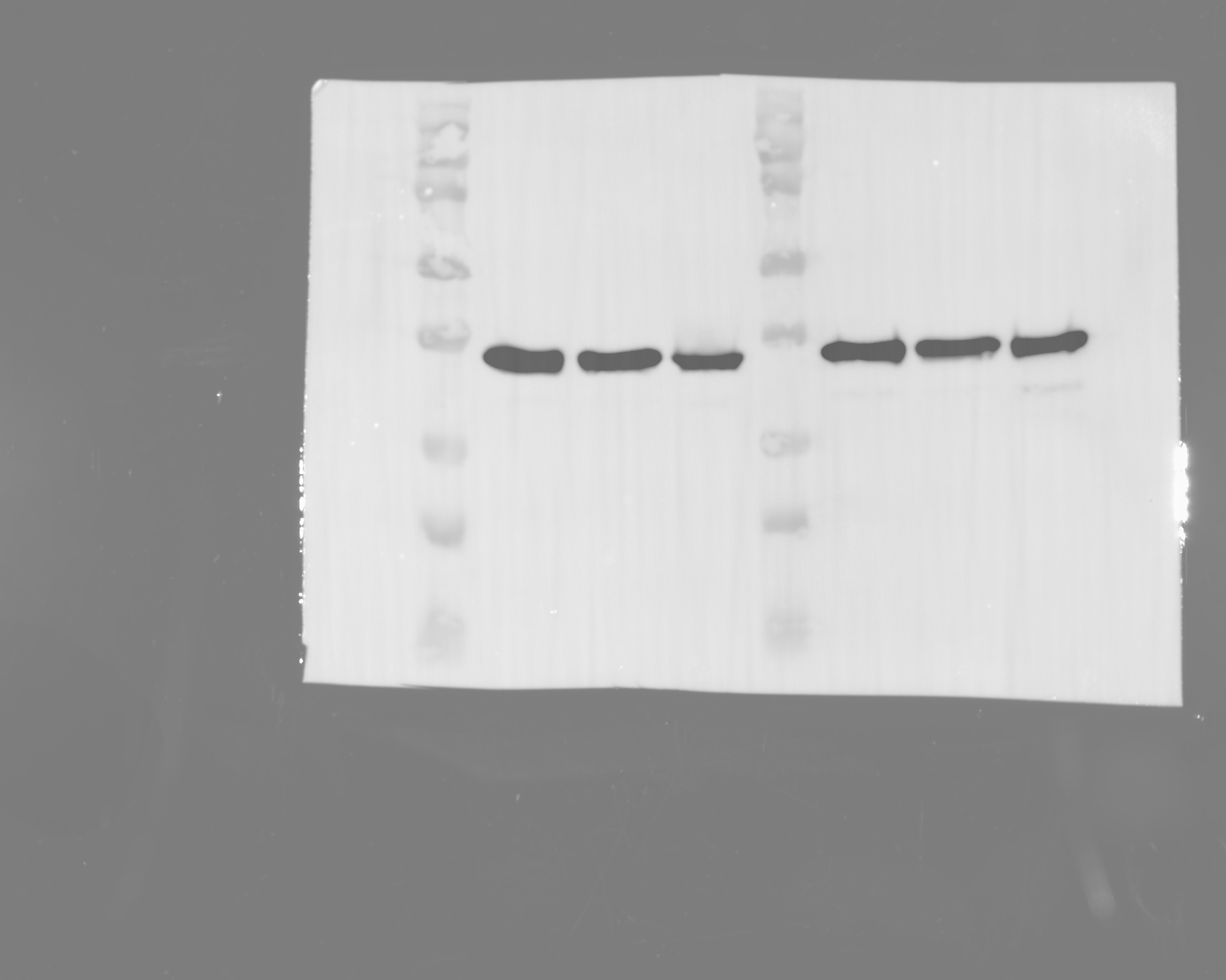

Supplement: Figure 2—source data 2. [file elife-107538-fig2-data2.zip › Figure 2E source data 2/HEPG2 GAPDH.tif]

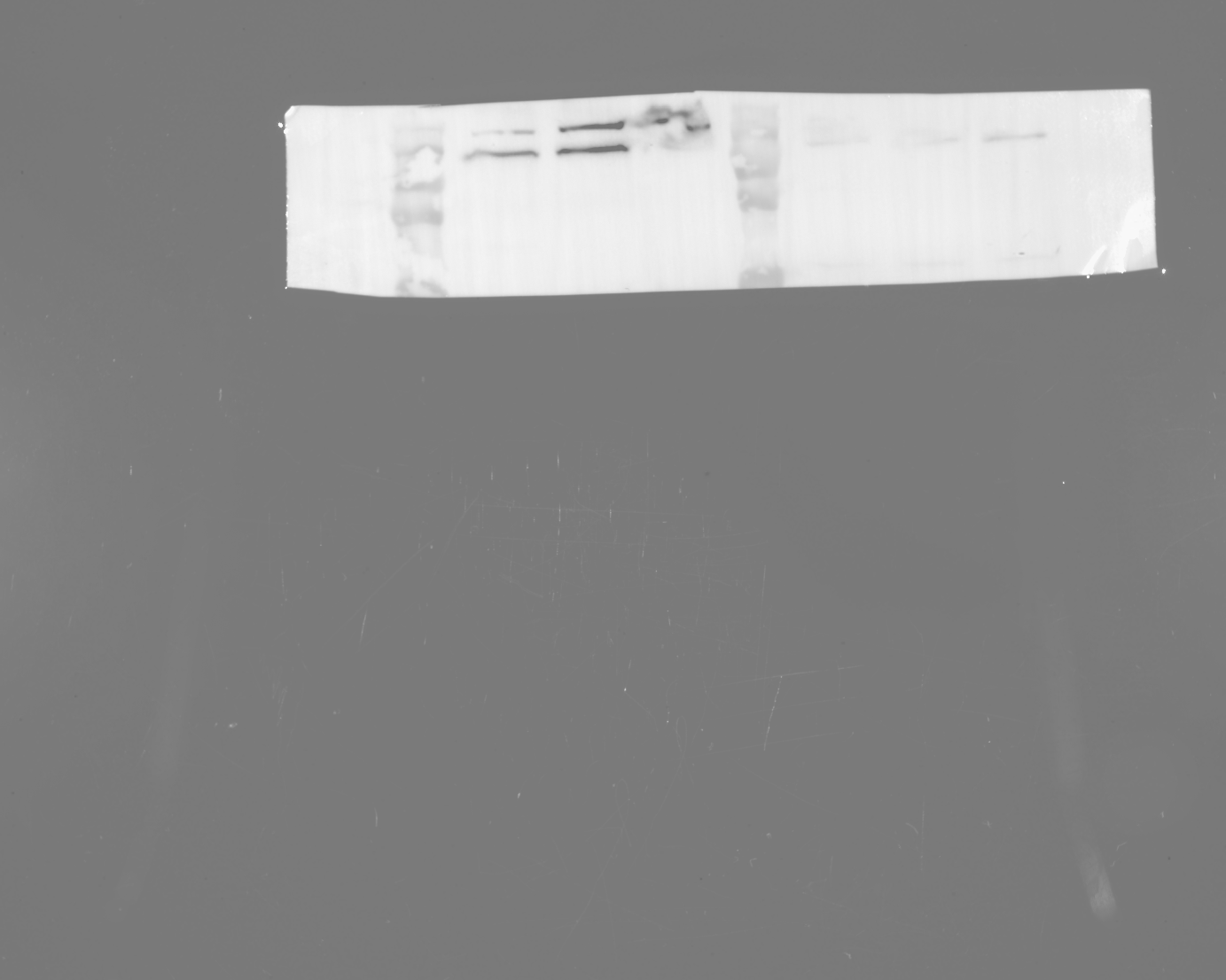

Supplement: Figure 2—source data 2. [file elife-107538-fig2-data2.zip › Figure 2E source data 2/HEPG2 HKDC1.tif]

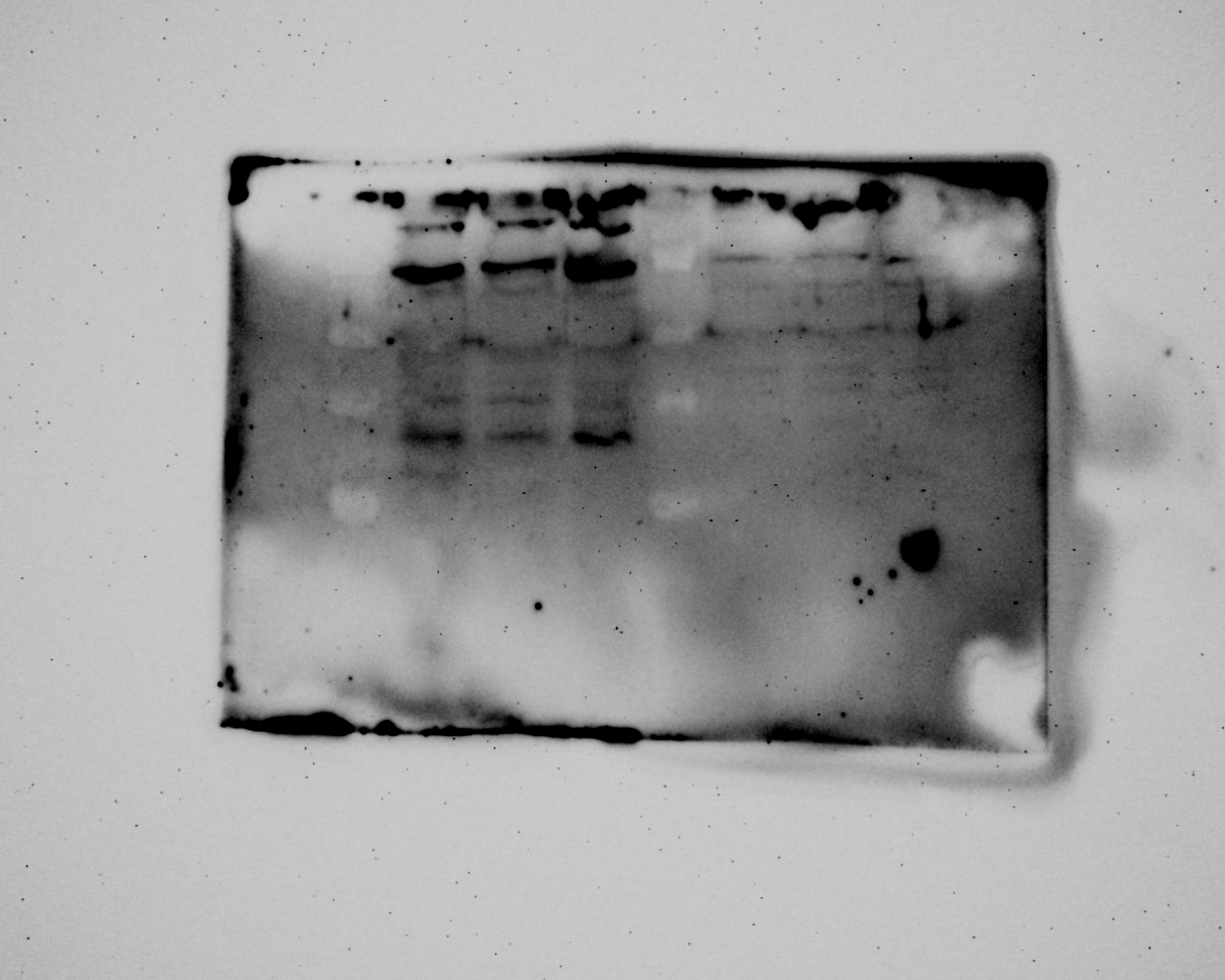

Supplement: Figure 2—source data 2. [file elife-107538-fig2-data2.zip › Figure 2E source data 2/HEPG2 ZMAT3.tif]

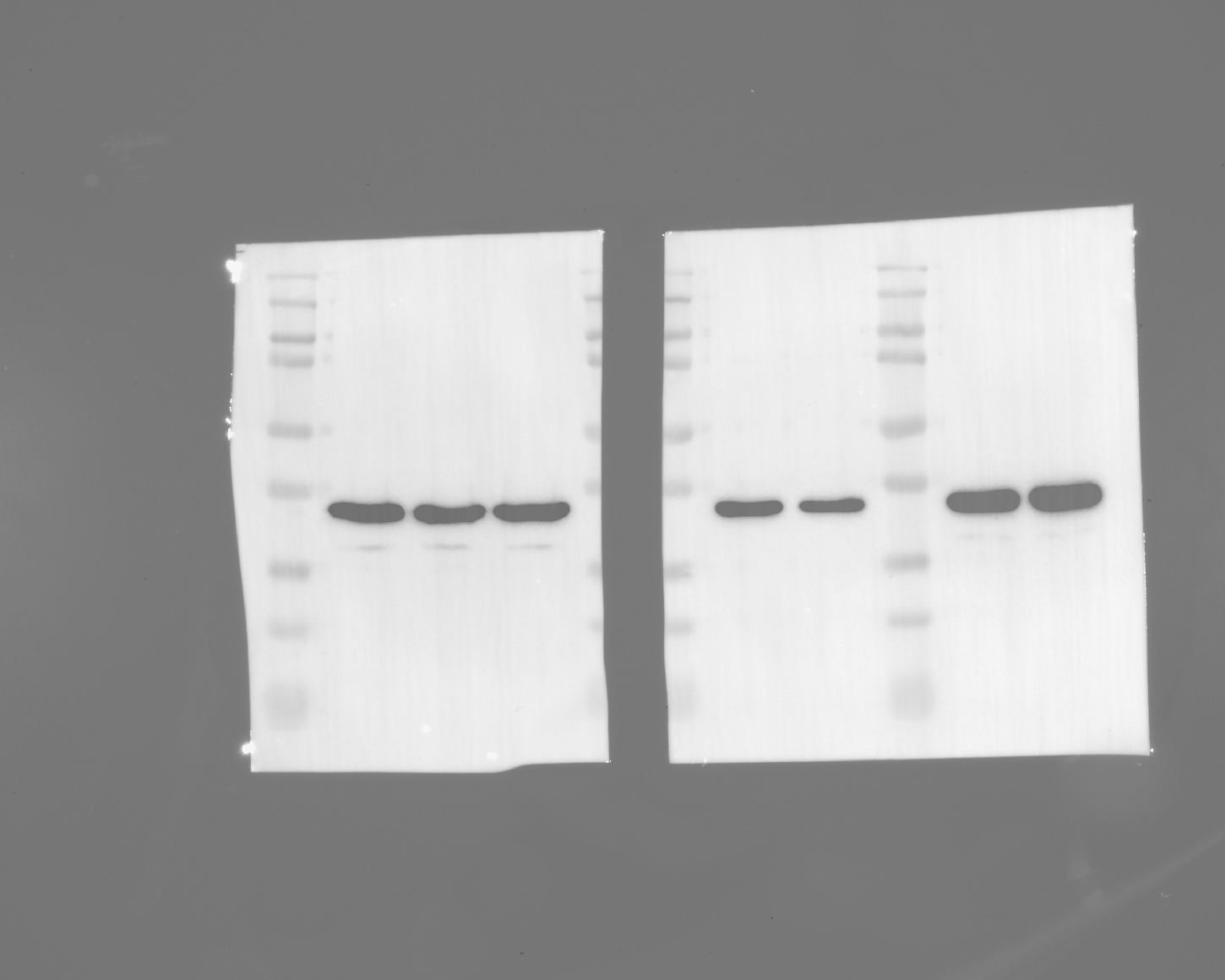

Supplement: Figure 2—figure supplement 1—source data 2. [file elife-107538-fig2-figsupp1-data2.zip › Figure 2- figure supplement 1F source data 2/GAPDH lower.tif]

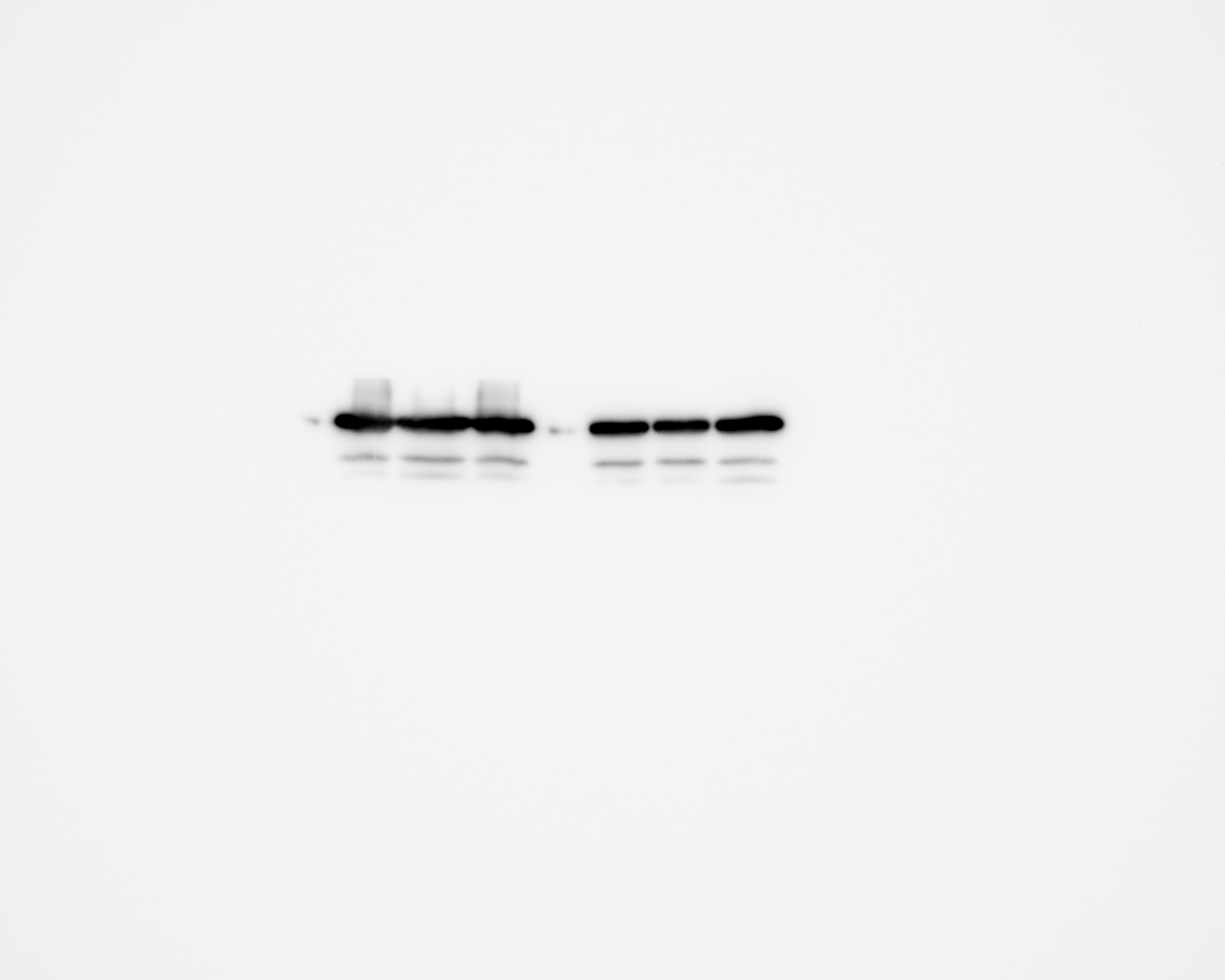

Supplement: Figure 2—figure supplement 1—source data 2. [file elife-107538-fig2-figsupp1-data2.zip › Figure 2- figure supplement 1F source data 2/GAPDH upper.tif]

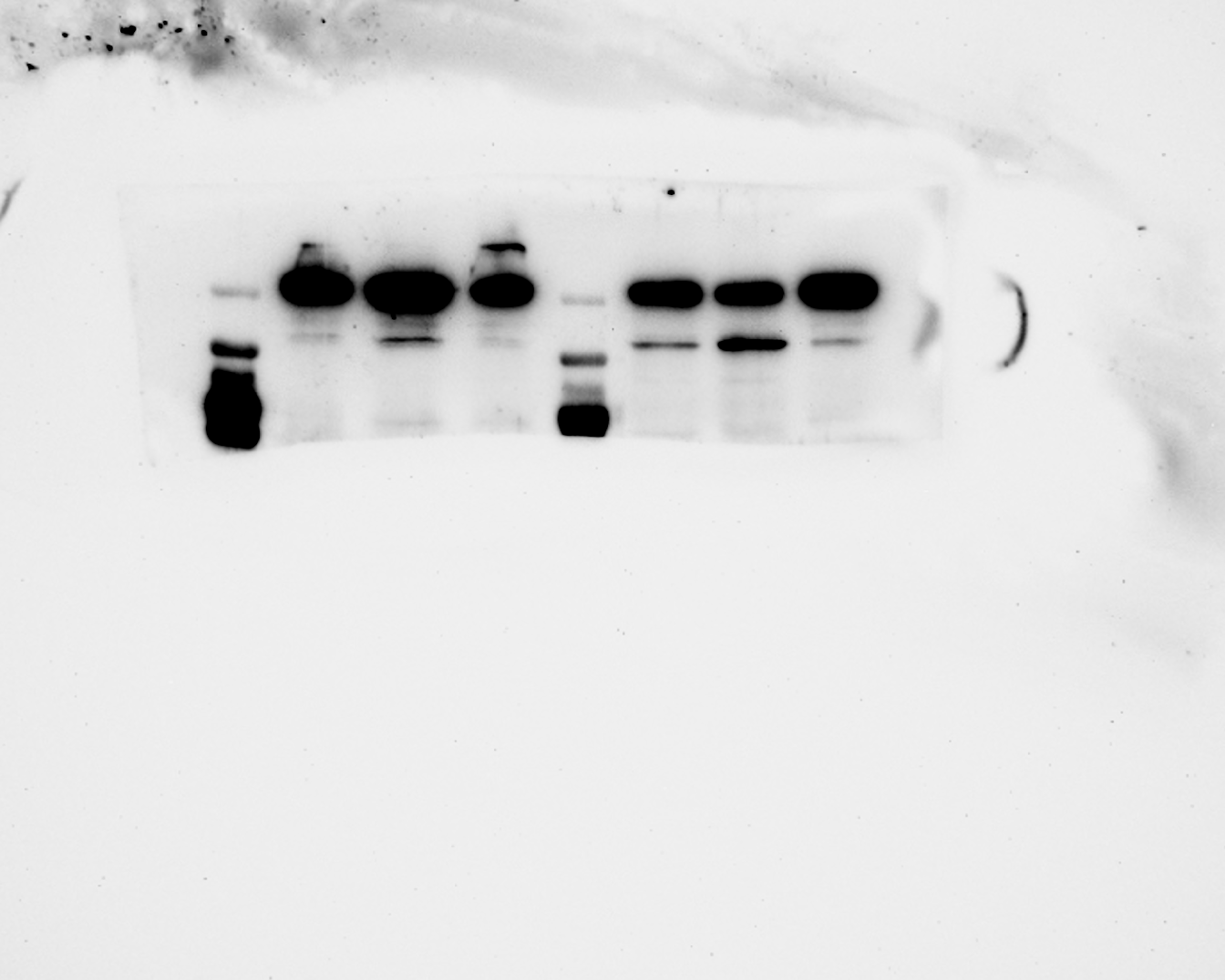

Supplement: Figure 2—figure supplement 1—source data 2. [file elife-107538-fig2-figsupp1-data2.zip › Figure 2- figure supplement 1F source data 2/HKDC1.tif]

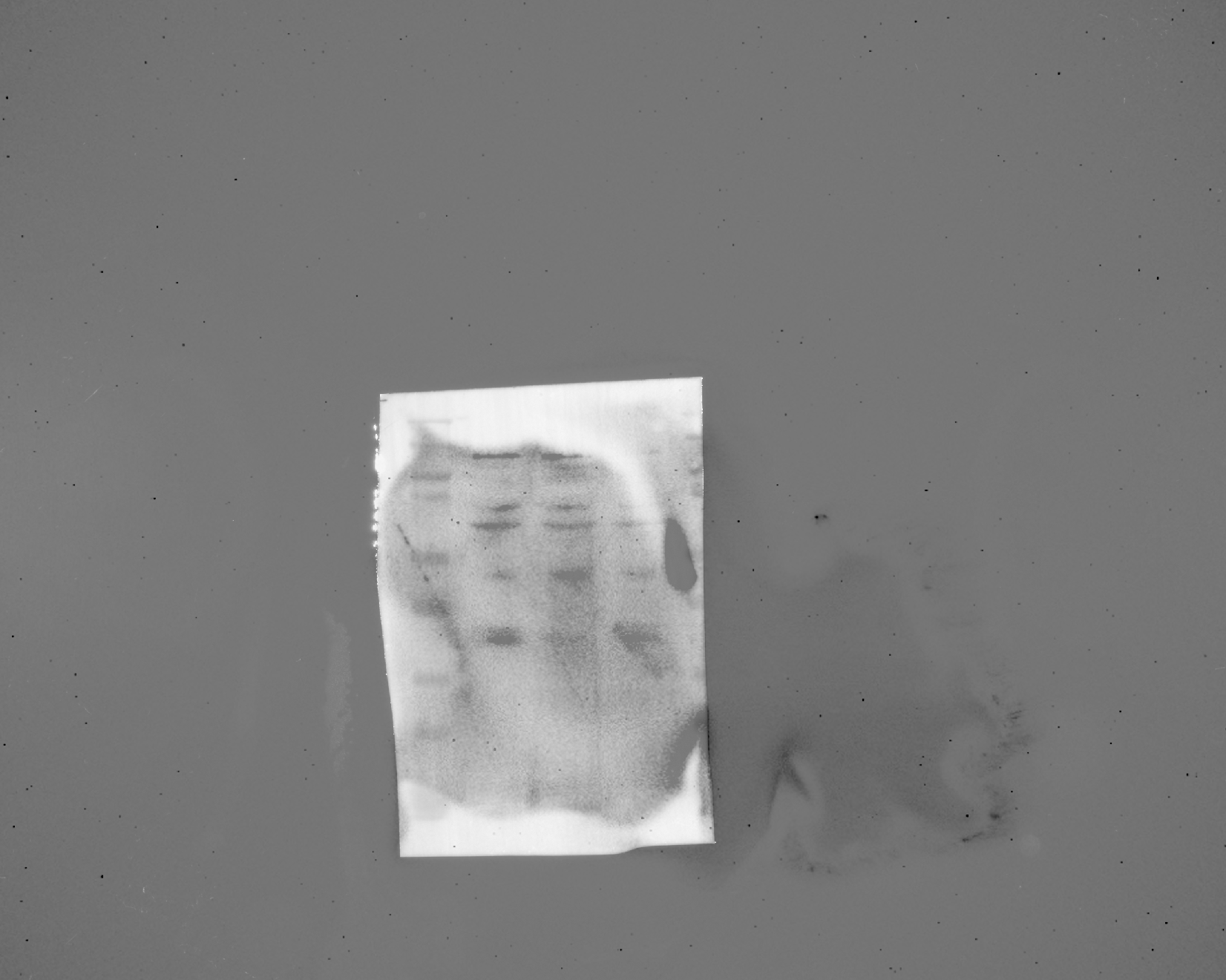

Supplement: Figure 2—figure supplement 1—source data 2. [file elife-107538-fig2-figsupp1-data2.zip › Figure 2- figure supplement 1F source data 2/ZMAT3.tif]

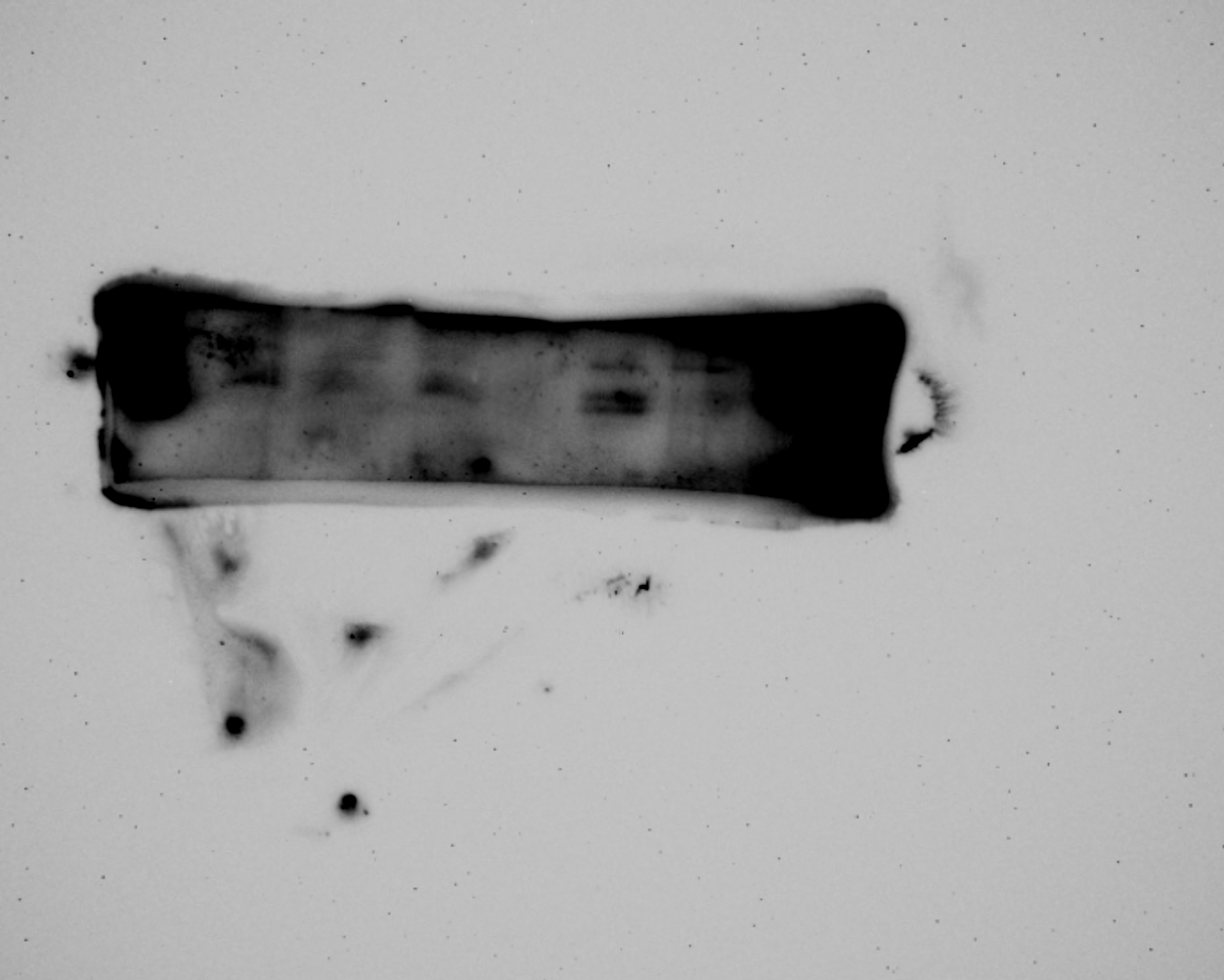

Supplement: Figure 2—figure supplement 1—source data 2. [file elife-107538-fig2-figsupp1-data2.zip › Figure 2- figure supplement 1G source data 2/ZMAT3.tif]

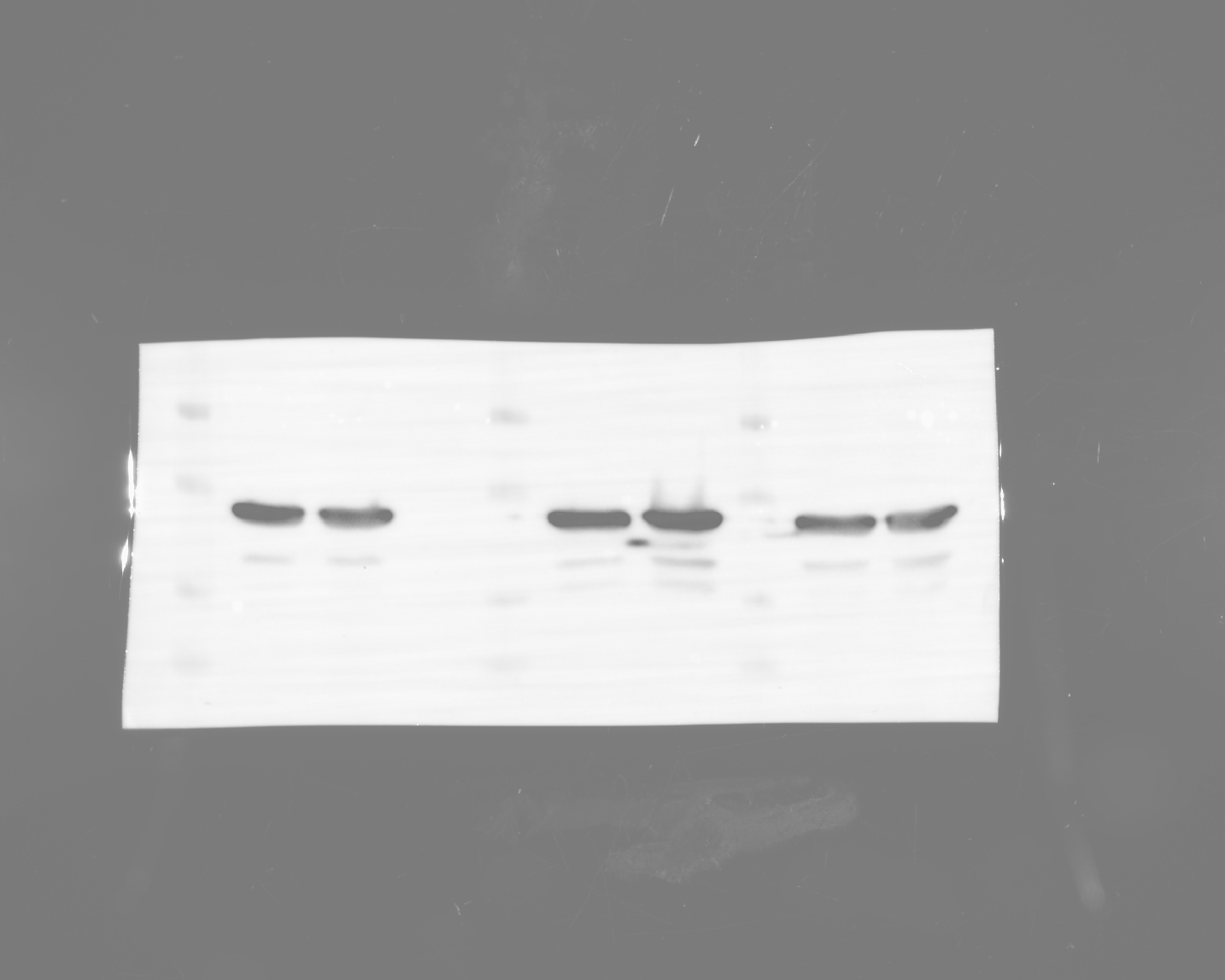

Supplement: Figure 4—source data 2. [file elife-107538-fig4-data2.zip › Figure 4D source data 2/GAPDH.tif]

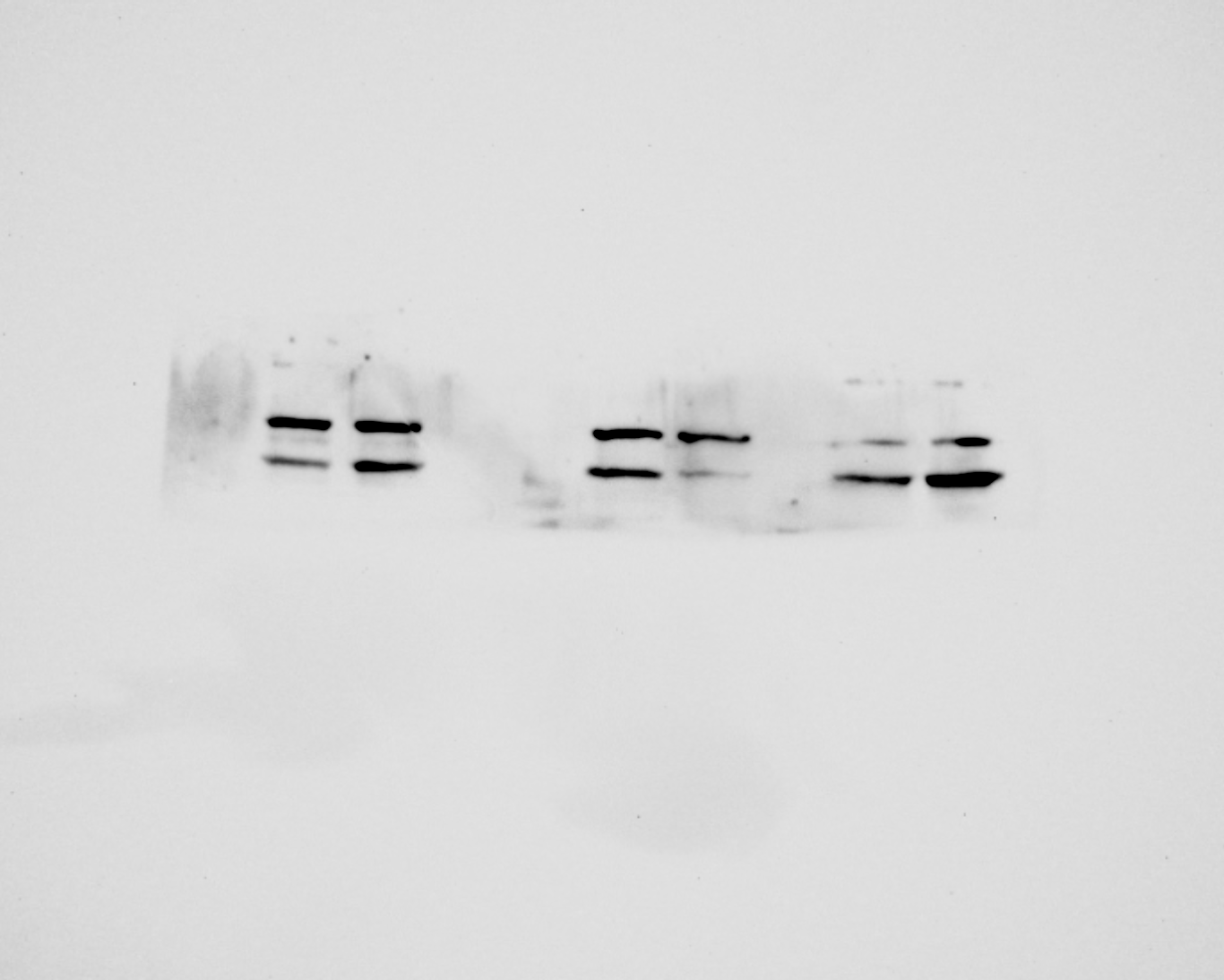

Supplement: Figure 4—source data 2. [file elife-107538-fig4-data2.zip › Figure 4D source data 2/HKDC1.tif]

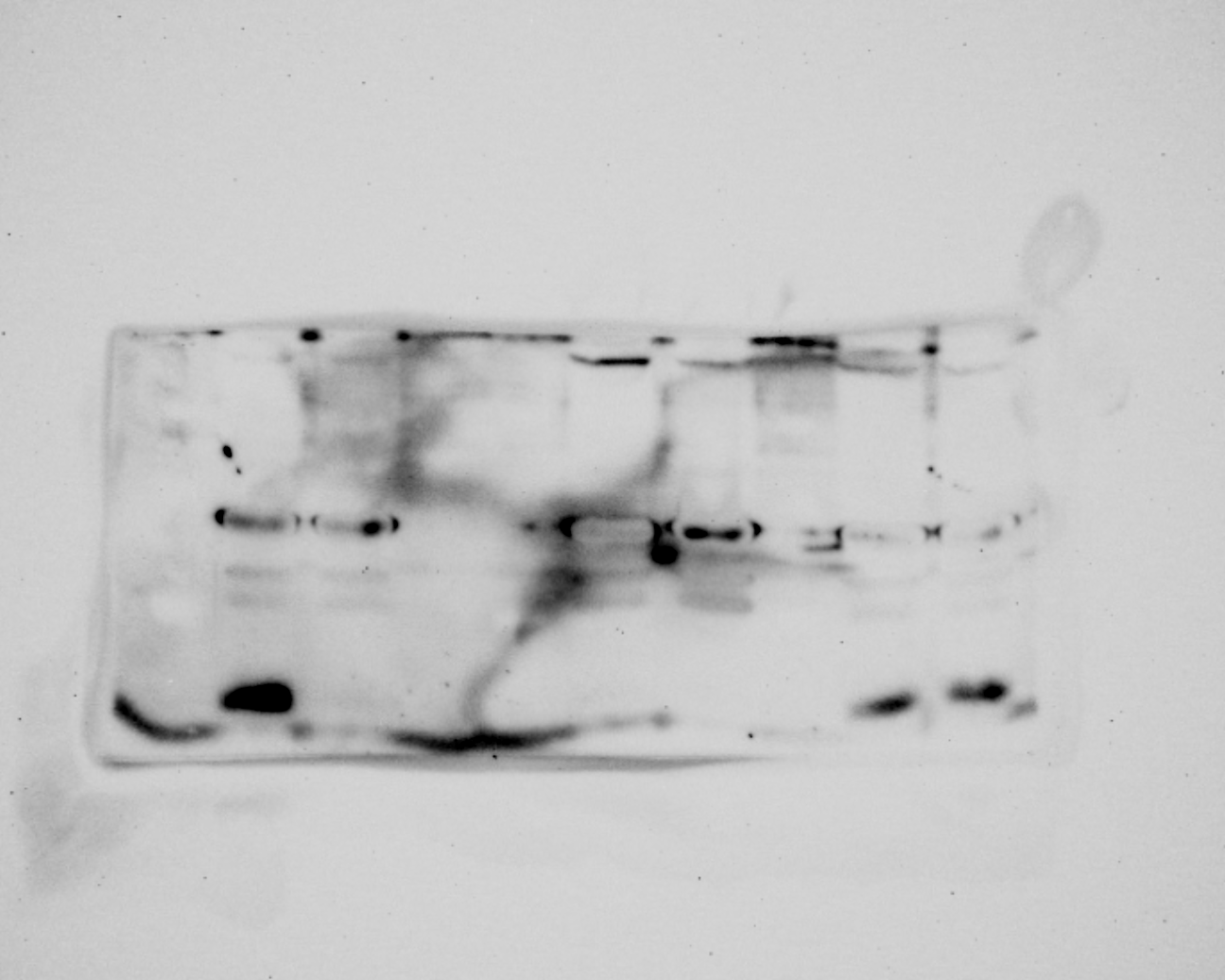

Supplement: Figure 4—source data 2. [file elife-107538-fig4-data2.zip › Figure 4D source data 2/p21.tif]

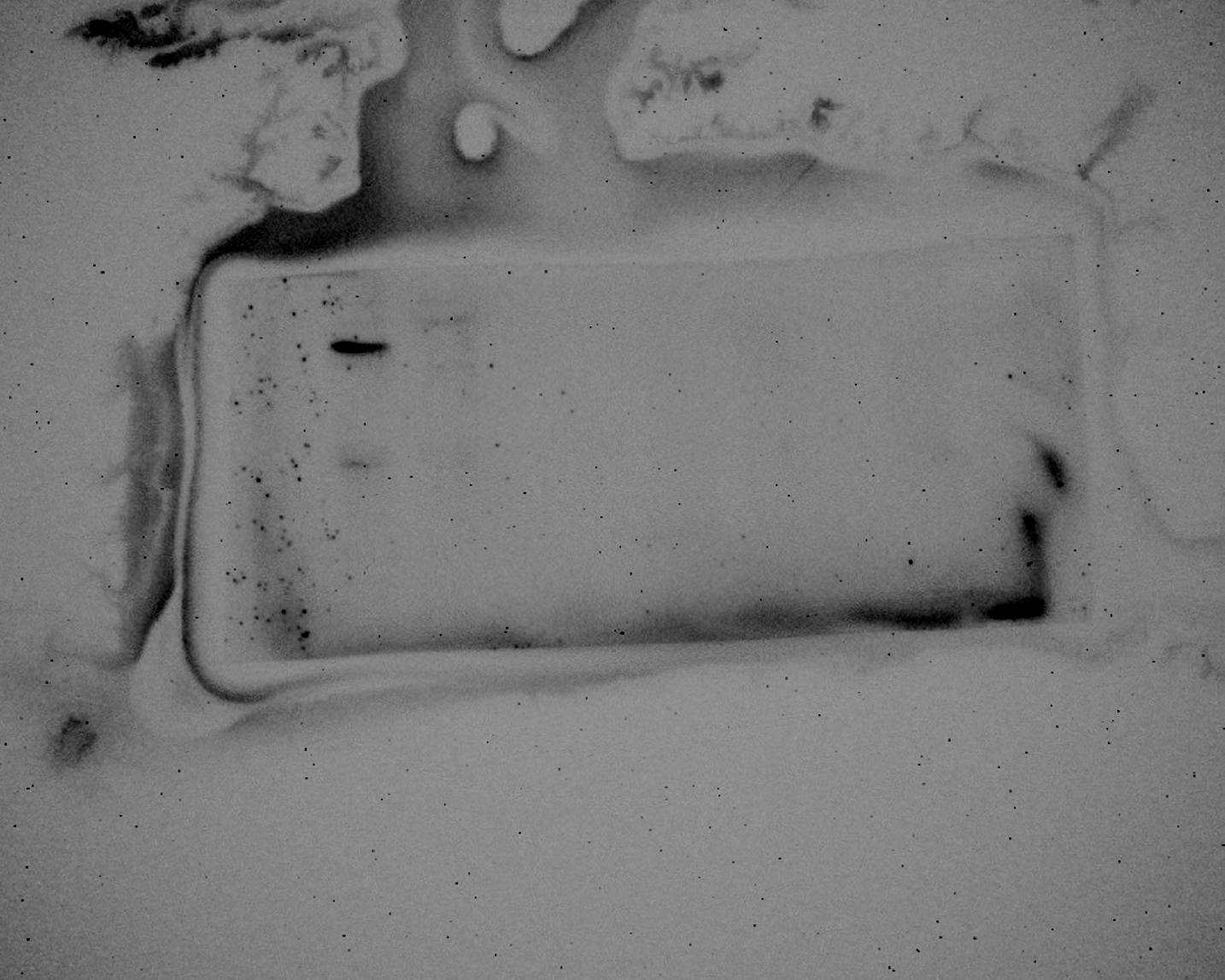

Supplement: Figure 4—source data 2. [file elife-107538-fig4-data2.zip › Figure 4D source data 2/p53.tif]

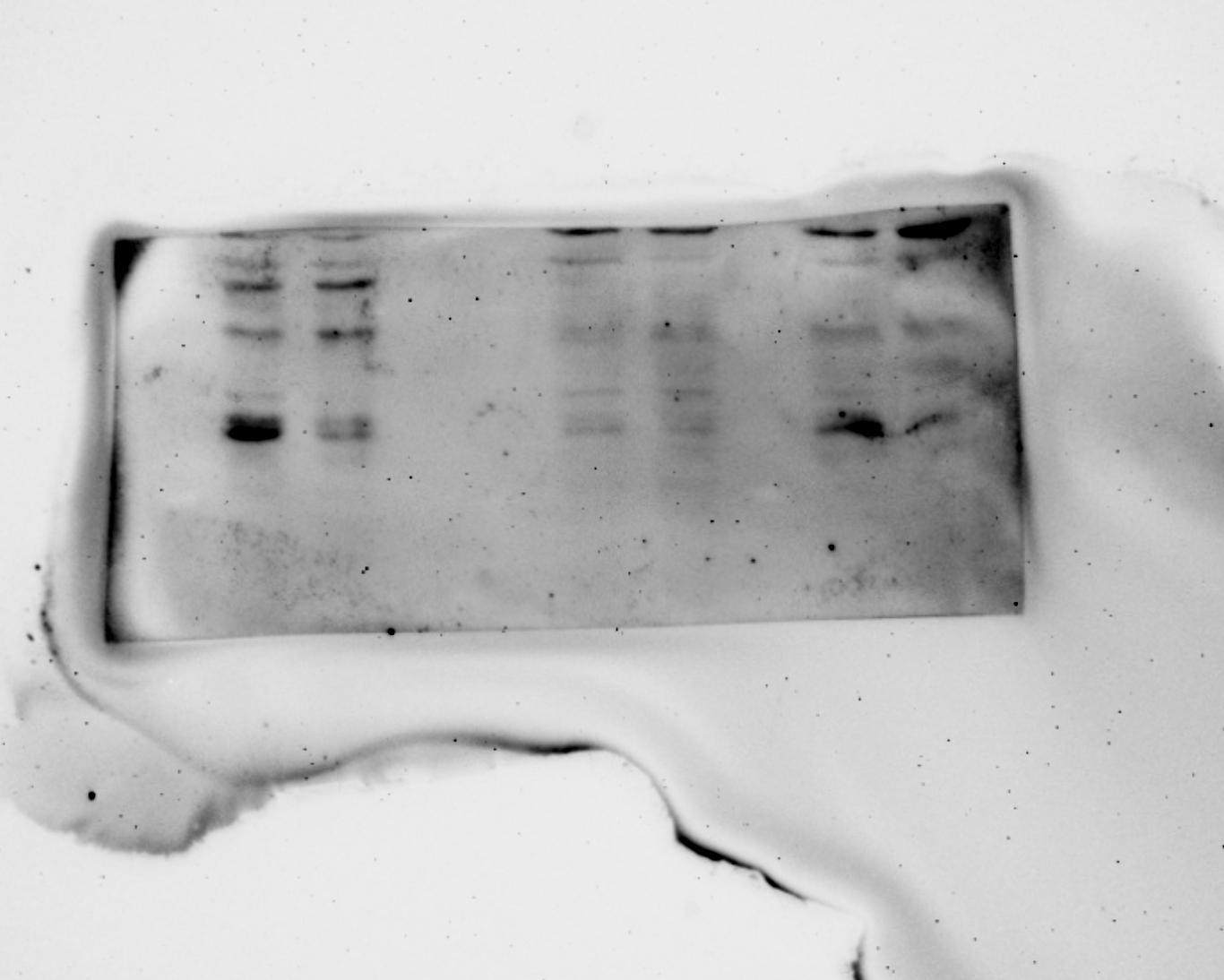

Supplement: Figure 4—source data 2. [file elife-107538-fig4-data2.zip › Figure 4D source data 2/ZMAT3.tif]

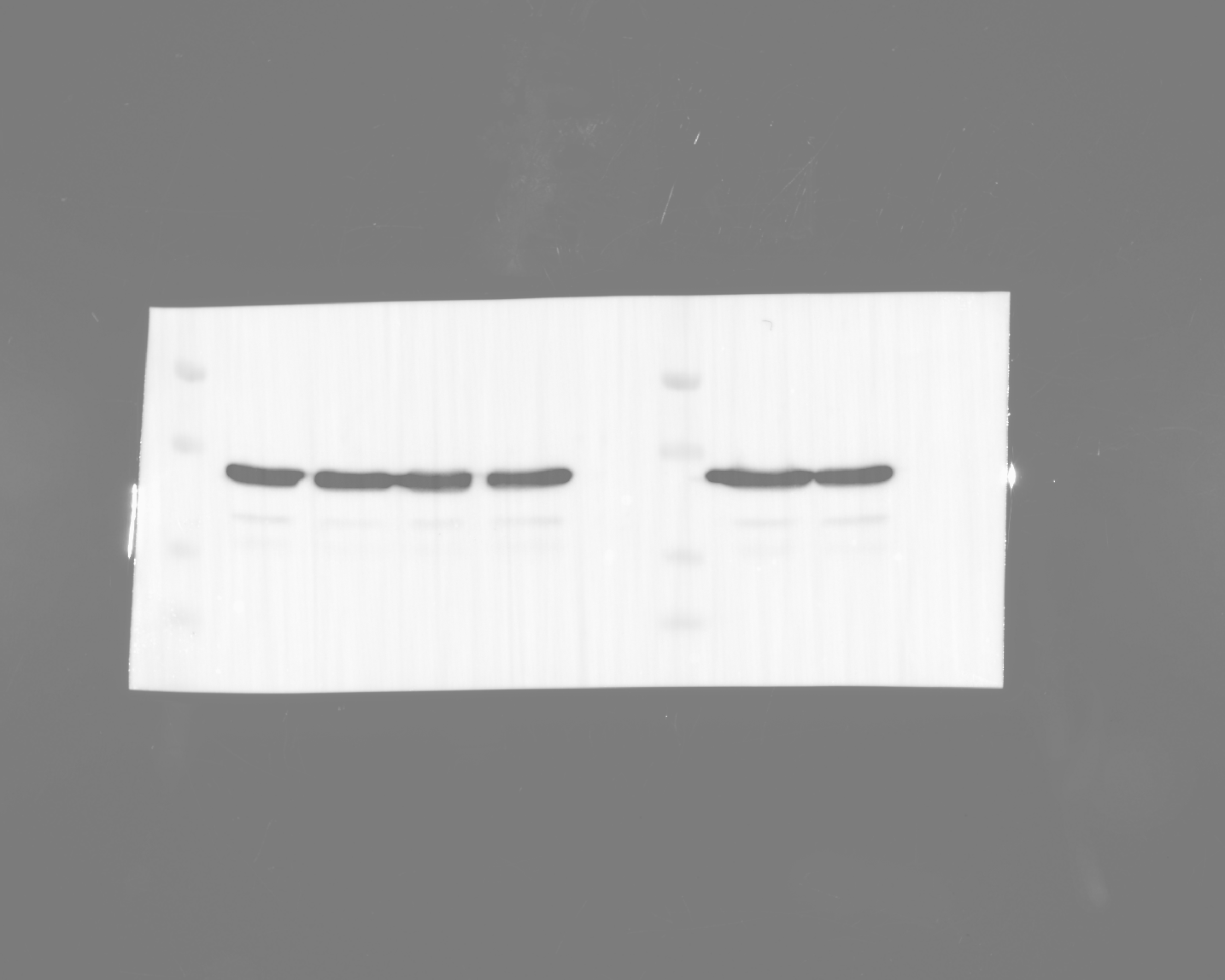

Supplement: Figure 4—source data 2. [file elife-107538-fig4-data2.zip › Figure 4F source data 2/GAPDH.tif]

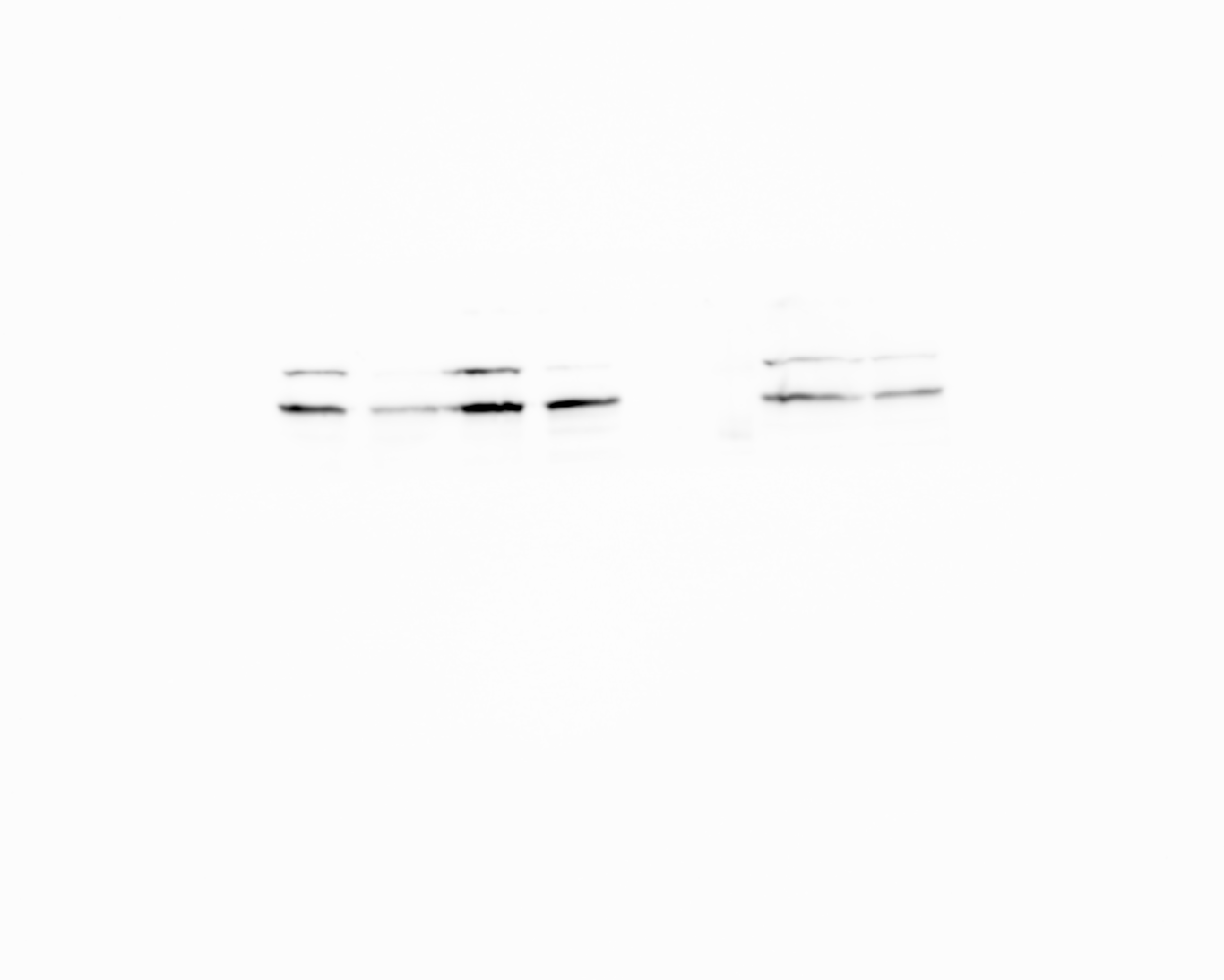

Supplement: Figure 4—source data 2. [file elife-107538-fig4-data2.zip › Figure 4F source data 2/HKDC1.tif]

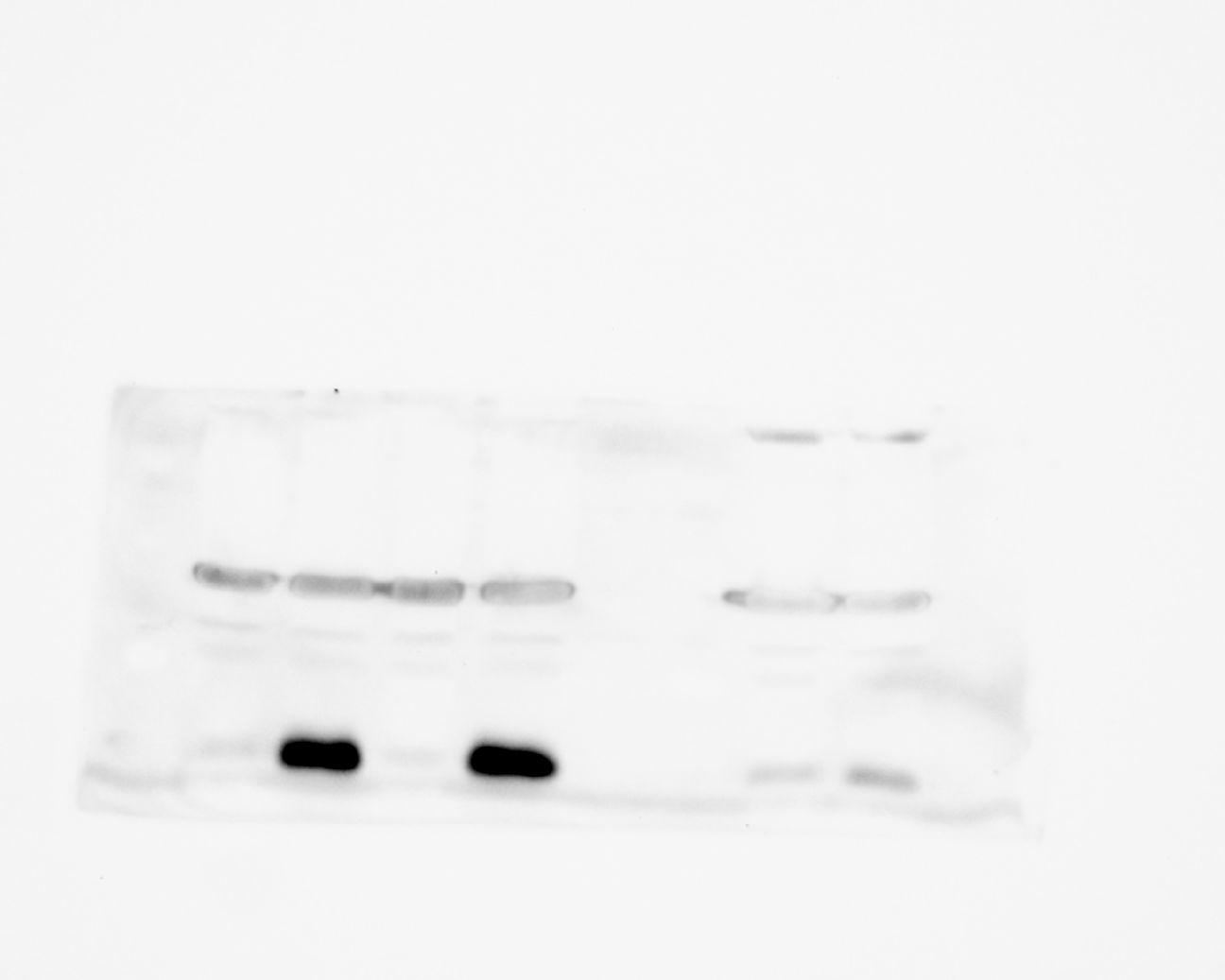

Supplement: Figure 4—source data 2. [file elife-107538-fig4-data2.zip › Figure 4F source data 2/p21.tif]

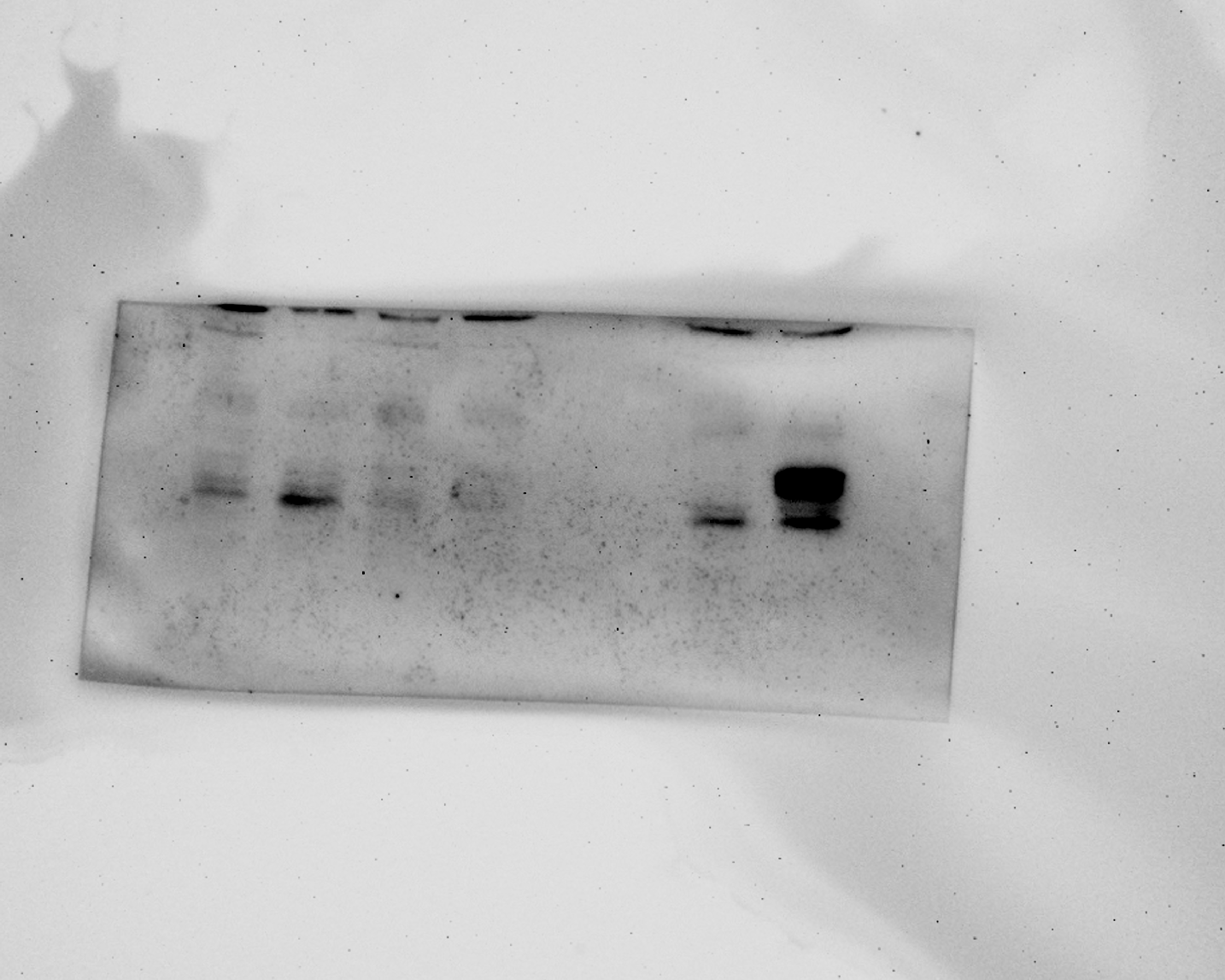

Supplement: Figure 4—source data 2. [file elife-107538-fig4-data2.zip › Figure 4F source data 2/ZMAT3.tif]

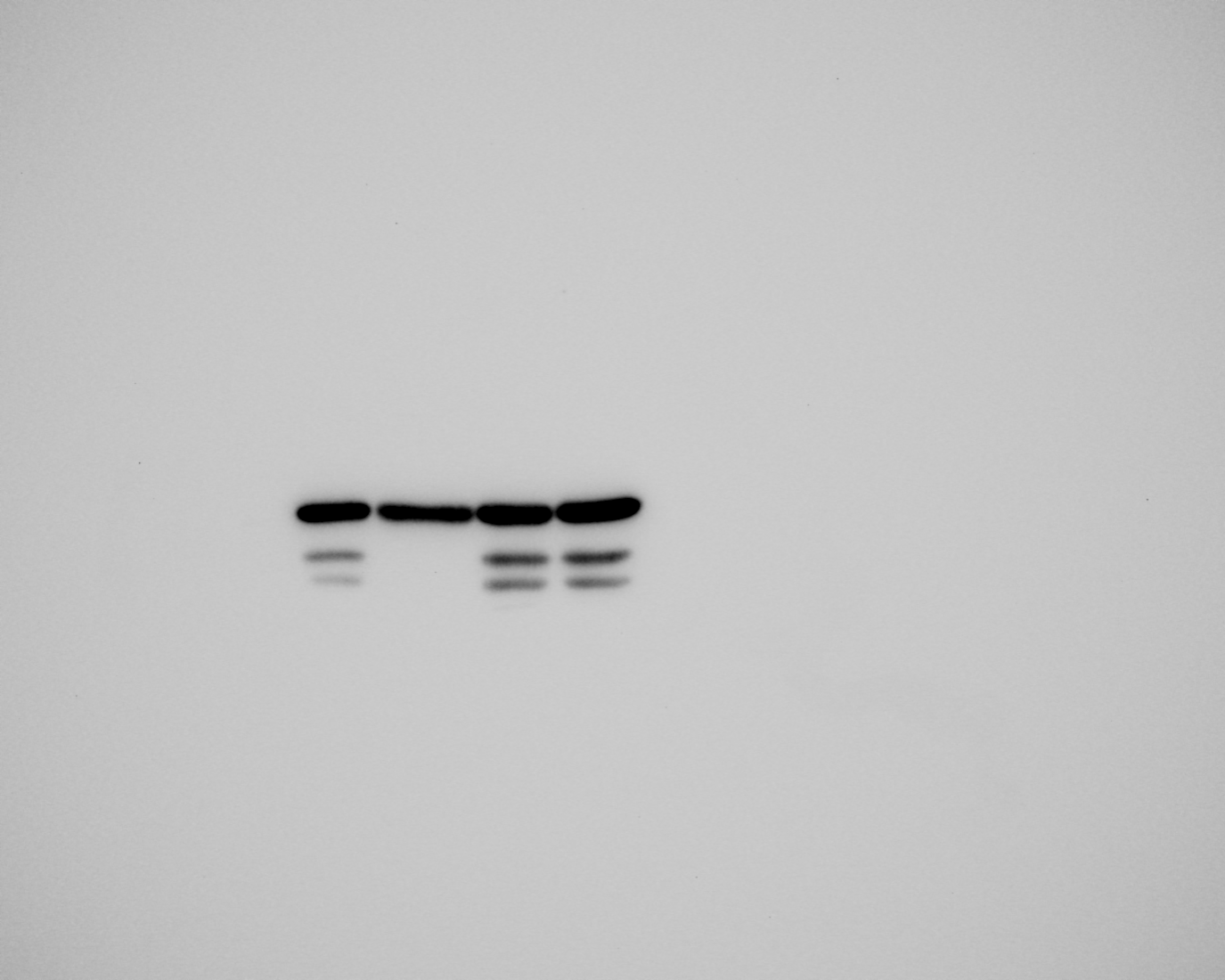

Supplement: Figure 4—source data 2. [file elife-107538-fig4-data2.zip › Figure 4J source data 2/GAPDH upper.tif]

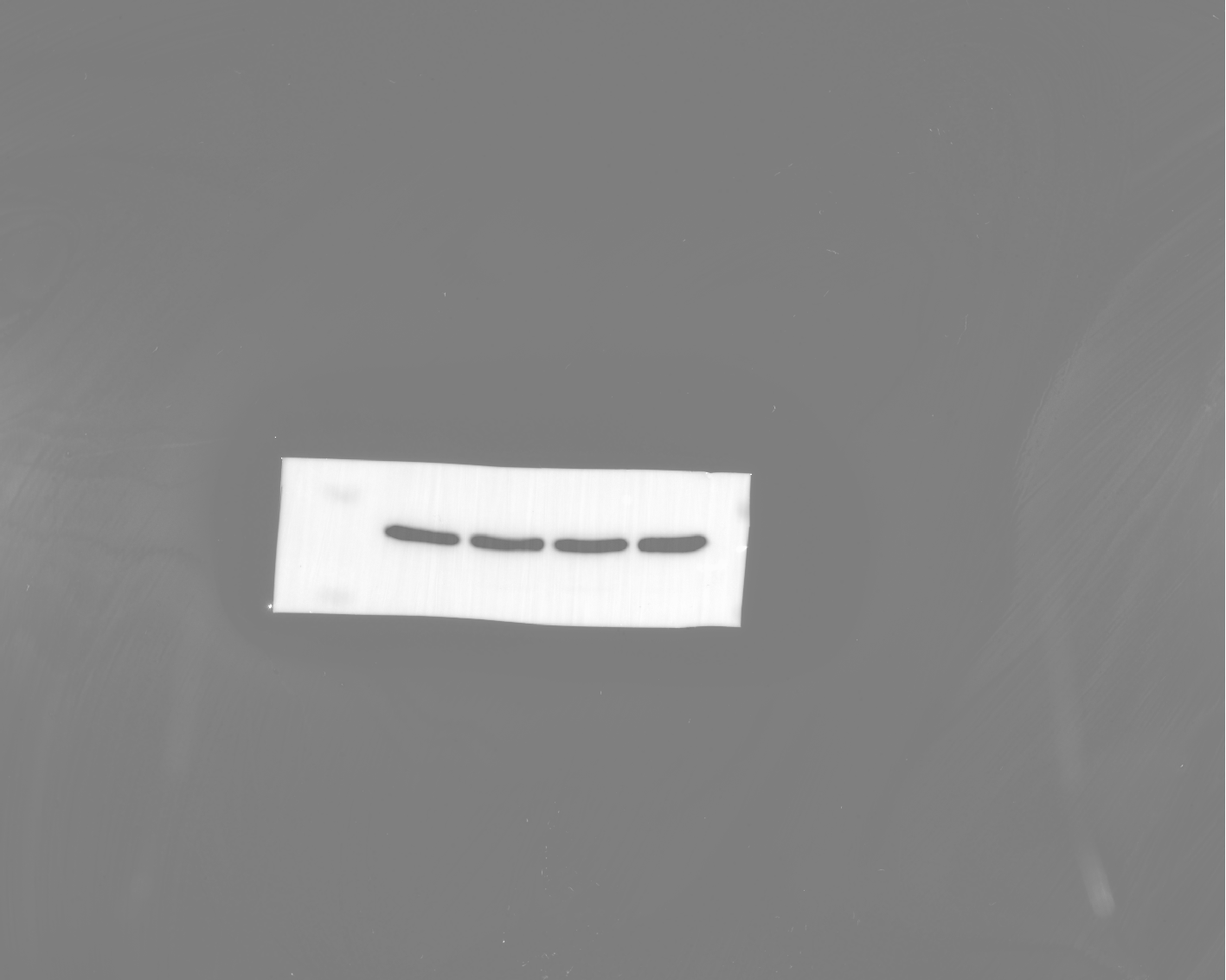

Supplement: Figure 4—source data 2. [file elife-107538-fig4-data2.zip › Figure 4J source data 2/GAPDH.tif]

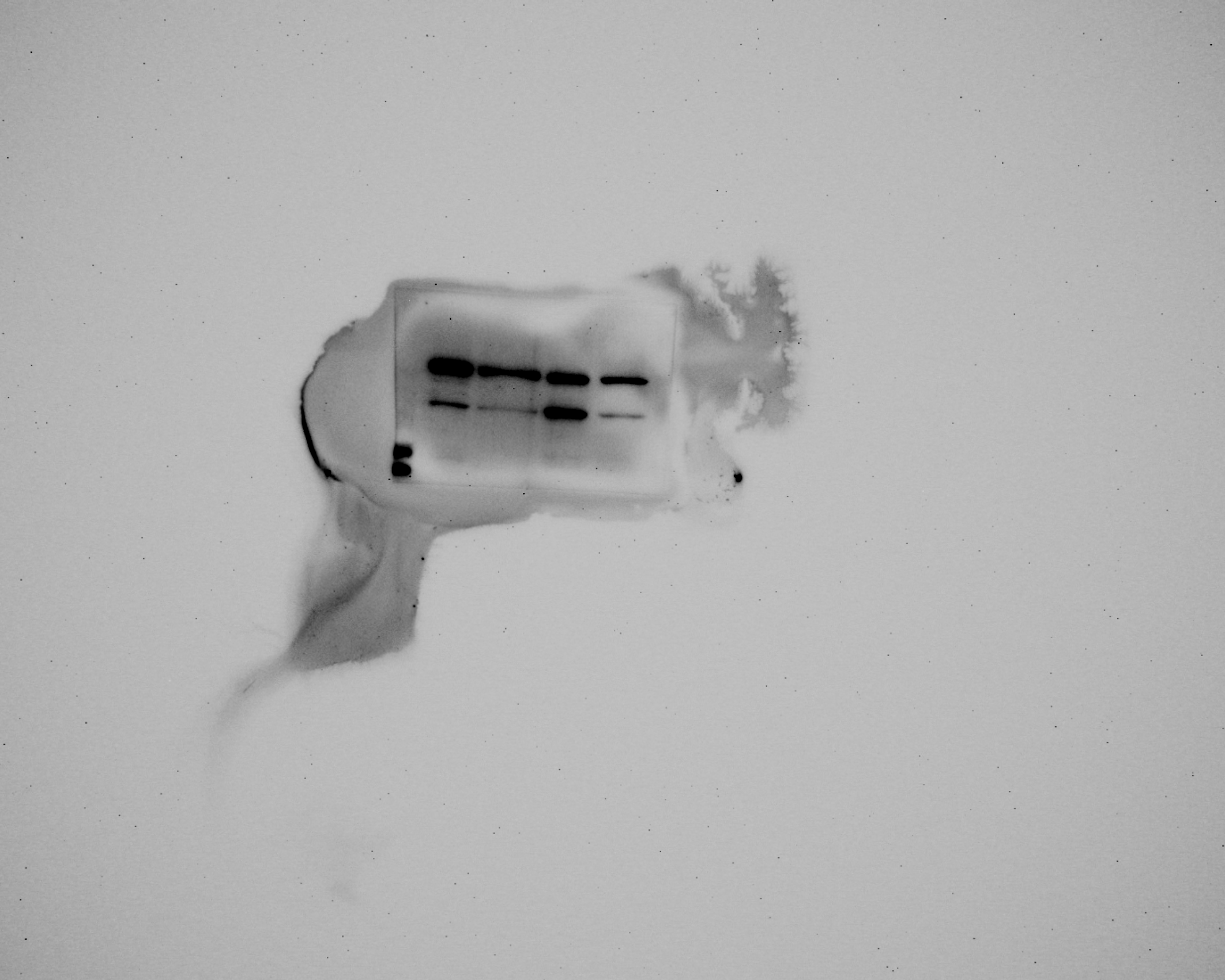

Supplement: Figure 4—source data 2. [file elife-107538-fig4-data2.zip › Figure 4J source data 2/HKDC1.tif]

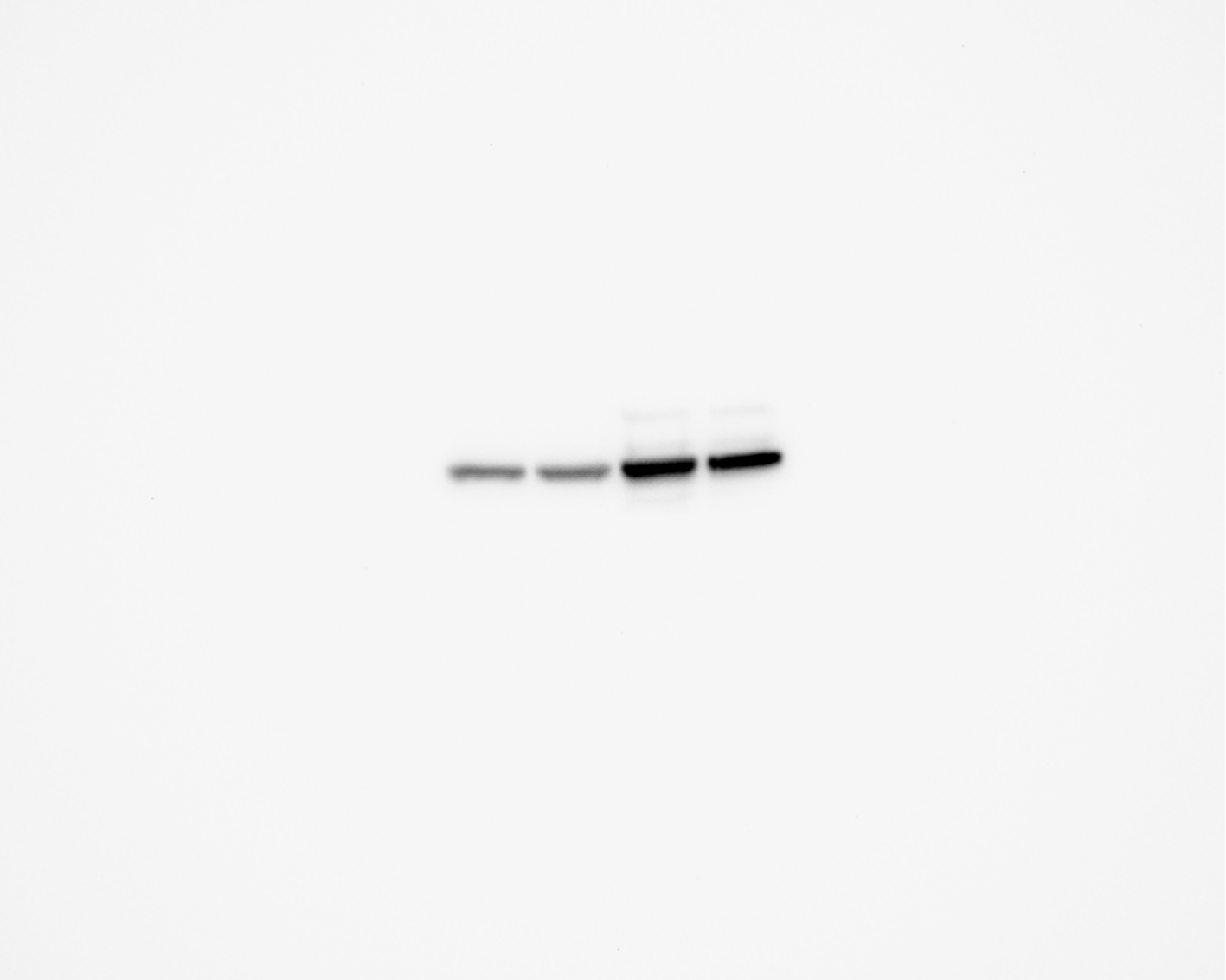

Supplement: Figure 4—source data 2. [file elife-107538-fig4-data2.zip › Figure 4J source data 2/p53.tif]

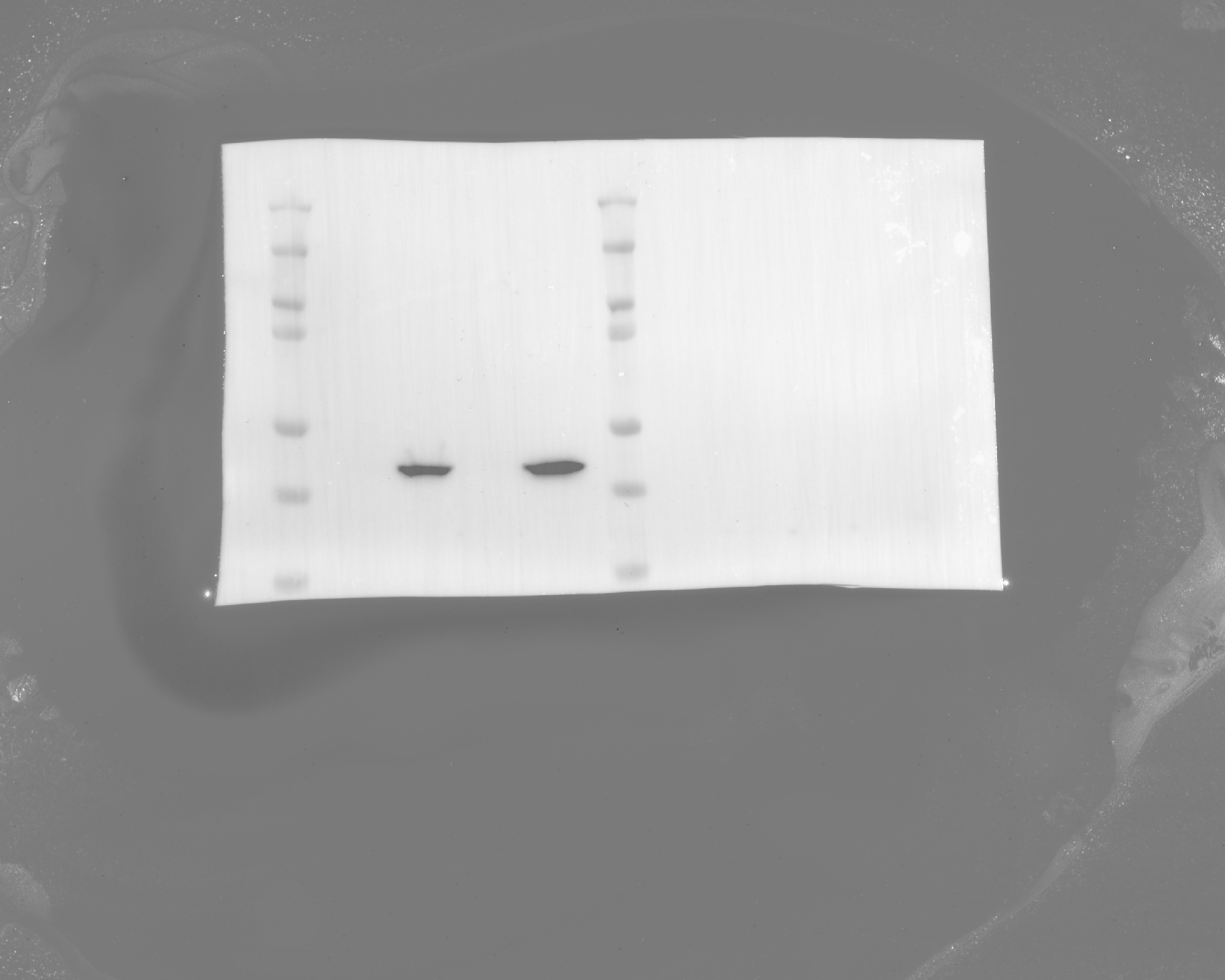

Supplement: Figure 4—source data 2. [file elife-107538-fig4-data2.zip › Figure 4J source data 2/ZMAT3.tif]

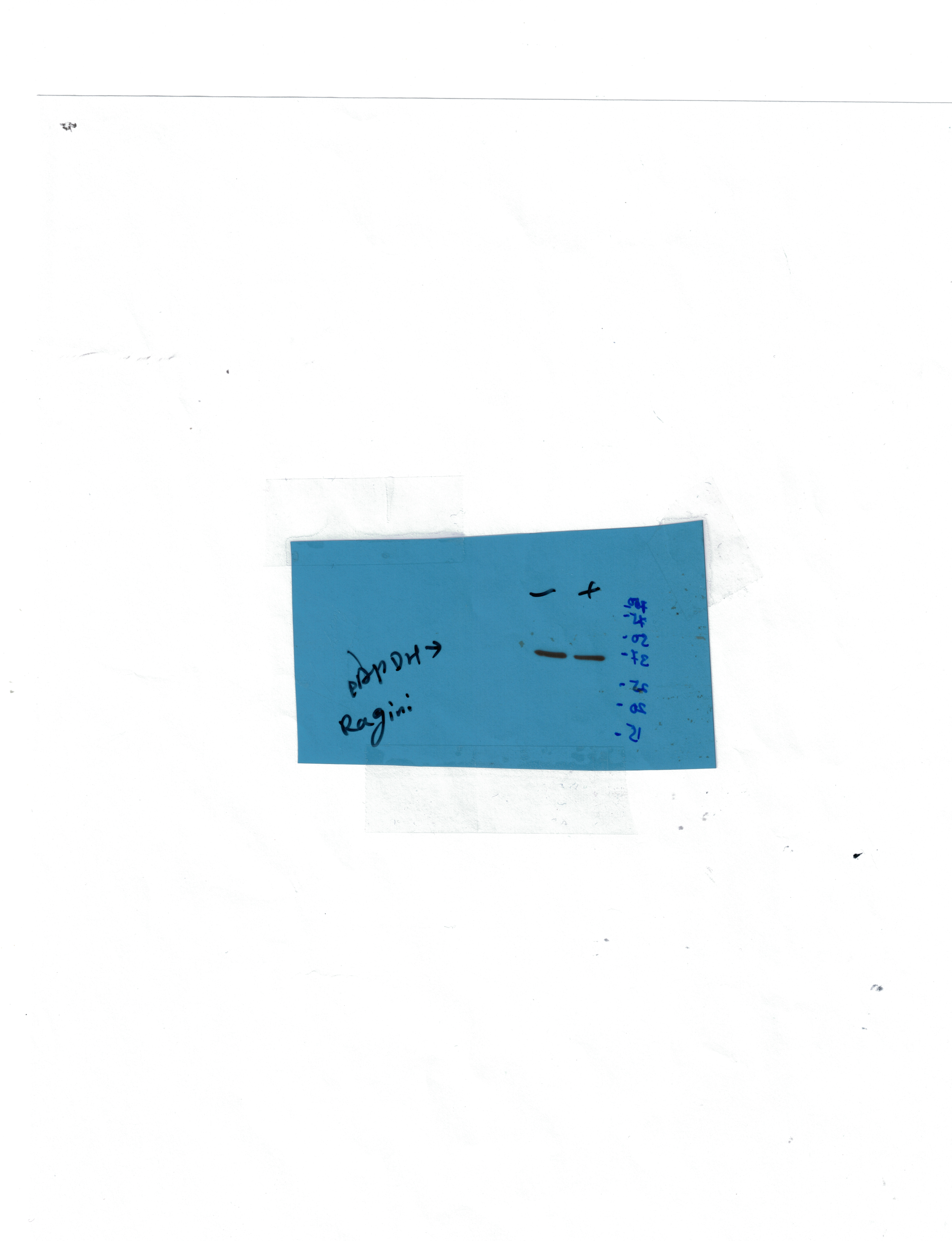

Supplement: Figure 4—source data 2. [file elife-107538-fig4-data2.zip › Figure 4H source data 2/GAPDH.tif]

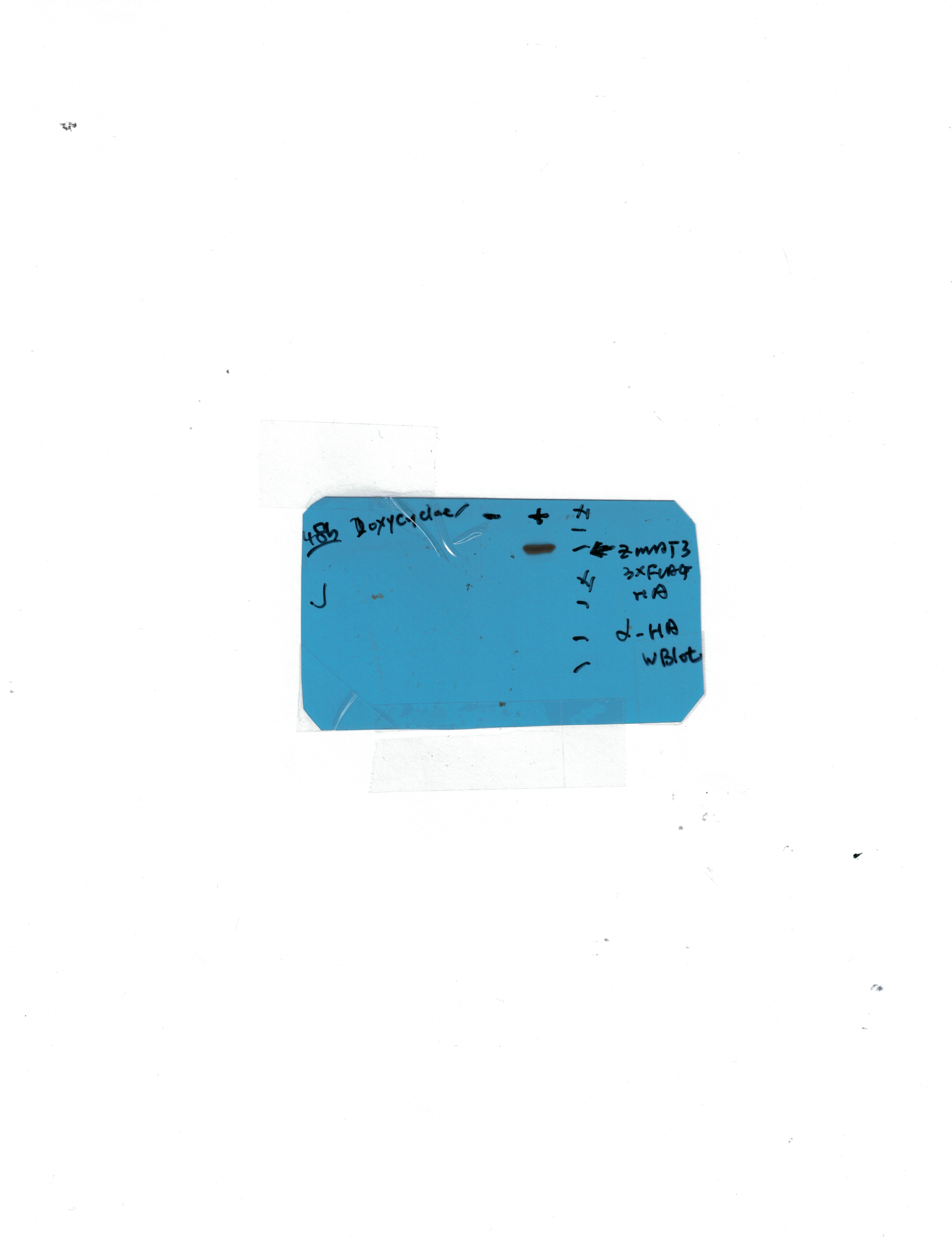

Supplement: Figure 4—source data 2. [file elife-107538-fig4-data2.zip › Figure 4H source data 2/ZMAT3 FLAG HA.tif]

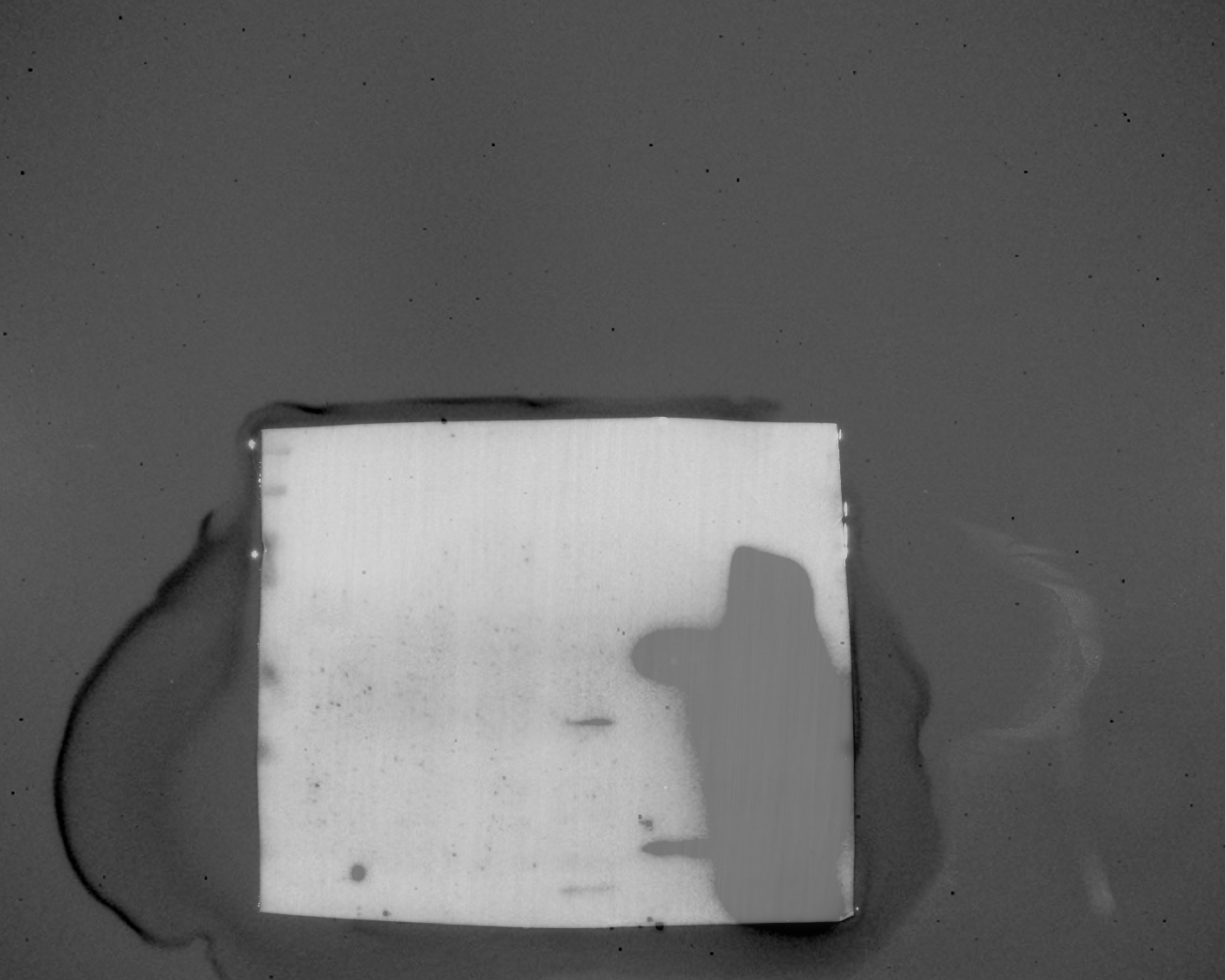

Supplement: Figure 5—source data 2. [file elife-107538-fig5-data2.zip › Figure 5D source data 2/FLAG high exposure.tif]

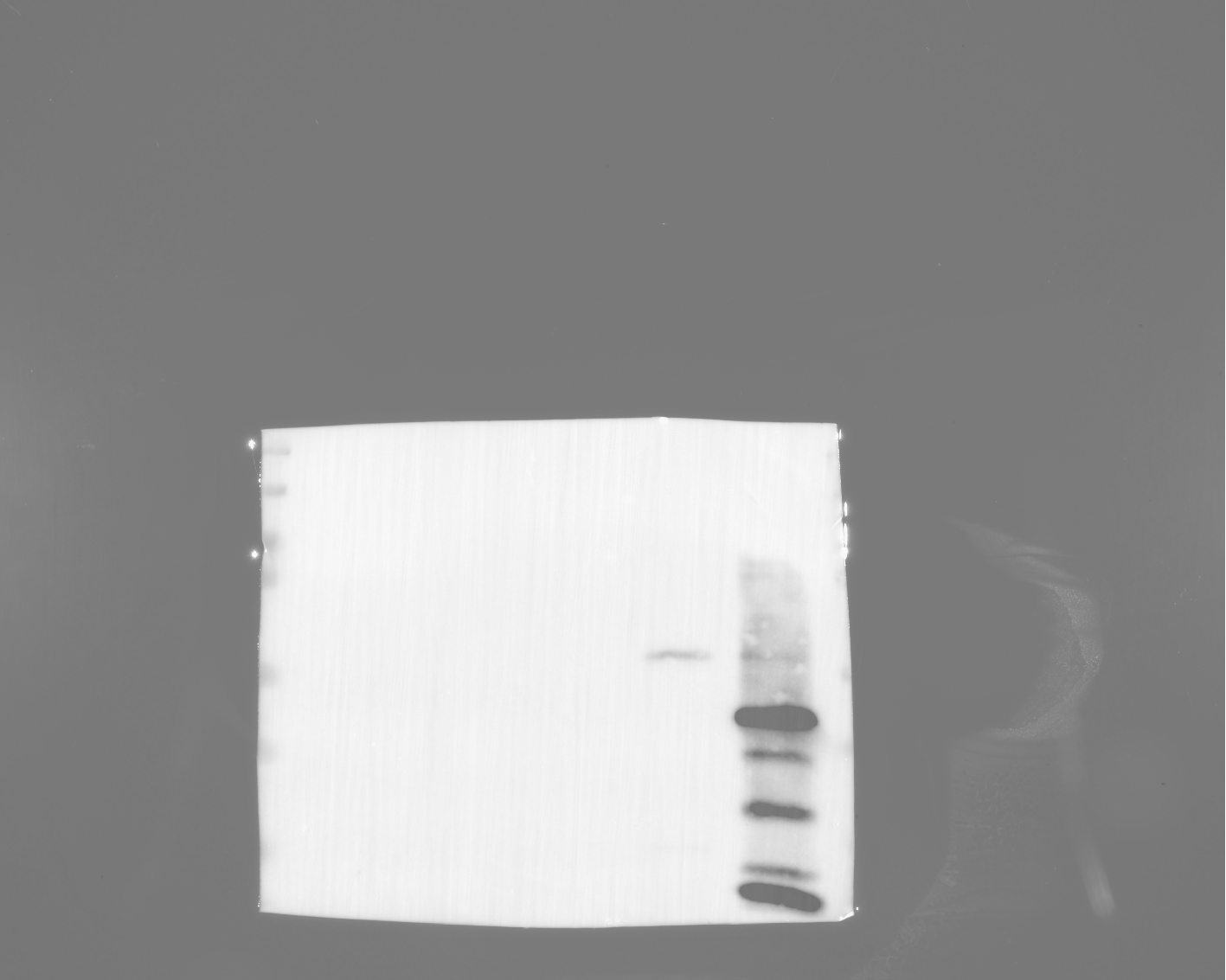

Supplement: Figure 5—source data 2. [file elife-107538-fig5-data2.zip › Figure 5D source data 2/FLAG low exposure.tif]

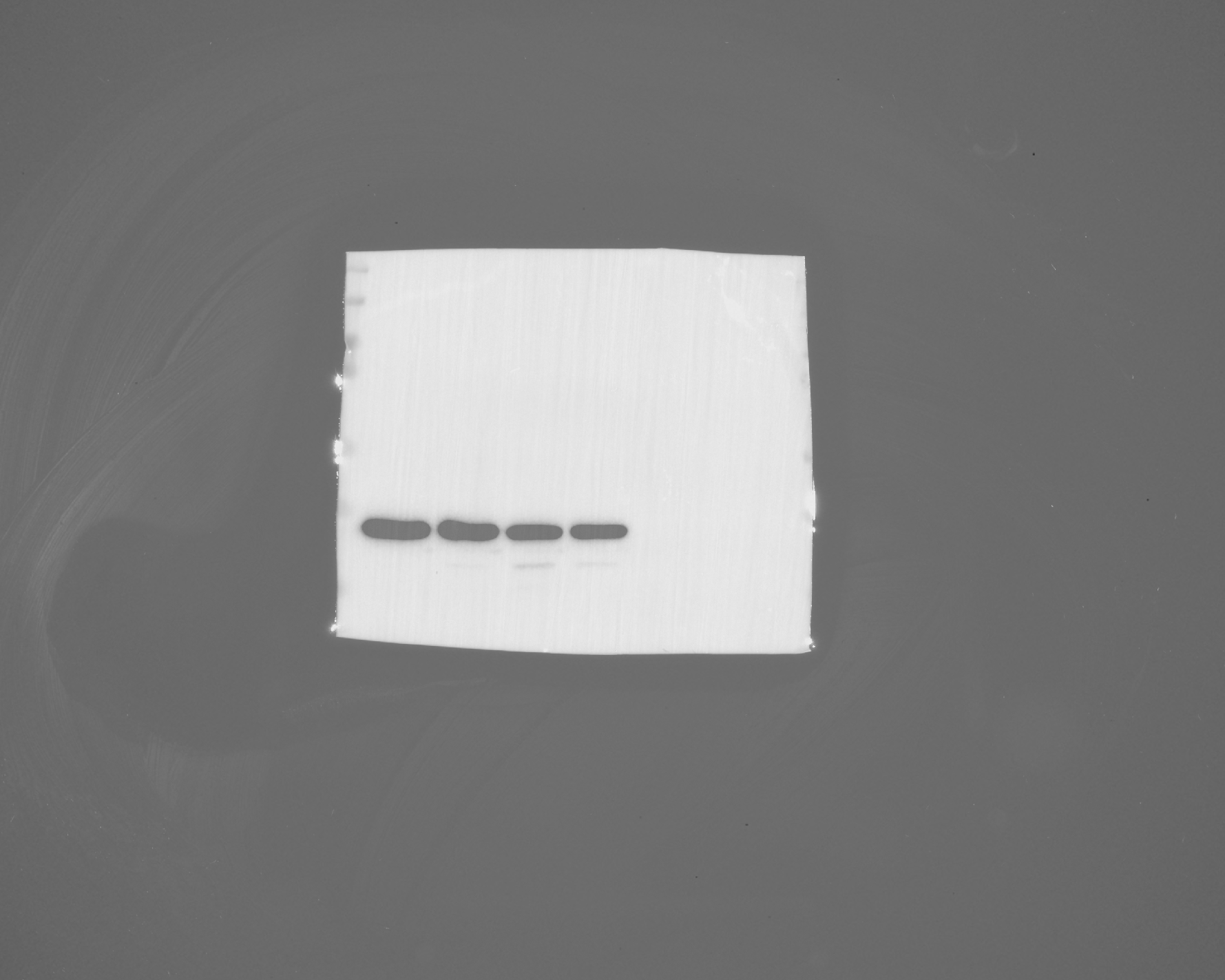

Supplement: Figure 5—source data 2. [file elife-107538-fig5-data2.zip › Figure 5D source data 2/GAPDH.tif]

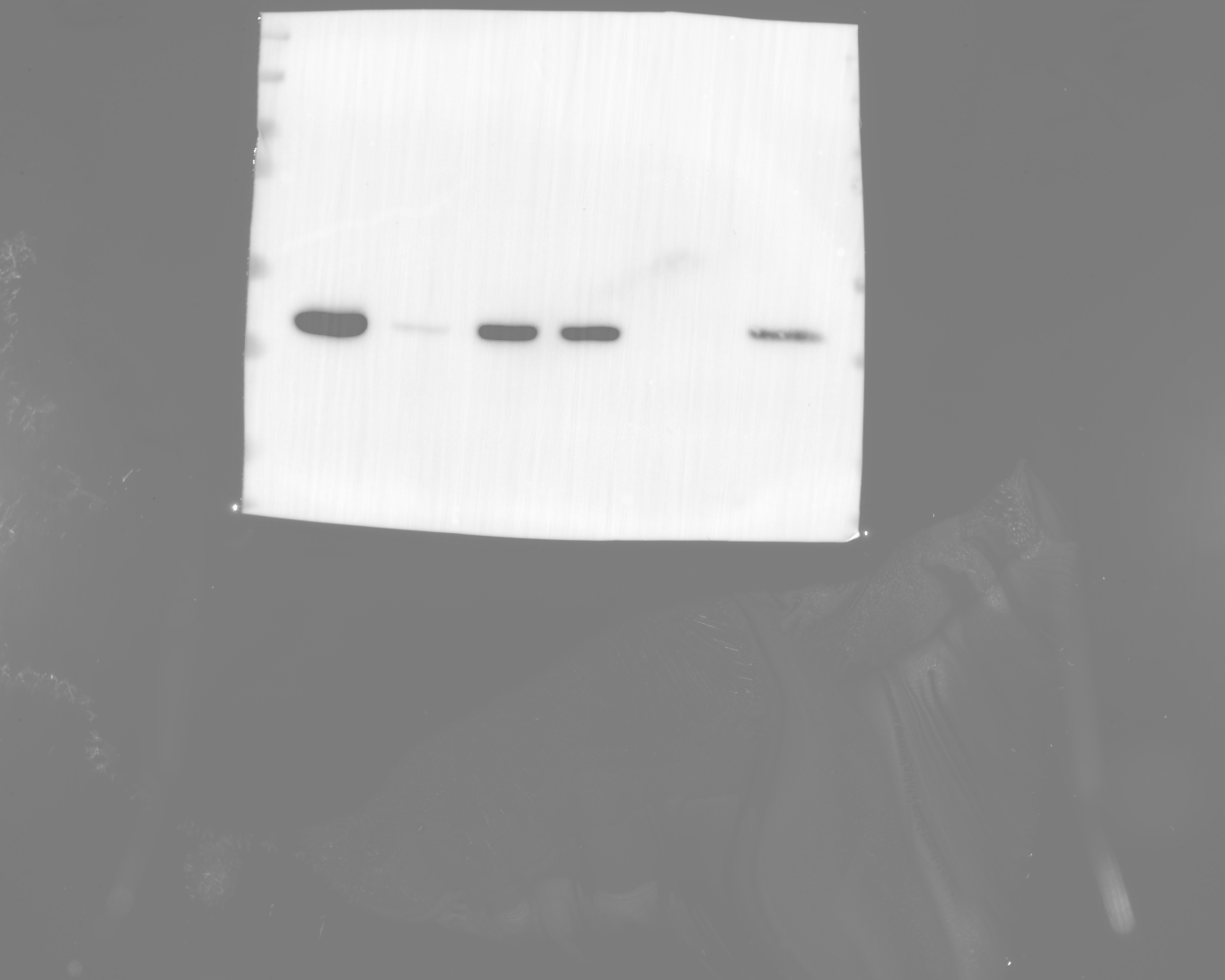

Supplement: Figure 5—source data 2. [file elife-107538-fig5-data2.zip › Figure 5D source data 2/JUN.tif]

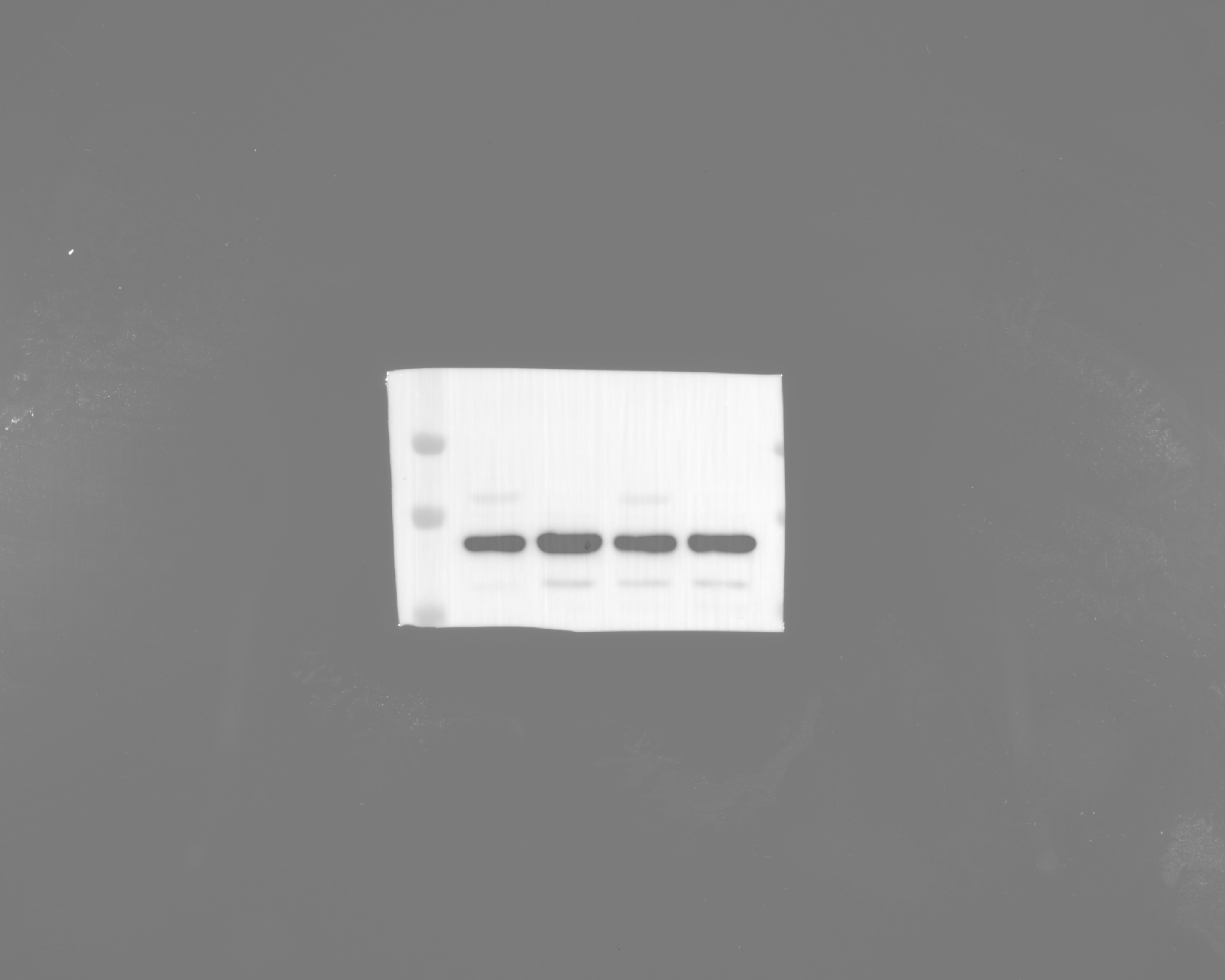

Supplement: Figure 5—source data 2. [file elife-107538-fig5-data2.zip › Figure 5F source data 2/GAPDH.tif]

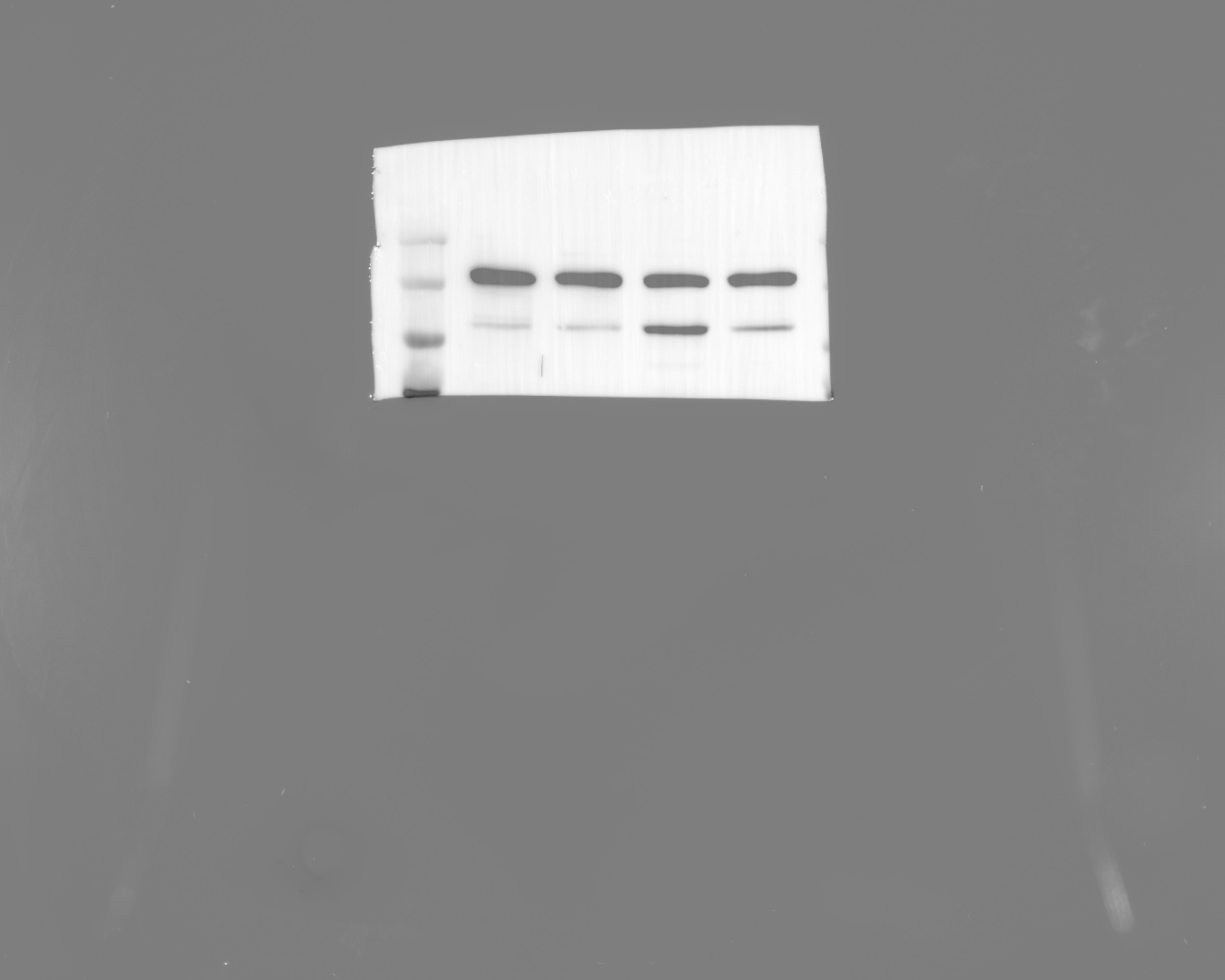

Supplement: Figure 5—source data 2. [file elife-107538-fig5-data2.zip › Figure 5F source data 2/HKDC1.tif]

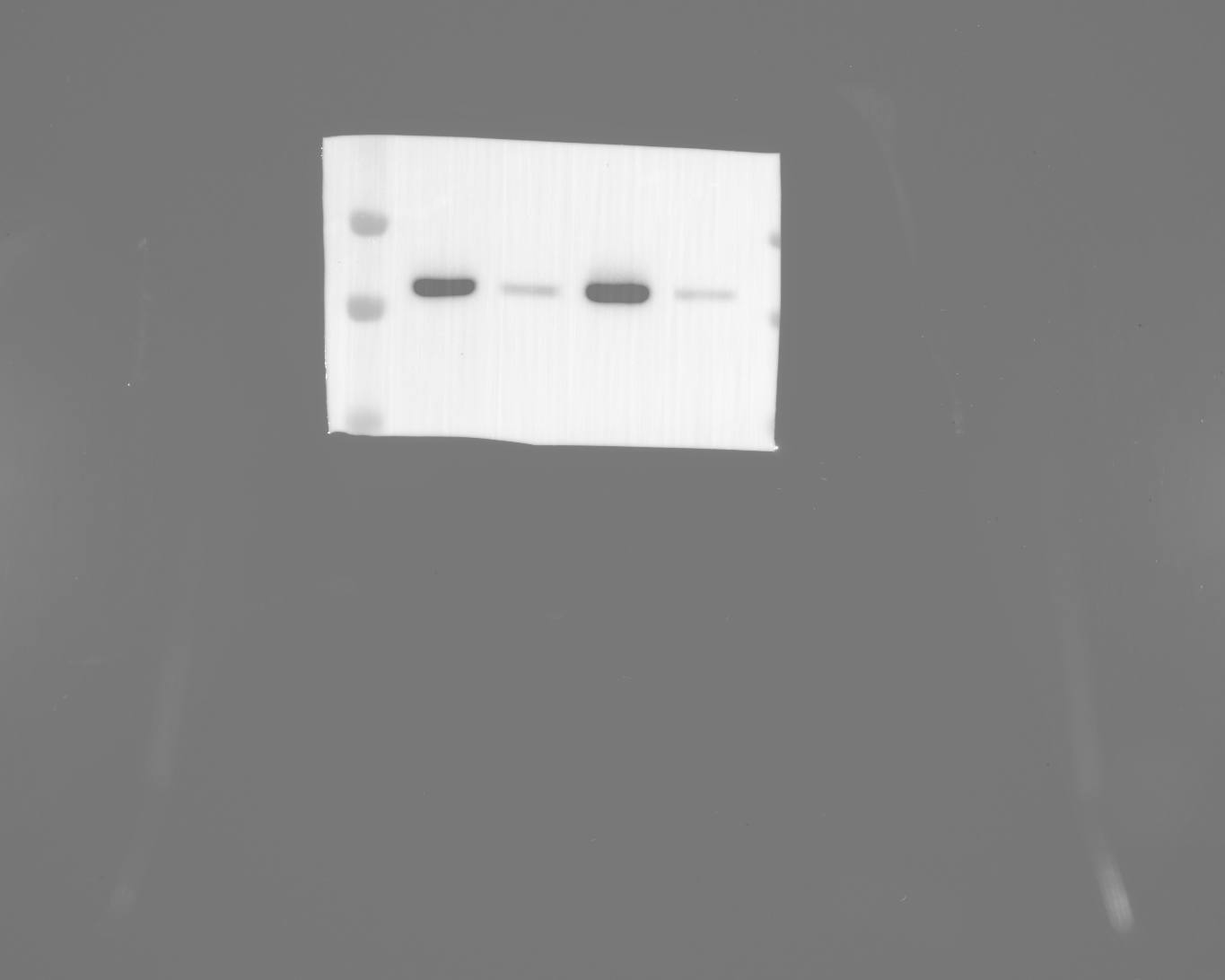

Supplement: Figure 5—source data 2. [file elife-107538-fig5-data2.zip › Figure 5F source data 2/JUN.tif]

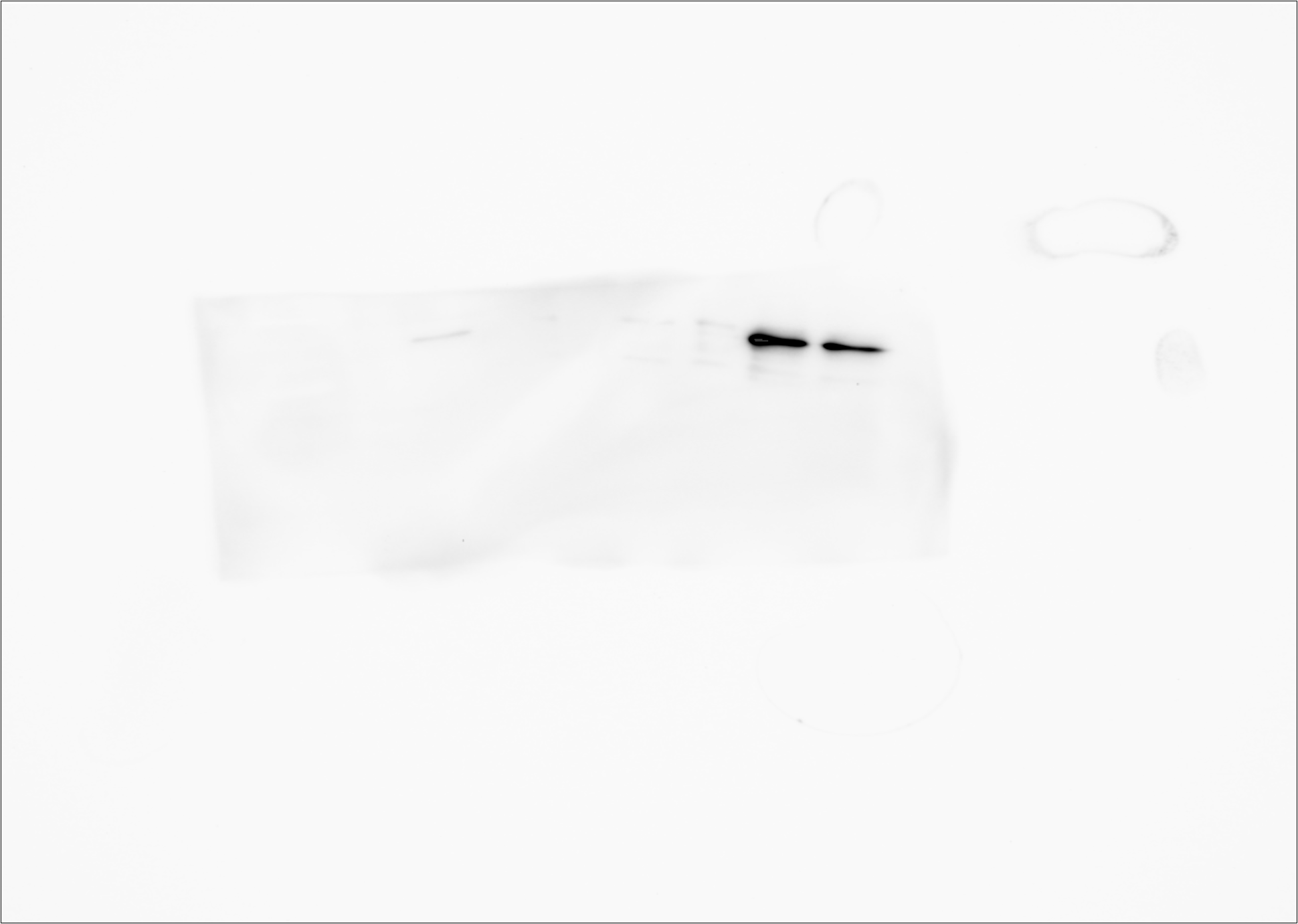

Supplement: Figure 5—figure supplement 1—source data 2. [file elife-107538-fig5-figsupp1-data2.zip › Figure 5- figure supplement 1C source data 2/ZMAT3-FLAG-HA 2.tif]

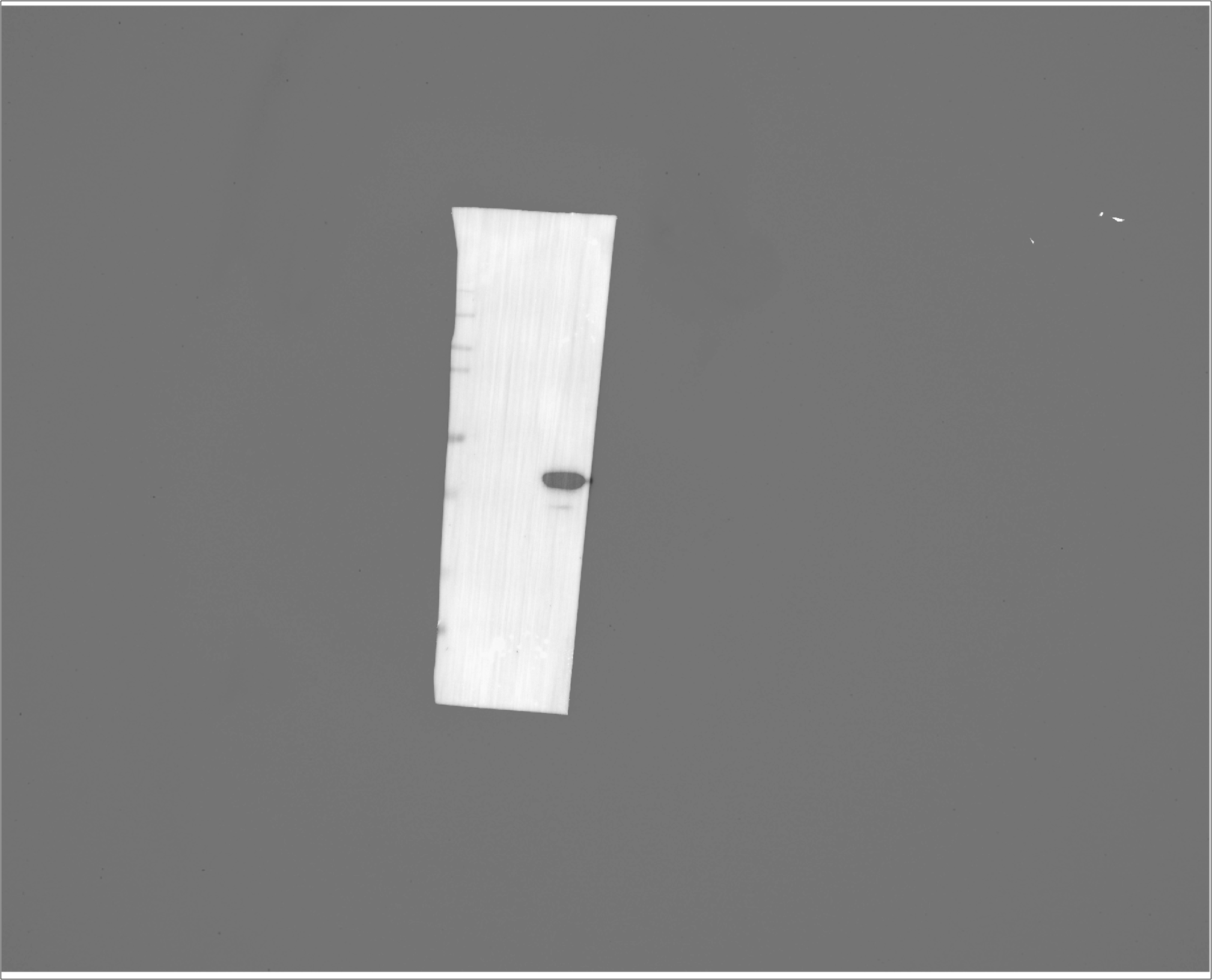

Supplement: Figure 5—figure supplement 1—source data 2. [file elife-107538-fig5-figsupp1-data2.zip › Figure 5- figure supplement 1C source data 2/ZMAT3-FLAG-HA.tif]

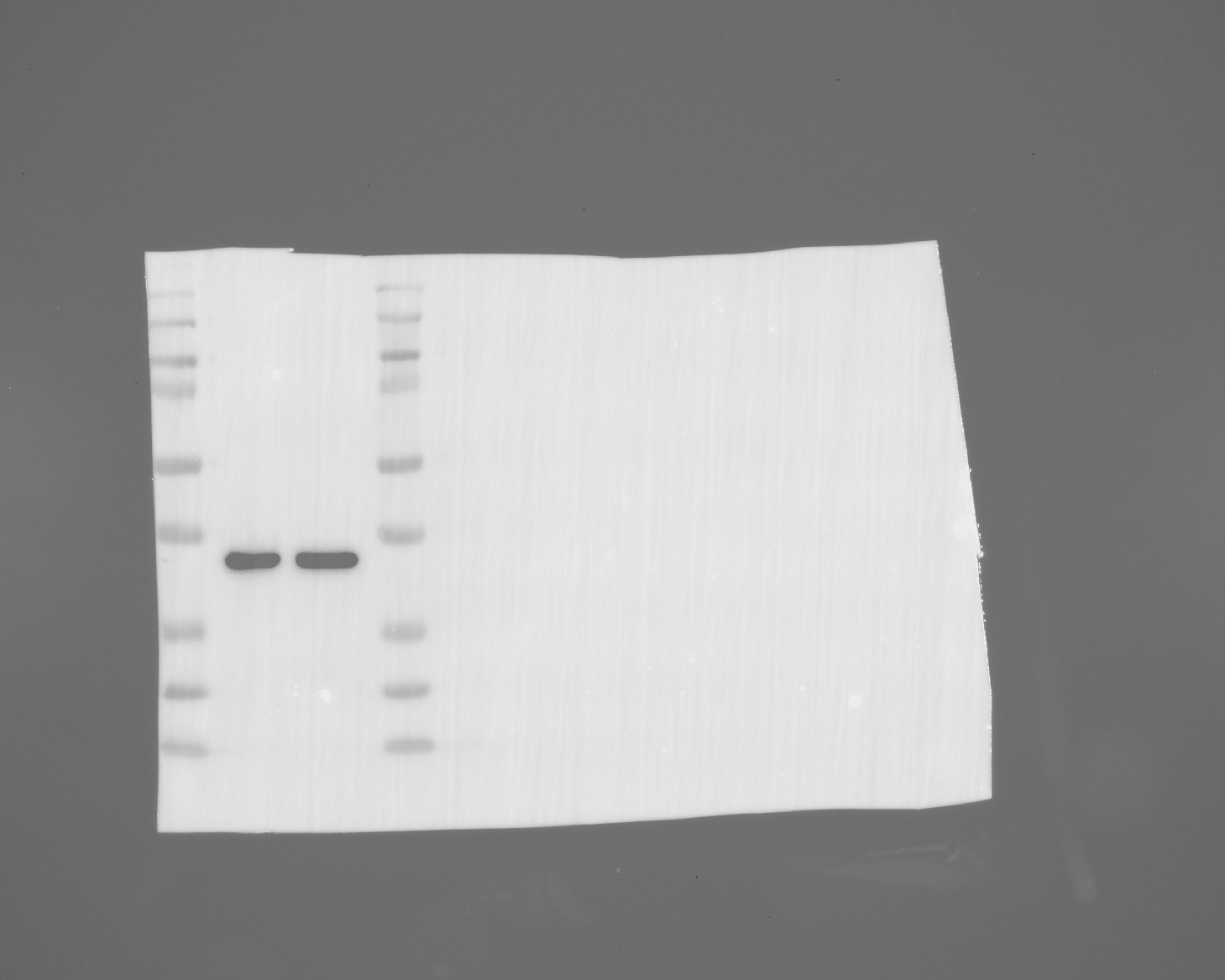

Supplement: Figure 5—figure supplement 1—source data 2. [file elife-107538-fig5-figsupp1-data2.zip › Figure 5- figure supplement 1D source data 2/GAPDH.tif]

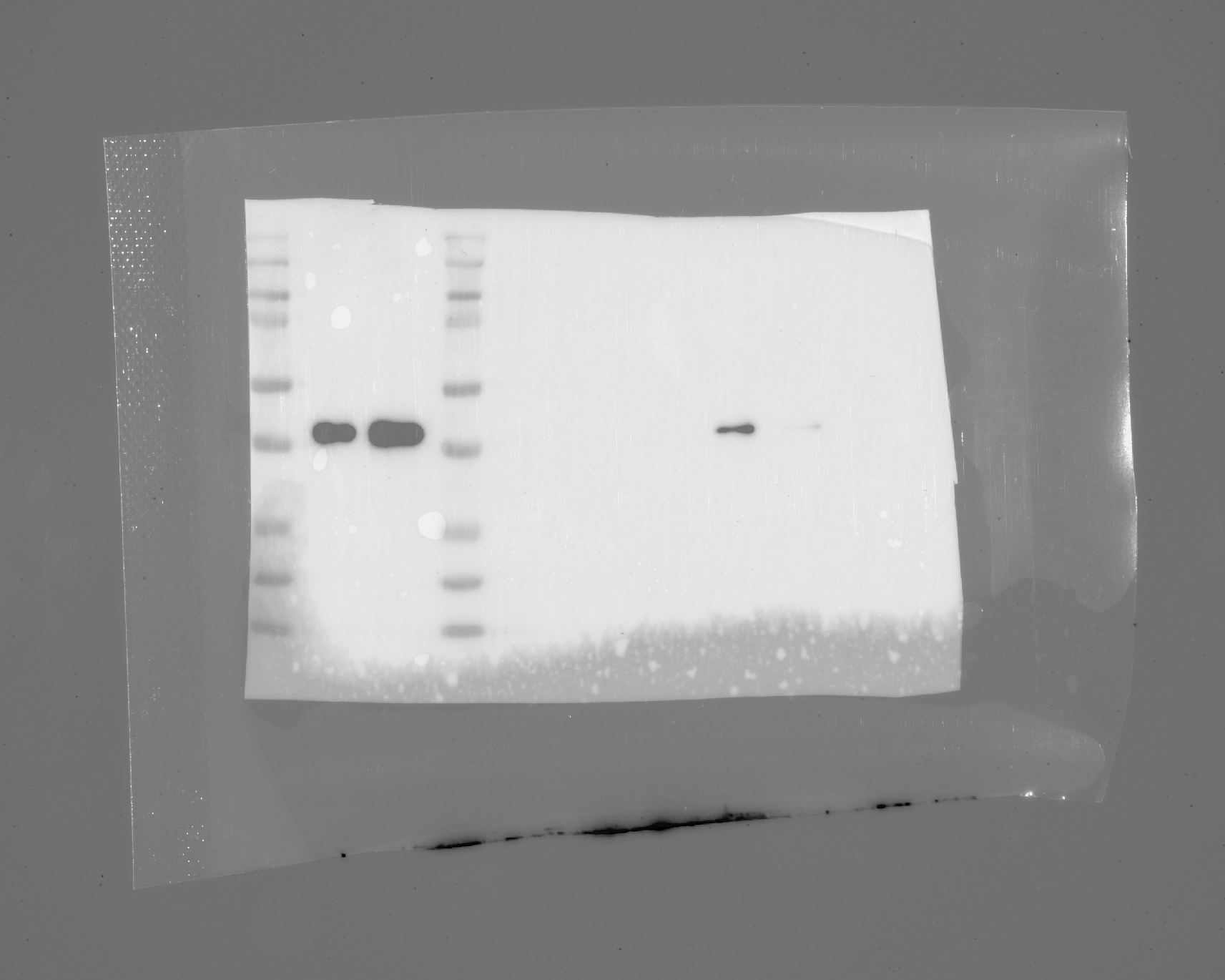

Supplement: Figure 5—figure supplement 1—source data 2. [file elife-107538-fig5-figsupp1-data2.zip › Figure 5- figure supplement 1D source data 2/JUN.tif]

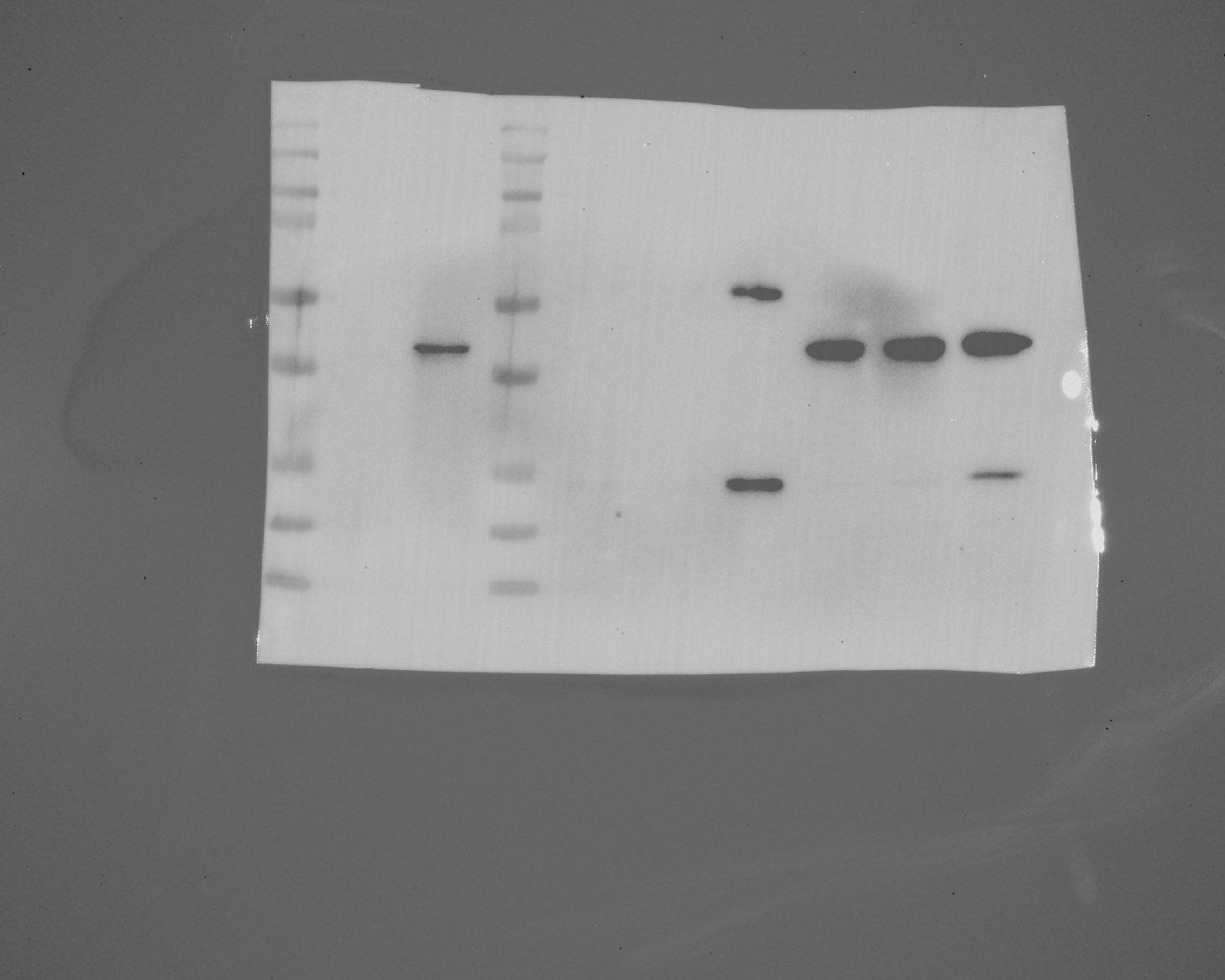

Supplement: Figure 5—figure supplement 1—source data 2. [file elife-107538-fig5-figsupp1-data2.zip › Figure 5- figure supplement 1D source data 2/ZMAT3-FLAG-HA.tif]
