# Supplementary material for: p53-induced RNA-binding protein ZMAT3 inhibits transcription of a hexokinase to suppress mitochondrial respiration in human cancer cells
Source: eLife. 2026 Mar 17;14:RP107538. doi: 10.7554/eLife.107538 (PMC12995290; doi:10.7554/eLife.107538)
Supplement: Figure 1—figure supplement 2—source data 1. [file elife-107538-fig1-figsupp2-data1.zip › Figure_1-figure_supplement_2-source_data_1.pdf]

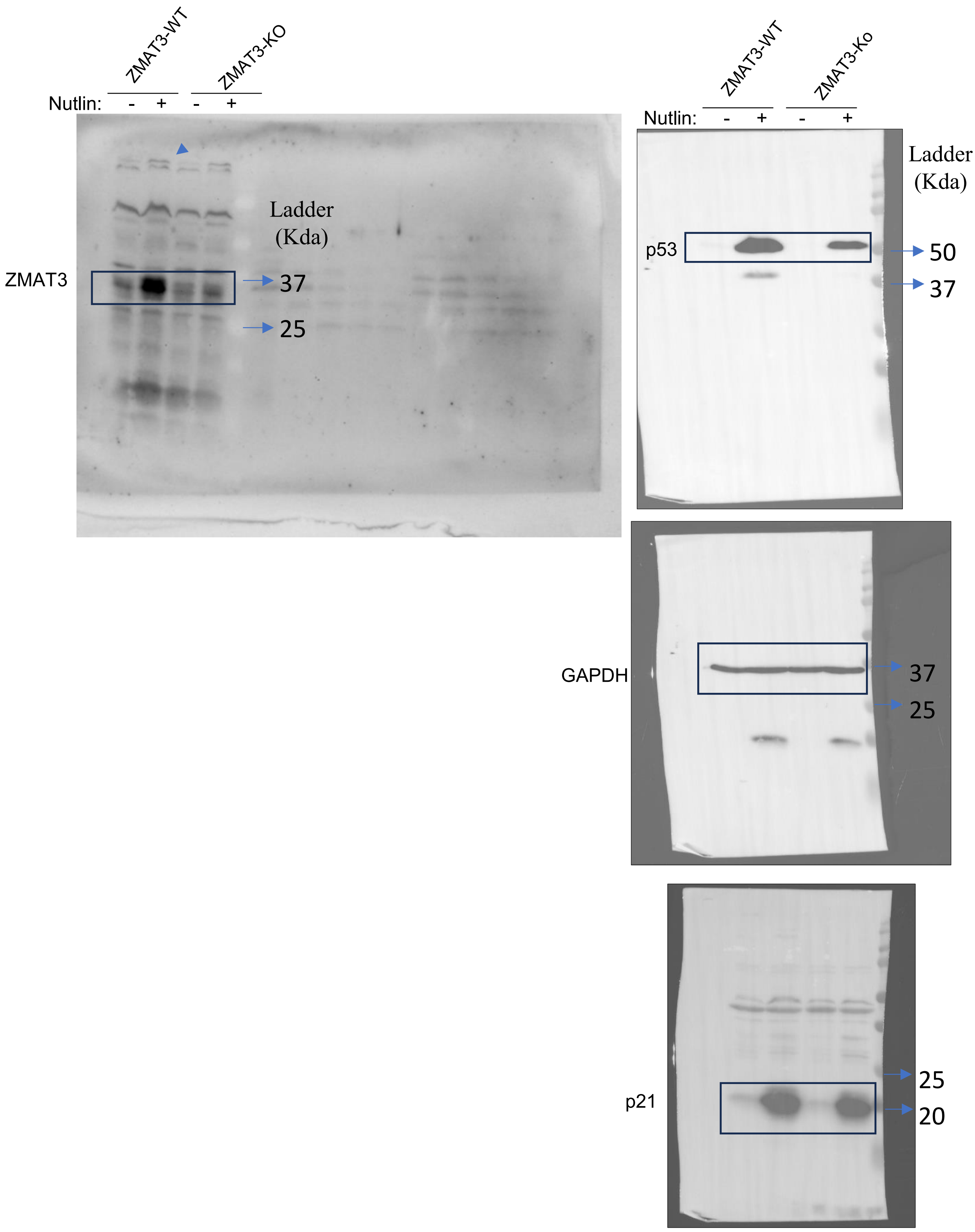

Figure 1-figure supplement 2-source data 1. Original membranes corresponding to Figure 1- figure supplement 2 panel A. BIO-RAD molecular markers (catalog no. 161-0394) were employed. The membranes correspond to ZMAT3, p53, p21 and GAPDH immunoblot.

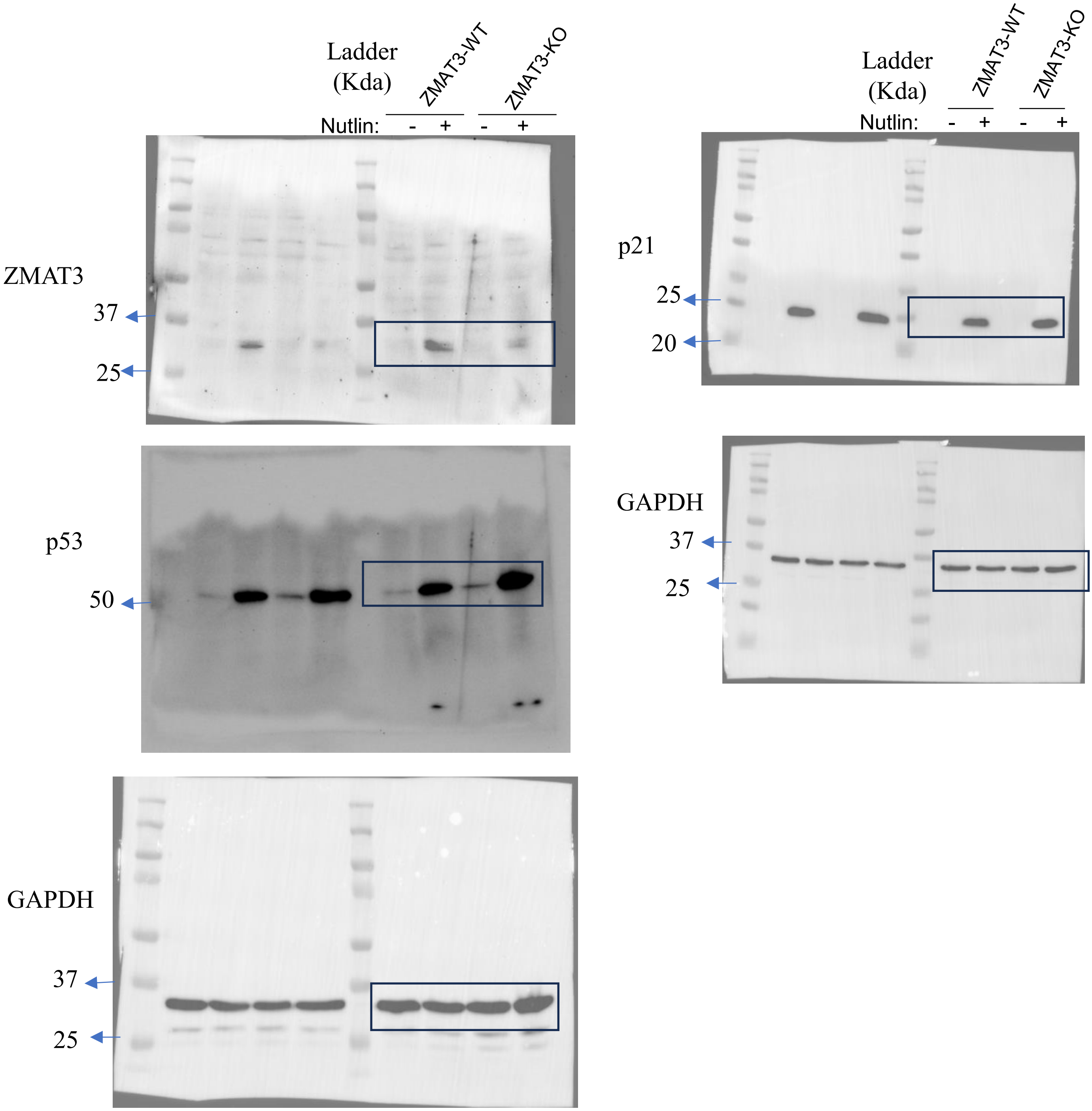

Figure 1- figure supplement 2-source data 1. Original membranes corresponding to Figure 1- figure supplement 2 panel B. BIO-RAD molecular markers (catalog no. 161-0394) were employed. The membranes correspond to ZMAT3, p53, p21 and GAPDH immunoblot.
