# Supplementary material for: p53-induced RNA-binding protein ZMAT3 inhibits transcription of a hexokinase to suppress mitochondrial respiration in human cancer cells
Source: eLife. 2026 Mar 17;14:RP107538. doi: 10.7554/eLife.107538 (PMC12995290; doi:10.7554/eLife.107538)
Supplement: Figure 2—source data 1. [file elife-107538-fig2-data1.zip › Figure_2-source_data_1.pdf]

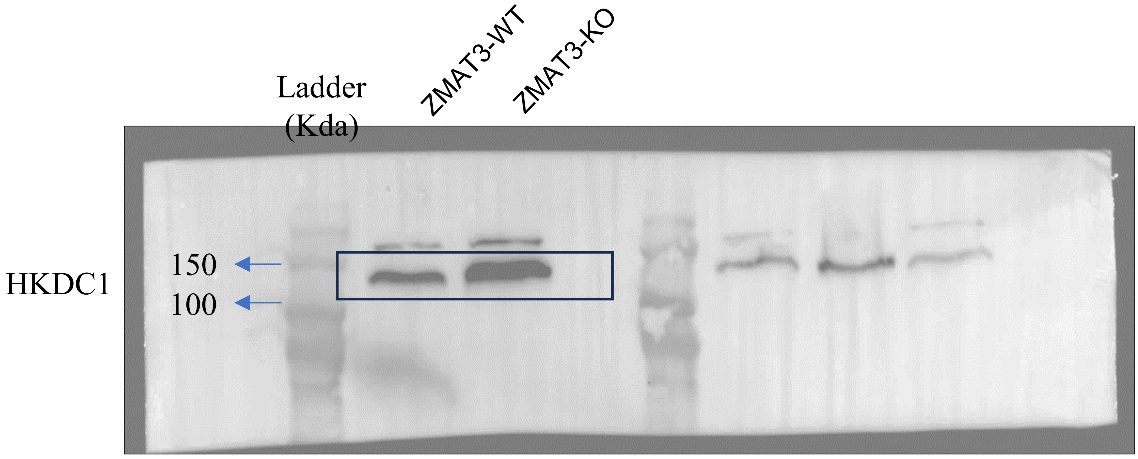

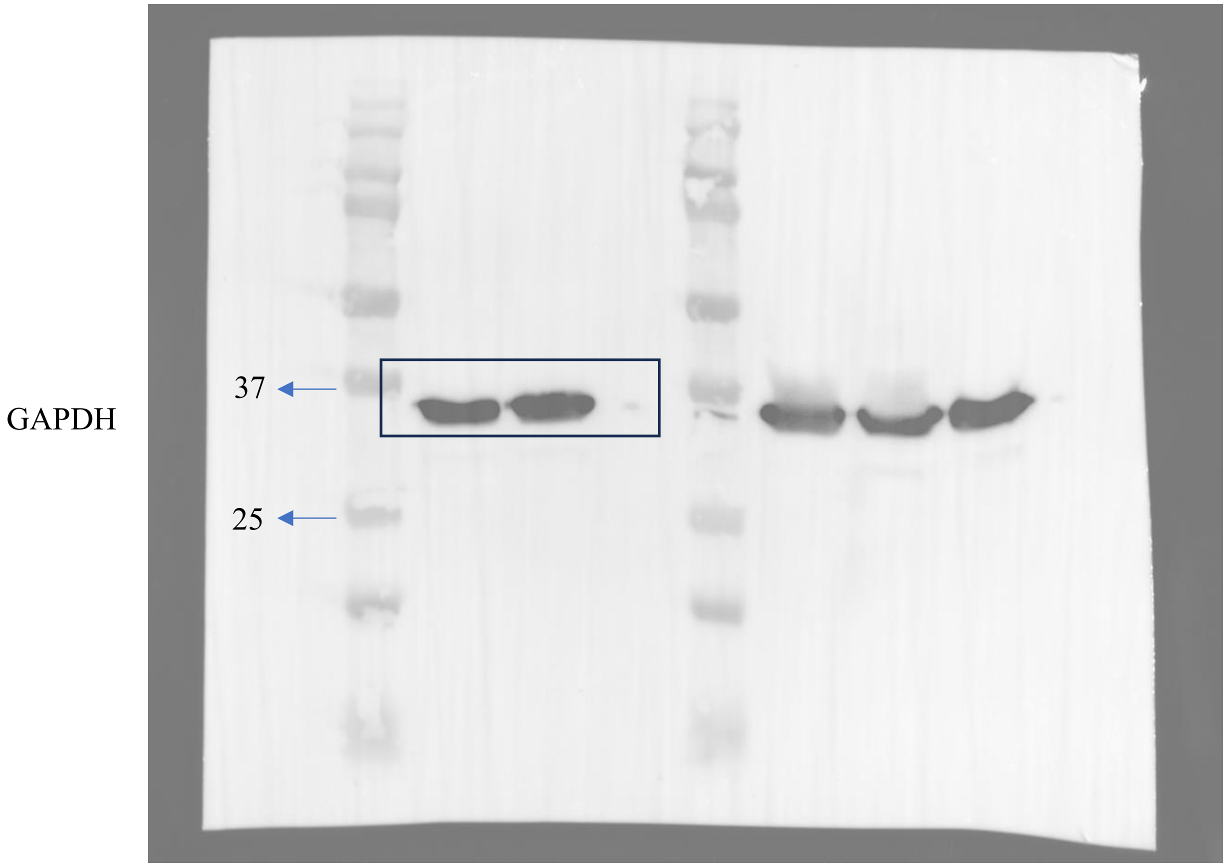

Figure 2-source data 1. Original membrane corresponding to Figure 2, panel B. BIO-RAD molecular markers (catalog no. 161-0394) were employed. The upper membrane corresponds to HKDC1 and lower membrane corresponds to GAPDH immunoblot.

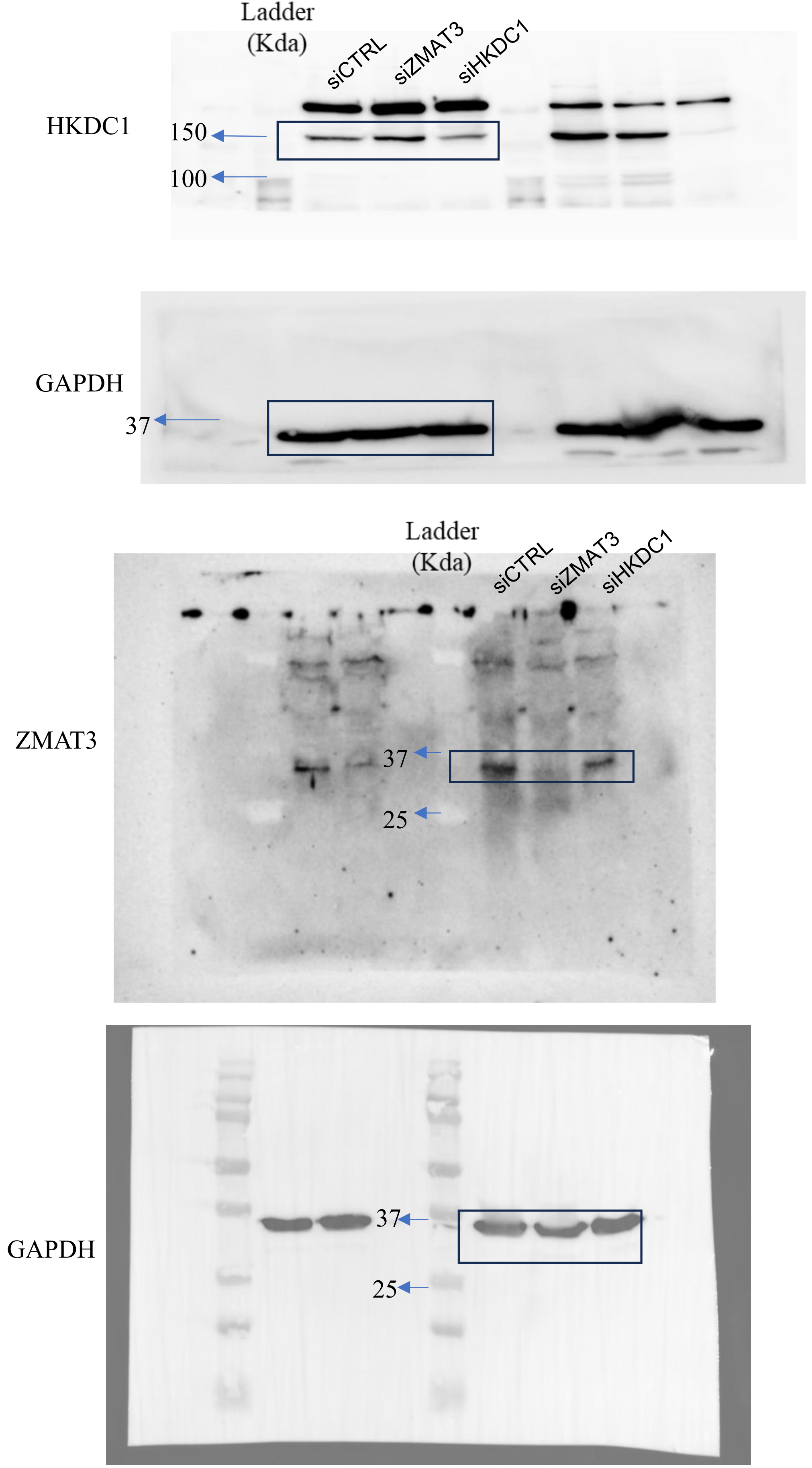

Figure 2-source data 1. Original membranes corresponding to Figure 2, panel E left for HCT116 cells. BIO-RAD molecular markers (catalog no. 161-0394) were employed. The upper two membranes corresponds to HKDC1 and GAPDH, and lower two membranes correspond to ZMAT3 and GAPDH immunoblot.

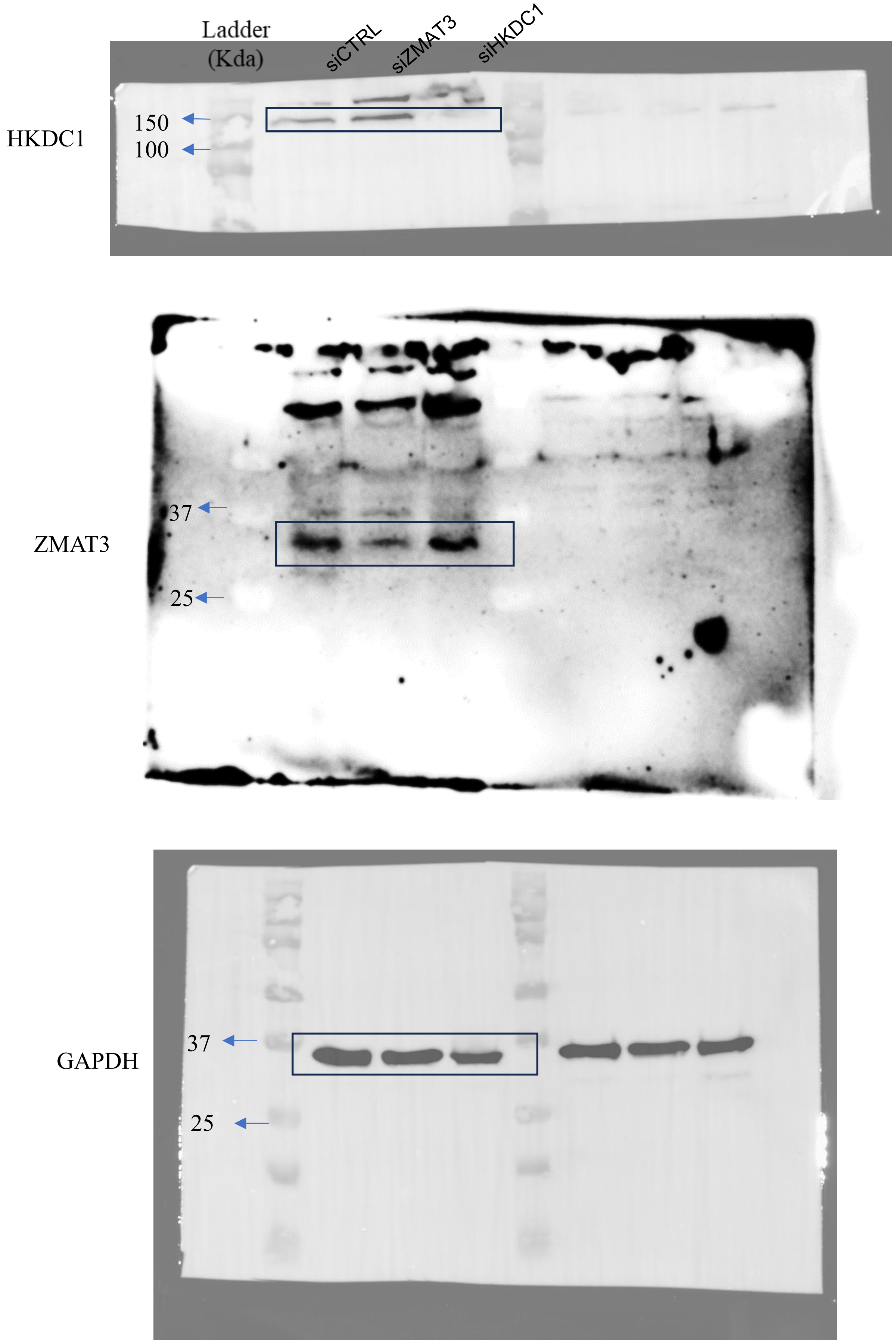

Figure 2-source data 1. Original membrane corresponding to Figure 2, panel E right for HepG2 cells. BIO-RAD molecular markers (catalog no. 161-0394) were employed. The membranes correspond to HKDC1, ZMAT3 and GAPDH immunoblot.
