# Supplementary material for: p53-induced RNA-binding protein ZMAT3 inhibits transcription of a hexokinase to suppress mitochondrial respiration in human cancer cells
Source: eLife. 2026 Mar 17;14:RP107538. doi: 10.7554/eLife.107538 (PMC12995290; doi:10.7554/eLife.107538)
Supplement: Figure 2—figure supplement 1—source data 1. [file elife-107538-fig2-figsupp1-data1.zip › Figure_2-_figure_supplement_1-source_data_1.pdf]

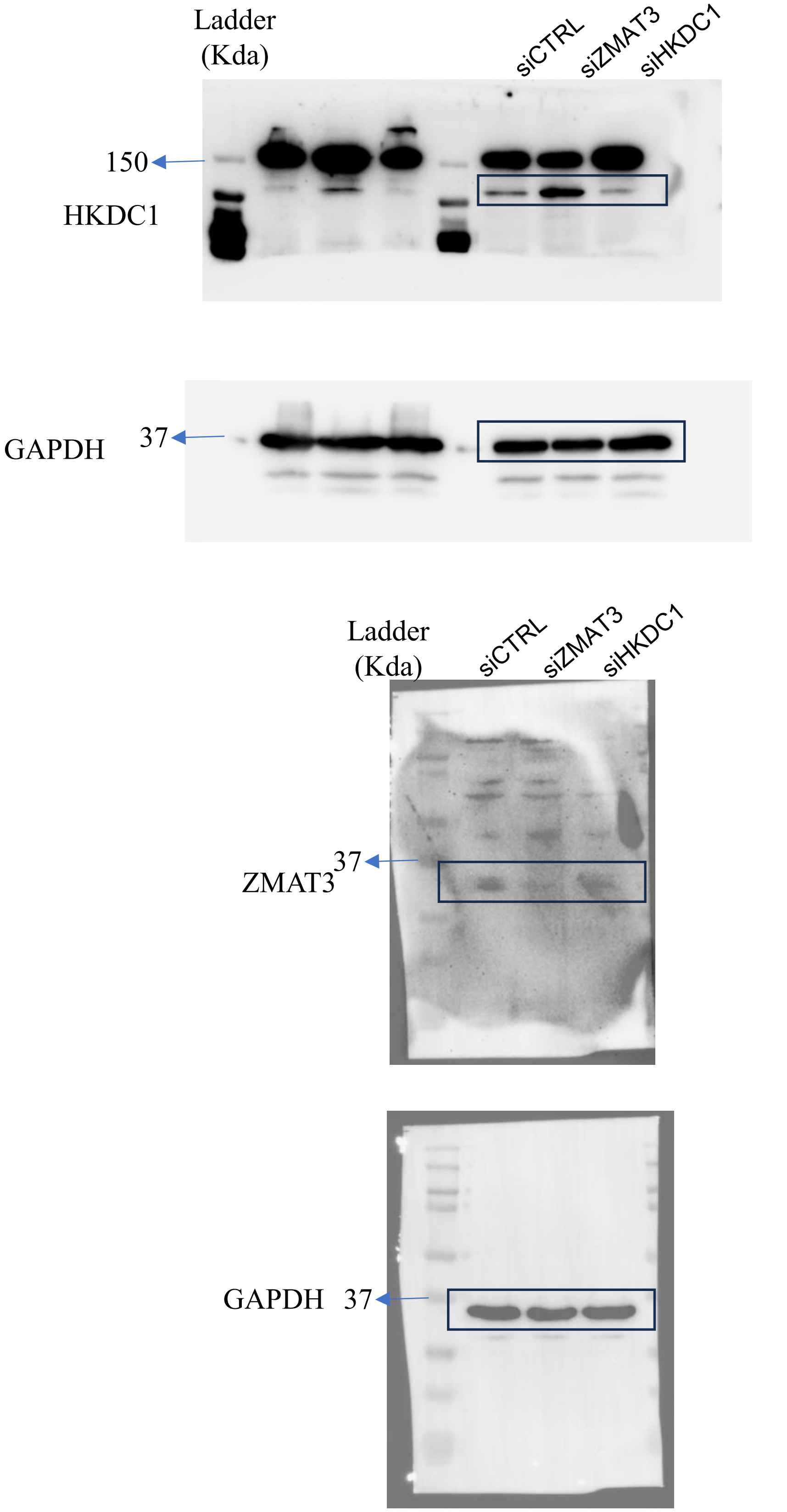

Figure 2- figure supplement 1-source data 1. Original membranes corresponding to Figure 2 - figure supplement 1, panel F. BIO-RAD molecular markers (catalog no. 161-0394) were employed. The membranes correspond to HKDC1, ZMAT3 and GAPDH immunoblot.

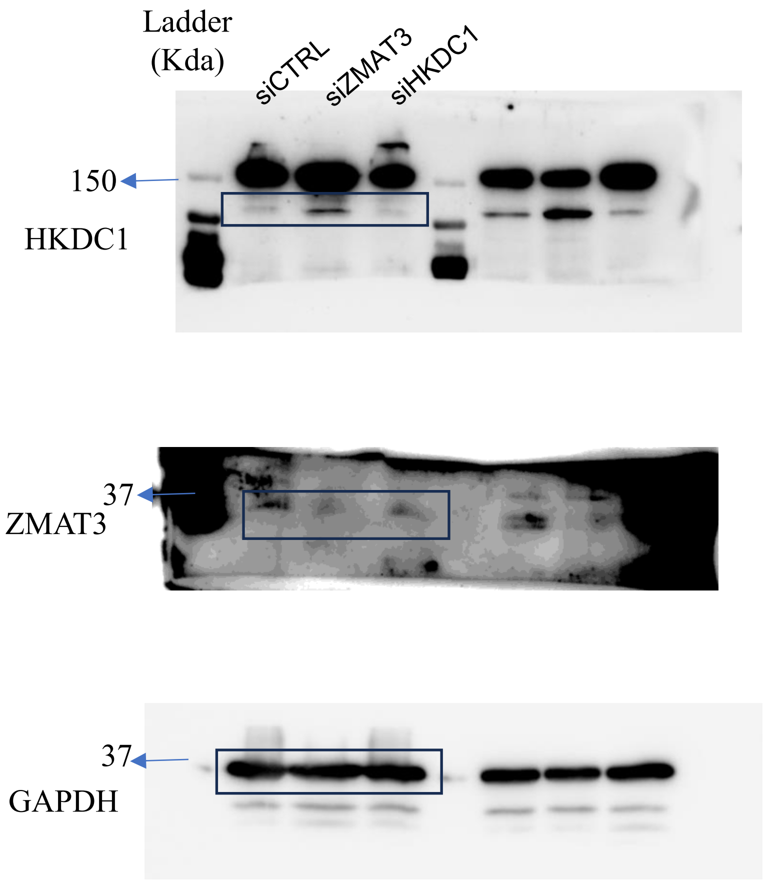

Figure 2- figure supplement 1-source data 1. Original membrane corresponding to Figure 2 - figure supplement 1, panel G. BIO-RAD molecular markers (catalog no. 161-0394) were employed. The membrane corresponds to HKDC1, ZMAT3 and GAPDH immunoblot.
